# Comparative Safety of Empirical Antibiotic Classes in Newly Hospitalized COVID-19 Patients

**DOI:** 10.3390/ph18101588

**Published:** 2025-10-21

**Authors:** Kalynn Park, Sohyeon Park, Jung Yoon Choi, Chaeyoon Kim, Jeongha Yun, Jiyeon Bae, Ji Yun Bae, Kang-Il Jun, Jeong-Han Kim, Chung-Jong Kim, Hee Jung Choi, Sandy Jeong Rhie

**Affiliations:** 1 Graduate School of Pharmaceutical Sciences, Ewha Womans University, Seoul 03760, Republic of Korea; ngpark93@ewha.ac.kr (K.P.); qkrthgus9129@ewha.ac.kr (S.P.); jungyoonchoi@ewhain.net (J.Y.C.); miseong@ewha.ac.kr (C.K.); jeongha.yun@ewha.ac.kr (J.Y.); 2 Division of Infectious Diseases, Department of Internal Medicine, Ewha Womans University Mokdong Hospital, Seoul 07985, Republic of Korea; jiyeonbae@ewha.ac.kr (J.B.); 01294@eumc.ac.kr (J.-H.K.); 3 Division of Infectious Diseases, Department of Internal Medicine, Ewha Womans University Seoul Hospital, Seoul 07804, Republic of Korea; 00979@eumc.ac.kr (J.Y.B.); 01102s@eumc.ac.kr (K.-I.J.); erinus@ewha.ac.kr (C.-J.K.)

**Keywords:** coronavirus disease 2019, anti-bacterial agents, mortality, real-world data

## Abstract

**Background**: Empirical antibiotic use is common in hospitalized patients with COVID-19 despite the low prevalence of bacterial coinfection, raising concerns about antimicrobial resistance and inappropriate prescribing. However, the comparative safety of commonly used antibiotic classes in this context remains unclear. **Methods**: We conducted a retrospective cohort study using real-world clinical data standardized through the Observational Medical Outcomes Partnership Common Data Model from 1 January 2020 to 31 May 2025. Adults with confirmed COVID-19 who were administered empirical antibiotics on the admission day were included. Empirical antibiotic exposure was categorized as third-generation cephalosporins (3GCs), fluoroquinolones, or aminopenicillins with β-lactamase inhibitors (PEN–BLis). **Results**: Compared with 3GCs, fluoroquinolone use was associated with significantly higher risks of mechanical ventilation (hazard ratio [HR]: 1.50; 95% confidence interval [CI]: 1.12–2.00), ICU admission (HR: 1.54; 95% CI: 1.10–2.15), vasopressor use (HR: 1.35; 95% CI: 1.11–1.63), all-cause in-hospital mortality (HR: 1.55; 95% CI: 1.22–1.96), and the composite outcome (HR: 1.32; 95% CI: 1.10–1.60). PEN–BLis showed no significant differences from 3GCs across outcomes. **Conclusions**: Empirical fluoroquinolone use at COVID-19 admission may be associated with greater risks of critical care interventions and in-hospital mortality compared to those of 3GCs. These findings highlight the need for careful patient selection and clinical judgment when initiating empirical antibiotic therapy for viral respiratory infections such as COVID-19.

## 1. Introduction

The COVID-19 pandemic has created unprecedented challenges in clinical decision-making, including widespread empirical antibiotic use without confirmed bacterial coinfection [1,2]. Early in the pandemic, up to 75% of hospitalized patients with COVID-19 were prescribed antibiotics despite the relatively low prevalence of documented bacterial infections, reflecting concerns regarding potential secondary infections and the initial lack of antiviral therapies [1,2,3]. However, such extensive prescribing has raised concerns about antibiotic overuse and its role in antimicrobial resistance, particularly when empirical regimens deviate from guideline recommendations.

Commonly prescribed antibiotics for community-acquired respiratory infections include third-generation cephalosporins (3GCs), fluoroquinolones, and aminopenicillins with β-lactamase inhibitors (PEN–BLis) such as amoxicillin–clavulanate and ampicillin–sulbactam [2,4]. For adults admitted to general wards with mild-to-moderate community-acquired pneumonia, the Korean guidelines recommend initial empiric therapy with either a β-lactam or respiratory fluoroquinolone [5]. When aspiration pneumonia is suspected, or infection with β-lactamase–producing organisms is a concern, penicillin/β-lactamase inhibitor combinations (e.g., ampicillin–sulbactam or amoxicillin–clavulanate) are often used while awaiting microbiological results [6]. Nevertheless, their roles in managing viral pneumonia, such as COVID-19, remain unclear [7,8]. Without specific clinical guidelines, antibiotic selection often reflects institutional practice, perceived illness severity, or patient characteristics rather than microbiological evidence or anticipated benefit [9,10]. Consequently, predicting patient outcomes based on empirical antibiotic choice in COVID-19 remains challenging.

Although many studies report antibiotic prescribing rates in COVID-19, few have directly compared patient outcomes across antibiotic classes, especially in real-world, multi-institutional cohorts. Clarifying how specific empirical antibiotic choices correlate with clinical outcomes may support more judicious prescribing and guide future preparedness for viral respiratory outbreaks and pandemics.

This study aims to evaluate the short-term clinical outcomes in newly admitted patients with COVID-19 who received empirical treatment with fluoroquinolones or PEN–BLis, compared to those treated with 3GCs. We specifically assessed whether the initial empirical antibiotic choice was associated with critical in-hospital outcomes, including mechanical ventilation initiation, intensive care unit (ICU) admission, vasopressor use, a composite outcome of these three outcomes, and all-cause in-hospital mortality.

## 2. Results

### 2.1. Cohort Construction and Propensity Score Adjustment

We identified adult patients (≥18 years) hospitalized with COVID-19 between J1 2020, and 31 May 2025, across 12 tertiary hospitals in South Korea. Patients who received empirical antibiotic therapy with 3GCs, fluoroquinolones, or PEN–BLis on admission day were included, with data standardized to the Observational Medical Outcomes Partnership Common Data Model (OMOP-CDM) [11,12].

**Figure 1:**
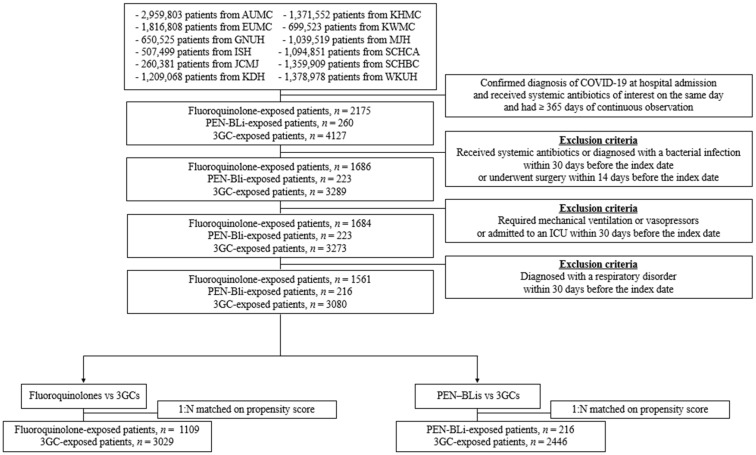
Cohort construction and patient distribution following propensity score matching for antibiotic comparisons. Matched cohorts were constructed independently. 3GCs: Third-generation cephalosporins; AUMC: Ajou University Medical Center; EUMC: Ewha Womans University Medical Center; GNUH: Gyeongsang National University Hospital; ISH: International St. Mary’s Hospital; JCMJ: Jecheon Myongji Hospital; KDH: Kangdong Sacred Heart Hospital; KHMC: Kyunghee University Medical Center; KWMC: Kangwon National University Medical Center; MJH: Myongji Hospital; PEN–BLis: aminopenicillin/β-lactamase inhibitor combinations; SCHCA: Soonchunhyang University Hospital Cheonan Center; SCHBC: Soonchunhyang University Hospital Bucheon Center; WKUH: Wonkwang University Hospital.

To adjust baseline differences, we employed large-scale propensity score (PS) methods using regularized logistic regression incorporating a comprehensive set of demographics, clinical, and laboratory variables. Primary analyses were conducted using PS matching. In the fluoroquinolone versus 3GC comparison, the matched cohorts included 1505 and 3050 patients, respectively. In the PEN–BLi versus 3GC comparison, the matched cohorts comprised 216 PEN–BLi and 2446 3GC patients (Figure 1). Although overall covariate balance improved after matching, residual imbalances (standardized mean difference > 0.1) persisted for age, sex, and certain comorbidities in specific comparisons. Supplementary Tables S1A,B–S9A,B, S10A, S11A and S12A,B shows the baseline characteristics before and after PS adjustment, with Supplementary Figures S1A,B–S9A,B, S10A, S11A and S12A,B showing love plots of standardized mean differences.

### 2.2. Primary Outcomes by Antibiotic Class

**Figure 2:**
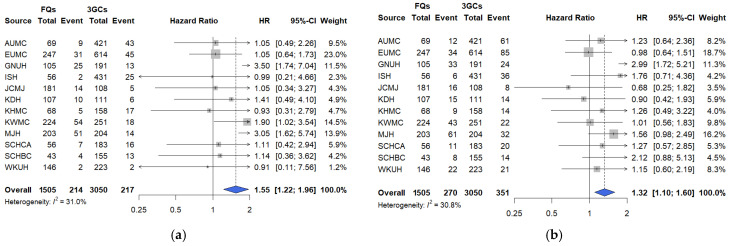
Comparative risks of primary outcomes between fluoroquinolones and 3GCs among hospitalized patients with COVID-19. (**a**) All-cause in-hospital mortality. (**b**) Composite outcome. The number of events and total patients in each treatment group are shown alongside site-specific hazard ratios (HRs). The HRs were calibrated based on the empirical null distribution derived from negative control outcomes to account for systematic bias. The size of the data marker indicates the weight of the study. Error bars indicate 95% CIs. 3GCs: Third-generation cephalosporins; AUMC: Ajou University Medical Center; CI: confidence interval; EUMC: Ewha Womans University Medical Center; FQs: Fluoroquinolones; GNUH: Gyeongsang National University Hospital; ISH: International St. Mary’s Hospital; JCMJ: Jecheon Myongji Hospital; KDH: Kangdong Sacred Heart Hospital; KHMC: Kyunghee University Medical Center; KWMC: Kangwon National University Medical Center; MJH: Myongji Hospital; SCHCA: Soonchunhyang University Hospital Cheonan Center; SCHBC: Soonchunhyang University Hospital Bucheon Center; WKUH: Wonkwang University Hospital.

We evaluated all-cause in-hospital mortality and a composite outcome, which is defined as the occurrence of mechanical ventilation, ICU admission, or vasopressor use within the time-at-risk window. In PS-matched cohorts, fluoroquinolone use was associated with statistically significant increases in both primary endpoints relative to those of 3GCs (mortality: hazard ratio [HR] 1.55, 95% confidence interval [CI] 1.22–1.96; composite: HR 1.32, 95% CI 1.10–1.60). This indicates elevated risks with fluoroquinolone therapy (Figure 2). Hospital-level heterogeneity was low to moderate (mortality: I^2^ = 31.0%; composite: I^2^ = 30.0%), with consistent risk elevations associated with fluoroquinolone therapy relative to 3GCs across institutions. The minimum detectable HRs (80% power) were 1.33 and 1.27 for mortality and the composite outcome, respectively.

Cumulative incidence curves showed higher event rates among fluoroquinolone users than GC users for all-cause in-hospital mortality and the composite outcome (Supplementary Figure S13A,B). Separation between groups was most pronounced in the first week of admission for the composite outcome, whereas mortality curves diverged more gradually. These trends were consistent across hospitals.

**Figure 3:**
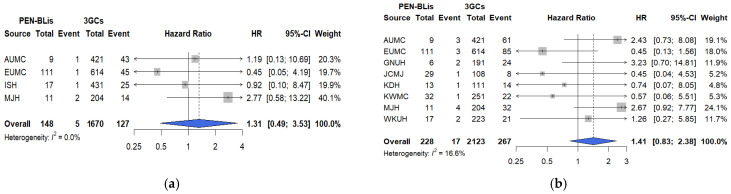
Comparative risks of primary outcomes between PEN–BLis and 3GCs among hospitalized patients with COVID-19. (**a**) All-cause in-hospital mortality. (**b**) Composite severe outcome. The number of events and total patients in each treatment group are shown alongside site-specific hazard ratios (HRs). The HRs were calibrated based on the empirical null distribution derived from negative control outcomes to account for systematic bias. The size of the data marker indicates the weight of the study. Error bars indicate 95% CIs. 3GCs: Third-generation cephalosporins; AUMC: Ajou University Medical Center; CI: confidence interval; EUMC: Ewha Womans University Medical Center; GNUH: Gyeongsang National University Hospital; ISH: International St. Mary’s Hospital; JCMJ: Jecheon Myongji Hospital; KDH: Kangdong Sacred Heart Hospital; KWMC: Kangwon National University Medical Center; MJH: Myongji Hospital; PEN–BLis: aminopenicillin/β-lactamase inhibitor combinations; WKUH: Wonkwang University Hospital.

In the PEN–BLi versus 3GC comparison, no statistically significant differences were observed (Figure 3). For all-cause in-hospital mortality, the pooled HR was 1.31 (95% CI: 0.49–3.53; I^2^ = 0.0%) (Figure 3a), and for the composite outcome, 1.41 (95% CI: 0.83–2.38; I^2^ = 16.6%) (Figure 3b). Heterogeneity remained low (mortality: I^2^ = 0.0%; composite: I^2^ = 16.6%). Although small sample sizes and low event rates produced wide CIs, point estimates for all outcomes suggested higher risks with PEN–BLis. Detection thresholds were higher for this comparison: 2.44 for mortality and 1.75 for the composite outcome.

Cumulative incidence curves showed higher early-stage event rates among PEN–BLi users, but overall trends closely mirrored those of the 3GC users (Supplementary Figure S14A,B).

### 2.3. Secondary Outcomes by Antibiotic Class

**Figure 4:**
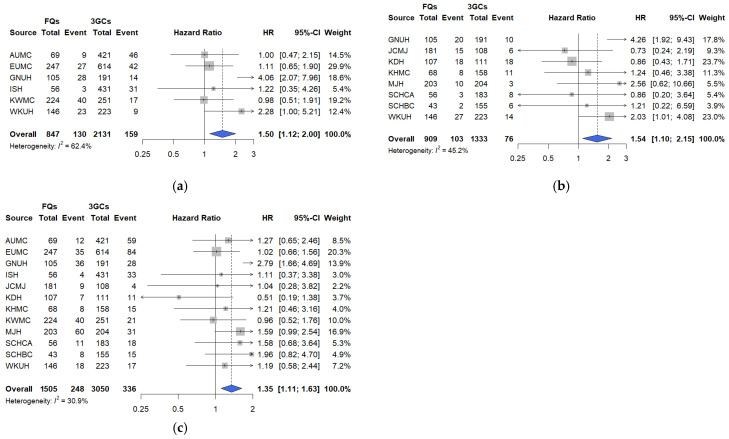
Comparative risks of secondary outcomes between fluoroquinolones and 3GCs among hospitalized patients with COVID-19. (**a**) Mechanical ventilation. (**b**) Intensive Care Unit (ICU) admission. (**c**) Vasopressor use. The number of events and total patients in each treatment group are shown alongside site-specific hazard ratios (HRs). The HRs were calibrated based on the empirical null distribution derived from negative control outcomes to account for systematic bias. The size of the data marker indicates the weight of the study. Error bars indicate 95% CIs. 3GCs, Third-generation cephalosporins; AUMC: Ajou University Medical Center; CI: confidence interval; EUMC: Ewha Womans University Medical Center; FQs: Fluoroquinolones; GNUH: Gyeongsang National University Hospital; ISH: International St. Mary’s Hospital; JCMJ: Jecheon Myongji Hospital; KDH: Kangdong Sacred Heart Hospital; KHMC: Kyunghee University Medical Center; KWMC: Kangwon National University Medical Center; MJH: Myongji Hospital; SCHCA: Soonchunhyang University Hospital Cheonan Center; SCHBC: Soonchunhyang University Hospital Bucheon Center; WKUH: Wonkwang University Hospital.

Fluoroquinolone use was associated with higher risks of mechanical ventilation (HR: 1.50, 95% CI: 1.12–2.00; Figure 4a), ICU admission (HR: 1.54, 95% CI: 1.10–2.15; Figure 4b), and vasopressor use (HR: 1.35, 95% CI: 1.11–1.63; Figure 4c) than 3GCs. Hospital heterogeneity was moderate (I^2^ 30.9–62.4%). The cumulative incidence curves for these secondary outcomes aligned with the primary findings, demonstrating early separation within the first week of admission and consistent patterns across all participating hospitals (Supplementary Figure S15A–C).

**Figure 5:**
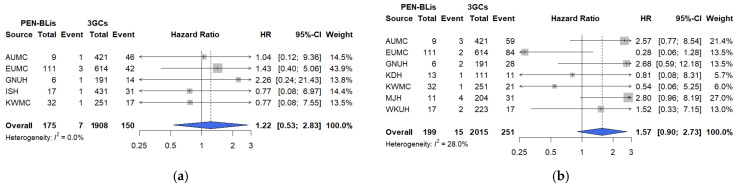
Comparative risks of secondary outcomes between PEN–BLis and 3GCs among hospitalized patients with COVID-19. (**a**) Mechanical ventilation. (**b**) Vasopressor use. The number of events and total patients in each treatment group are shown alongside site-specific hazard ratios (HRs). The HRs were calibrated based on the empirical null distribution derived from negative control outcomes to account for systematic bias. The size of the data marker indicates the weight of the study. Error bars indicate 95% CIs. 3GCs: Third-generation cephalosporins; AUMC: Ajou University Medical Center; CI: confidence interval; EUMC: Ewha Womans University Medical Center; GNUH: Gyeongsang National University Hospital; ISH: International St. Mary’s Hospital; JCMJ: Jecheon Myongji Hospital; KDH: Kangdong Sacred Heart Hospital; KWMC: Kangwon National University Medical Center; MJH: Myongji Hospital; PEN–BLis: aminopenicillin/β-lactamase inhibitor combinations; WKUH: Wonkwang University Hospital.

The risk of mechanical ventilation did not differ significantly between PEN–BLi and 3GC users (HR: 1.22, 95% CI: 0.55–2.83, I^2^ = 0.0%; Figure 5a). Vasopressor use was higher among PEN–BLi users than with 3GC users (HR: 1.57, 95% CI: 0.90–2.73; I^2^ = 28.0%), though the difference was not statistically significant (Figure 5b). Consistent with the findings of the primary analyses, the minimum detectable HRs (80% power) in fluoroquinolones vs. 3GCs were 1.44 (mechanical ventilation), 1.53 (ICU admission), and 1.28 (vasopressor use); in PEN–BLis vs. 3GCs, the values were 2.24 and 1.82, respectively. The cumulative incidence curves for PEN–BLi users mirrored the primary endpoints, showing higher early-phase event rates, but the overall trends were similar to those of 3GC users (Supplementary Figure S16A,B).

### 2.4. Sensitivity Analysis

Using a severity-augmented PS incorporating baseline vital signs, laboratory parameters, and oxygenation (peripheral oxygen saturation by pulse oximetry [SpO_2_], arterial oxygen saturation [SaO_2_]), results aligned with the main analysis: fluoroquinolones carried significantly higher risks than 3GCs across all evaluated outcomes, whereas PEN–BLis showed higher but non-significant risks than 3GCs across outcomes (Supplementary Figures S17A,B and S18A,B). The distributions of all variables before and after propensity-score adjustment are provided in Supplementary Tables S13A,B–S19A,B, S20A, S21A and S22A,B.

In a sensitivity analysis excluding patients with early microbiological confirmation, PCT ≥ 2 ng/mL, the associations remained consistent with the main outcomes. Fluoroquinolones were linked to higher risks for both primary outcomes—all-cause in-hospital mortality and composite outcome—relative to those of 3GCs (Supplementary Figure S19A). In contrast, PEN–BLis showed similarly elevated but non-significant risks for these outcomes relative to those of 3GCs, which is consistent with findings from the main analysis (Supplementary Figure S19B). For the secondary outcomes, fluoroquinolones were associated with significantly increased risks of ICU admission and vasopressor use, while the hazard for mechanical ventilation was numerically higher but not statistically significant (Supplementary Figure S20A). In contrast, PEN–BLis demonstrated numerically higher but non-significant HRs for these outcomes (Supplementary Figure S20B).

In sensitivity analyses restricted to the Omicron period, the associations were consistent with the primary findings. Fluoroquinolones were associated with significantly higher risks for both primary outcomes—all-cause in-hospital mortality and the composite outcome, compared with 3GCs (Supplementary Figure S21A). Consistent with findings from the primary analysis, PEN–BLis demonstrated directionally different risks for all-cause mortality and the composite outcome, but remained statistically non-significant (Supplementary Figure S21B). For the secondary outcomes, fluoroquinolones demonstrated significantly greater risks of mechanical ventilation, ICU admission, and vasopressor use, consistent with findings from the primary analysis (Supplementary Figure S22A). PEN–BLis were associated with increased hazard for mechanical ventilation and reduced hazard for vasopressor use; this was directionally consistent with the primary analysis for mechanical ventilation but differed for vasopressor use, and both associations remained non-significant as in the primary analysis (Supplementary Figure S22B).

In a sensitivity analysis restricted to the most commonly prescribed agents, levofloxacin/moxifloxacin use was associated with higher risks for all outcomes than with ceftriaxone, including all-cause in-hospital mortality, composite outcome, mechanical ventilation, ICU admission, and vasopressor use (Supplementary Figures S23 and S24).

In the as-treated analysis, HRs were directionally consistent with findings from the main analysis; however, most were not statistically significant, except for mortality (Supplementary Figures S25A,B and S26A,B).

### 2.5. Quantitative Bias Analysis

**Figure 6:**
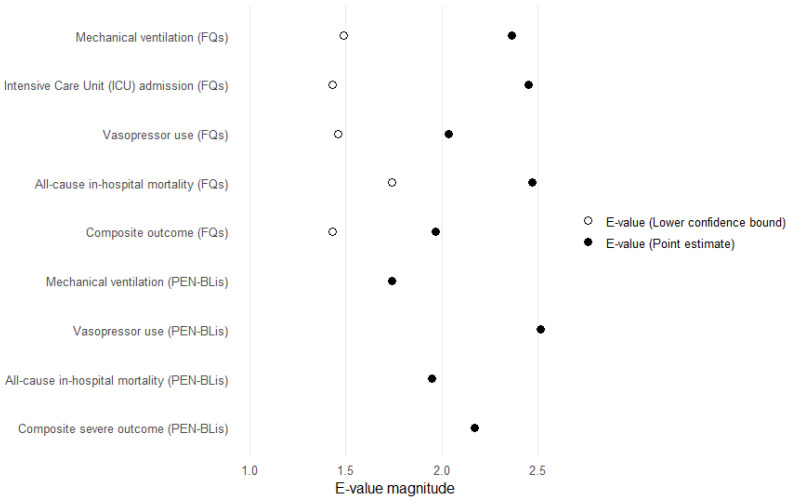
E-values for adverse outcomes associated with empirical antibiotic therapy in hospitalized patients with COVID-19. E-values quantifying the minimum strength of association that an unmeasured confounder would need to have with both antibiotic class (fluoroquinolones or PEN–BLis vs. 3GCs) and each outcome to fully explain away the observed effect. Filled circles denote E-values for the point estimates, and open circles denote E-values for the lower bounds of the 95% confidence intervals. 3GCs: Third-generation cephalosporins; PEN–BLis: aminopenicillin/β-lactamase inhibitor combinations.

E-value analysis indicated that fluoroquinolone findings would necessitate unmeasured confounding of considerable magnitude. For all-cause in-hospital mortality, the point estimate corresponded to an E-value of 2.47 (lower confidence bound, 1.74), suggesting that only a confounder with approximately 2.5-fold associations with exposure and outcome—beyond measured covariates—could fully account for the observed effect. E-values for mechanical ventilation, ICU admission, and vasopressor use were of similar magnitude, further supporting the robustness of these findings. In contrast, the PEN–BLis estimates were not statistically significant; therefore, CI-limit E-values could not be defined, and only point-estimate E-values are provided, which do not support a definitive association. Figure 6 illustrates a consolidated visualization.

## 3. Discussion

In this large, multicenter, real-world cohort study using the OMOP-CDM framework across 12 tertiary hospitals in South Korea, short-term clinical outcomes were compared among hospitalized patients with COVID-19 treated with three commonly prescribed empirical antibiotic classes—3GCs, fluoroquinolones, and PEN–BLis. Using rigorous PS adjustment through matching, this study revealed that empirical fluoroquinolone use was associated with significantly higher rates of key clinical outcomes, including mechanical ventilation, ICU admission, vasopressor use, all-cause in-hospital mortality, and a composite outcome, than with 3GCs use. These associations were robust across multiple analytical approaches. In contrast, PEN–BLi use demonstrated a higher but non-significant trend for the same outcomes than with 3GC use, suggesting a potentially elevated risk.

These findings contribute to the growing evidence on antibiotic use in patients with COVID-19. While previous studies primarily describe antibiotic overuse, few directly compared the safety of specific antibiotic classes. For example, a recent study reported that bacterial culture testing was conducted in only 27.1% of antibiotic-treated patients with COVID-19, with most prescriptions classified within the WHO AWaRe Watch category [13]. The SEMI-COVID study from Spain reports inappropriate antibiotic use in over one-third of hospitalized patients with COVID-19, which is associated with higher rates of adverse events, including *Clostridioides difficile* infection and drug-related toxicities [14]. Furthermore, Buetti et al. report no mortality benefit from previous antibiotic administration in critically ill patients [15], and Langford et al. report that >70% of patients with COVID-19 received antibiotics despite low bacterial coinfection rates [2]. Beyond COVID-19, evidence from viral pneumonia further contextualizes these findings. In population-based surveillance of community-acquired pneumonia requiring hospitalization, respiratory viruses are detected more frequently than bacterial pathogens. This indicates that empiric antibacterial therapy in this framework is often microbiologically unsubstantiated [16]. Among adults with PCR-confirmed viral respiratory infections without evidence of bacterial coinfection, antibiotic treatment fails to reduce 30-day mortality or length of hospital stay [17]. Collectively, these findings provide the broader framework for interpreting our results: when the underlying etiology is viral, routine antibacterial therapy rarely confers benefit, and the choice of antibiotic class may influence patient outcomes. While studies comparing antibiotic classes exist for bacterial infections [18,19], such direct comparisons are notably lacking regarding documented viral pneumonia. Therefore, our study advances the literature by applying a large-scale, multicenter, direct comparative design with rigorous confounding control. This approach demonstrates a new dimension beyond the general futility of antibiotic use in viral infections, revealing that fluoroquinolones are associated with increased short-term clinical risks compared with 3GCs.

The elevated risks associated with fluoroquinolone use may be partially explained by known pharmacologic and biologic properties. Fluoroquinolones induce mitochondrial dysfunction, QT prolongation, central nervous system toxicity, and gut microbiota disruption [20,21,22,23]. Unlike 3GCs and PEN-BLis, fluoroquinolones may exert distinct effects on the intestinal microbiome owing to their different mechanisms of action, which could also influence clinical outcomes [24]. These mechanisms may exacerbate COVID-19 pathophysiology by increasing susceptibility to arrhythmia, altering neuroinflammatory responses, or dysregulating immune homeostasis [25]. In hospitalized patients with COVID-19, empiric antibiotics are frequently administered owing to concern for bacterial coinfection and limited early antiviral options. In patients with moderate to severe illness, compromised pulmonary function can impose cardiopulmonary stress, with elevated creatine kinase and troponin indicating myocardial strain. Within this clinical milieu, the proarrhythmic and autonomic effects characteristic of fluoroquinolones could potentially exacerbate hemodynamic stability, while higher vasopressor use may reflect subclinical circulatory instability or autonomic imbalance consistent with pharmacovigilance signals [26,27]. The absence of antiviral or immunomodulatory activity further limits the biological rationale for fluoroquinolones in viral respiratory infections, including COVID-19 [28]. Collectively, these mechanistic considerations provide a biologically plausible framework for understanding that fluoroquinolone use, compared with 3GCs, is associated with higher risks of mechanical ventilation, ICU admission, vasopressor use, in-hospital mortality, and the composite endpoint. Accordingly, preventive monitoring and risk mitigation strategies are warranted when fluoroquinolones are indicated.

For PEN–BLis, although the association was not statistically significant, likely owing to smaller sample sizes and lower event rates, the consistent direction of point estimates across outcomes suggests a potential safety signal that warrants further investigation in larger cohorts. Similar findings were reported in a previous study of community-acquired pneumonia, revealing higher in-hospital mortality and *Clostridioides difficile* infection with ampicillin–sulbactam than with ceftriaxone [29], consistent with our findings for PEN–BLis versus 3GCs in patients with COVID-19. PEN–BLis possesses broad-spectrum activity, particularly against β-lactamase–producing organisms, and is often prescribed based on institutional practices or perceived infection severity. However, without bacterial confirmation, their empirical use may not yield clinical benefits, potentially contributing to antibiotic-related harm.

We conducted sensitivity analyses to assess the robustness of our findings to residual confounding, infection misclassification, temporal shifts in care, agent-level heterogeneity, and treatment modifications. The findings revealed the following: First, re-estimating the PS after incorporating additional baseline physiologic markers, including vital signs, laboratory parameters, and oxygenation (SpO_2_/SaO_2_), did not alter the direction or estimate interpretation. This suggests that the observed associations are not fully attributable to baseline differences in illness severity. However, direct measures of oxygen requirements and radiographic burden were not available in the OMOP-CDM, and the timing of symptom onset was likewise unavailable; therefore, these variables could not be incorporated. Future studies should integrate OMOP-CDM cohorts with hospital electronic medical records to enable analyses that incorporate oxygen requirements, structured radiographic indicators, and the timing of symptom onset. Second, the consistency of sensitivity analysis results, excluding patients with early positive cultures or markedly elevated baseline procalcitonin levels, supports the robustness of primary and secondary outcomes by addressing potential bias arising from early diagnostic information that may influence antibiotic selection. Nevertheless, these findings should be interpreted cautiously, given possible residual confounding from unmeasured severity factors and the inherent challenges of fully excluding bacterial coinfections. Third, analyses restricted to the Omicron period were consistent with the primary analysis, indicating that the elevated risks associated with fluoroquinolone use persisted despite changes in epidemiology and therapeutic practices. For PEN–BLis versus 3GCs, estimates were generally aligned with the primary analysis; however, the association with vasopressor use shifted toward a reduction during the Omicron period. Because the Omicron analyses drew on fewer hospitals, resulting in smaller sample sizes, the attenuation is most plausibly a sample-size-related fluctuation rather than a true period-specific difference. Accordingly, this vasopressor use should be interpreted with caution. Fourth, agent-level comparison limited to commonly used regimens (ceftriaxone vs. levofloxacin or moxifloxacin) reproduced the fluoroquinolone-associated risks, underscoring their relevance to routine management of lower respiratory tract infection. Finally, in an as-treated analysis involving censoring at initiation of any non-index antimicrobial, estimates were directionally consistent with the intention-to-treat approach, with in-hospital mortality remaining significantly elevated, albeit attenuated, though informative censoring may still occur when treatment changes are prognosis-driven.

These findings have key implications for antimicrobial stewardship and pandemic response strategies. In South Korea, hospitalization for COVID-19 was generally reserved for patients with moderate to severe illness, while those with mild disease were managed in community-based treatment centers or under home-isolation [30]. Therefore, the hospitalized cohort in this study likely represents patients with at least moderate illness, and antibiotic therapy was primarily initiated empirically on admission. As new viral respiratory pathogens emerge, empirical antibiotic use will likely remain common in the early phases of diagnostic uncertainty. These context-specific factors—clinician familiarity with guidelines, perceived severity at admission, antimicrobial-stewardship activities, formulary availability or restrictions, and the limited availability of early antivirals—likely influenced empirical antibiotic class selection at admission, underscoring the need for robust adjustment for confounding by indication in our analyses [31]. However, fluoroquinolones may still be appropriate in certain cases, including patients with severe respiratory symptoms or those transferred from secondary or long-term care facilities where respiratory multidrug-resistant pathogens are a concern. Therefore, when fluoroquinolone use is warranted in hospitalized patients with COVID-19, close monitoring and timely reassessment based on culture and susceptibility results are essential to mitigate the risk of progression to critical conditions, such as mechanical ventilation, ICU transfer, or vasopressor use. Although our study could not fully adjust for baseline disease severity or prior care settings—an acknowledged limitation in our study—our robust covariate adjustment and matching suggest that the observed associations reflect the pharmacologic effects of fluoroquinolones rather than confounding from disease severity.

This study has several notable strengths. First, to our knowledge, this is the first large-scale, real-world study to directly compare the clinical outcomes of multiple empirical antibiotic classes in hospitalized patients with COVID-19 using standardized data from a nationwide multicenter network. Second, the head-to-head comparative design enables direct inferences on relative risks, rather than relying on descriptive analyses of prescribing trends. These strengths are supported by access to a large, standardized dataset from the OMOP-CDM network, which facilitated comprehensive cross-institutional comparisons of antibiotic treatment patterns and outcomes. Third, rigorous confounding control was incorporated through sensitivity analyses addressing residual confounding, infection misclassification, temporal shifts in care, agent-level heterogeneity, and treatment variations. This methodological rigor helped mitigate inherent limitations, including short antibiotic exposure, the acute and dynamic nature of COVID-19, and the potential for treatment switching, discontinuation, or combination therapy, factors that often impede causal inference in similar studies. Finally, the study focused on clinically meaningful, high-acuity endpoints (mechanical ventilation, ICU admission, vasopressor use, and in-hospital mortality). These outcomes are objectively defined and carry clinically relevant implications for patient prognosis and hospital resource utilization and planning during pandemics.

However, some limitations must be acknowledged. Concomitant or early sequential antibiotic use was not included as a baseline matching covariate, potentially introducing residual confounding. Since this exposure varies over time, including it in the baseline PS could misclassify treatment and introduce immortal-time or time-dependent bias, and the OMOP-CDM cannot reliably distinguish switching from add-on therapy. To mitigate these challenges, sensitivity analyses were conducted, including an as-treated approach censoring at initiation of any non-index antimicrobial and a per-protocol analysis. Nevertheless, some residual confounding from early co-therapy may persist. Therefore, future studies should use precise date–time data on medication orders and administrations to distinguish add-on from switching and incorporate these time-updated exposures to improve causal inference. Since add-on or switching often occurs in more severe cases, any residual co-therapy could bias between-group contrasts, potentially away from the null if concentrated among patients in severe conditions, so effect sizes should be interpreted cautiously. Although some covariates demonstrated standardized mean differences > 0.1 after matching, the procedure significantly reduced major pre-matching imbalances (Supplementary Figures S1A,B–S9A,B, S10A, S11A and S12A,B). Qualitative variations in effect estimates were observed across hospitals, likely reflecting differences in size, resistance patterns, and treatment protocols. However, to address this heterogeneity, data from several hospitals were included and meta-analyzed using hospital-specific estimates pooled across 12 tertiary or regional hub centers. Empirical antibiotic class distributions were broadly similar across hospitals, reducing concern that practice differences alone influenced the pooled effect. At 80% power, the comparisons for PEN–BLis were limited, with minimum detectable HRs ranging from approximately 1.75 to 2.44, indicating that null findings cannot exclude smaller, clinically relevant effects. Bacterial coinfections at admission may have been present among some included patients. To reduce baseline misclassification and capture patients hospitalized primarily for COVID-19, individuals with systemic antibiotic use, bacterial infection, or respiratory disorders within 30 days before cohort entry were excluded, and standardized coinfection screens were applied using positive bacterial cultures within 48–72 h of admission and biomarker thresholds, including procalcitonin ≥ 2 ng/mL. Despite these measures, ascertainment may remain incomplete owing to variation in testing intensity and data availability across sites, allowing for potential residual misclassification; however, sensitivity analyses with these stricter exclusions yielded effect estimates consistent in direction with the primary results. Although we attempted to include macrolides in the analysis, the small number of hospitalized patients receiving this class precluded meaningful evaluation. This finding aligns with nationwide claims data indicating that fluoroquinolones, 3GCs, and PEN-BLis are the most commonly prescribed antibiotics among hospitalized patients with COVID-19 in Korea [32]. The nationwide scope of this dataset ensures that our comparison of the two most used classes reflects the dominant prescribing pattern across the country. The lack of detailed dose and duration information limits the evaluation of dose response, warranting cautious interpretation of effect sizes. While our analysis focused on 30-day short-term outcomes, longer-term consequences such as resistance development and delayed complications warrant further investigation.

Despite these limitations, this study provides valuable insights to guide antibiotic stewardship during respiratory viral pandemics. As empirical antibiotic use remains common under diagnostic uncertainty, our findings underscore the need for risk profiling based on antibiotic class in initial prescribing decisions. Additionally, close monitoring and prompt discontinuation should be considered, particularly given their potential adverse effects, further guided by evidence of bacterial presence. When atypical bacterial infection is strongly suspected, fluoroquinolones may be used, although macrolides are generally preferred as the initial option. Future studies should integrate clinical severity measures, microbiological confirmation, and longitudinal outcomes to refine risk stratification and promote more targeted, evidence-based antibiotic use.

## 4. Materials and Methods

### 4.1. Data Sources

This retrospective observational cohort study utilized electronic health record data of approximately 15 million patients across 12 tertiary hospitals in South Korea. This institutions include Ajou University Medical Center (AUMC), Ewha Womans University Medical Center (EUMC), Gyeongsang National University Hospital (GNUH), International St. Mary’s Hospital (ISH), Jecheon Myongji Hospital (JCMJ), Kangdong Sacred Heart Hospital (KDH), Kyunghee University Medical Center (KHMC), Gangwon National University Medical Center (KWMC), Myongji Hospital (MJH), Soonchunhyang University Hospital Bucheon Center (SCHBC), Soonchunhyang University Hospital Cheonan Center (SCHCA), and Wonkwang University Hospital (WKUH). The databases were de-identified and standardized using the OMOP-CDM (version 5.3.1) [11,12]. This framework was developed by the Observational Health Data Sciences and Informatics (OHDSI) Network. The OMOP-CDM enables the harmonization of heterogeneous data sources, enhancing reproducibility and generalizability of real-world evidence across diverse healthcare settings, thereby supporting large-scale observational studies in a standardized format [11,12]. To address potential inter-hospital heterogeneity and maximize the generalizability of our results, we used data from all available university-affiliated institutions that had adopted the OMOP-CDM, ensuring broad regional coverage. The study was approved by the Institutional Review Board (IRB) of EUMC (IRB No. EUMC 2022-07-045; approval date: 1 August 2022), with informed consent waived due to the use of de-identified data.

### 4.2. Study Design and Participants

**Figure 7:**
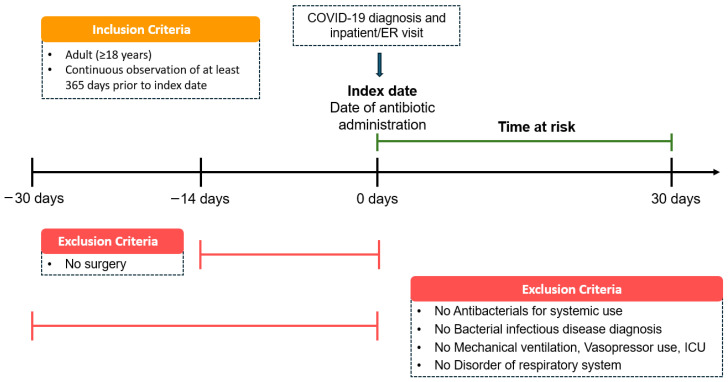
Cohort construction and patient distribution after propensity score matching for antibiotic comparisons. 3GCs: Third-generation cephalosporins; AUMC: Ajou University Medical Center; ER: emergency room; EUMC: Ewha Womans University Medical Center; GNUH: Gyeongsang National University Hospital; ICU: intensive care unit; ISH: International St. Mary’s Hospital; JCMJ: Jecheon Myongji Hospital; KDH: Kangdong Sacred Heart Hospital; KHMC: Kyunghee University Medical Center; KWMC: Kangwon National University Medical Center; MJH: Myongji Hospital; PEN–BLis: aminopenicillin/β-lactamase inhibitor combinations; SCHCA: Soonchunhyang University Hospital Cheonan Center; SCHBC: Soonchunhyang University Hospital Bucheon Center; WKUH: Wonkwang University Hospital.

This multicenter, retrospective cohort study included adult patients (≥18 years) hospitalized with COVID-19 from 1 January 2020 to 31 May 2025. Eligible patients had a confirmed COVID-19 diagnosis and received the systemic antibiotics of interest on the same day as hospital admission or emergency department presentation. The date of concurrent COVID-19 diagnosis, hospitalization, and antibiotic therapy was defined as the index date (Figure 7). The time-at-risk was defined as a fixed 30-day period from the index admission, without censoring at discharge. Study entry was restricted to the initial hospitalization, and outcomes were evaluated solely within this pre-specified risk window, regardless of subsequent readmissions. To ensure sufficient baseline characterization, patients were required to have at least 365 consecutive days of observation before the index date.

To mitigate confounding from prior infections, prophylactic antibiotic use, or advanced illness, individuals were excluded if they had received systemic antibiotics, been diagnosed with a bacterial infection or respiratory disorder, required mechanical ventilation or vasopressors, or had ICU admission within 30 days before the index date. Patients who underwent surgery within 14 days before the index date were also excluded.

Antibiotic exposure was classified into three groups: 3GCs, fluoroquinolones, and PEN–BLis. Ceftazidime and piperacillin-tazobactam were excluded from the 3GC and the PEN-BLi groups, respectively, due to their distinct spectrum and clinical usage. Supplementary Tables S23–S25 provides a list of agents within each class. The 3GCs served as the reference group for comparison with the other two antibiotic classes in all analyses.

### 4.3. Outcomes

The primary outcomes were all-cause in-hospital mortality and a composite outcome, defined as the occurrence of mechanical ventilation, ICU admission, or vasopressor use within the time-at-risk period. The secondary outcomes included initiation of mechanical ventilation, ICU admission, and vasopressor use. To evaluate the effect of the initial empirical antibiotic class on clinical outcomes, we established treatment cohorts across the 12 hospitals and conducted a comparative analysis of fluoroquinolones and PEN–BLis relative to 3GCs. To control potential confounding, PS adjustment methods were applied. The time-at-risk period commenced on the index date, extending for 30 days. All outcomes were assessed within this predefined interval to evaluate acute clinical deterioration from COVID-19 and its association with initial antibiotic exposure.

### 4.4. Statistical Analysis

To address potential confounding, large-scale PS adjustment was performed using regularized logistic regression models incorporating a comprehensive set of baseline covariates. These covariates included demographic factors (sex, age group, and index year), the Charlson Comorbidity Index, and clinical characteristics measured on or within 30 days before the index date. Clinical conditions included acidosis and organ failure—specifically, respiratory, cardiovascular, renal, and hepatic—all identified using ICD-10 codes. Medication and procedural variables included systemic corticosteroid use and diagnostic testing for procalcitonin, blood cultures, and lactate, each recorded on or within 30 days before the index date. All covariates, except laboratory values, were binary, and missing binary variables were recorded as absent. No imputation was applied to laboratory values. Missing data were indirectly addressed through PS matching, balancing covariates across antibiotic exposure groups.

PSs were estimated separately for each pairwise comparison between the target antibiotic groups (fluoroquinolones or PEN–BLis) and the comparator group (3GCs). To minimize poor matches and residual confounding, 1:N matching was conducted using a caliper width of 0.2 on the standardized logit PS, with nearest-neighbor, without-replacement matching, and no upper limit on the comparator-to-target ratio. Patients in either group without an eligible in-caliper match were excluded. Covariate balance was assessed using standardized mean differences and visualized with love plots. Analyses were performed using the OMOP-CDM (version 5.3.1) through the ATLAS interactive platform (version 2.12.0) and R (version 4.1.0).

Cox proportional hazards models were used to estimate HRs and 95% CIs for each clinical outcome. Time-to-event analyses were performed using Kaplan–Meier curves. Site-specific HRs were first estimated at each participating hospital, and the results were subsequently combined through meta-analysis, with statistical heterogeneity quantified using the I^2^ statistic. Minimum detectable HRs for the primary analysis outcomes were estimated using standard Cox model calculations, with 80% power. To detect systematic bias or unmeasured confounding, 119 negative control outcomes were selected (Supplementary Table S26) following a process similar to that outlined by Voss et al. [33].

To evaluate the robustness of our effect estimates to potential unmeasured confounding, a quantitative bias analysis was performed by calculating E-values. The E-value quantifies the minimal strength of association, on the risk-ratio scale, that an unmeasured confounder would need with the exposure and outcome, beyond the measured covariates, to fully explain the observed association [34]. E-values were calculated for HR point estimates and the lower bounds of their 95% CIs using the standard formula. All analyses were conducted in R (version 4.1.0).

### 4.5. Sensitivity Analyses

To evaluate the robustness of the primary effect estimates to residual confounding and analytic decisions, sensitivity analyses were conducted using the same outcome definitions. First, to account for baseline illness severity, we re-estimated the PS after expanding covariates to include vital signs (heart rate, systolic/diastolic blood pressure, respiratory rate, body temperature), laboratory parameters (white blood cell count, C-reactive protein), and oxygenation (SpO_2_/SaO_2_). This analysis addresses residual confounding based on severity but remains susceptible to measurement error, missing data, and limited covariate overlap. Second, to minimize bias from unrecognized bacterial coinfection or acute bacterial inflammation, we excluded patients with any of the following: (i) positive microbiological cultures within 48–72 h of admission; (ii) baseline procalcitonin ≥ 2 ng/mL. This approach still misclassifies infection when testing is incomplete, reducing precision and generalizability. Third, the cohort was restricted to admissions during the Omicron period to assess temporal transportability. This restriction introduced era-specific practice patterns and reduced power. Fourth, to examine within-class heterogeneity, we performed agent-level comparisons restricted to the most commonly prescribed regimens (ceftriaxone vs. levofloxacin or moxifloxacin). These comparisons may be more vulnerable to indication channeling at the molecular level and smaller cell sizes. Finally, we estimated an as-treated effect by censoring follow-up at the initiation of any non-index antimicrobial, reducing susceptibility to immortal-time and time-dependent confounding inherent in intention-to-treat-like specifications. With respect to early co-therapy, concomitant or sequential antibiotic use changes over time. In our multi-site OMOP CDM, medication records are captured mostly at the date level rather than with time-of-day stamps, which prevents establishing the within-day sequence needed to distinguish add-ons from switching after admission. Therefore, we did not implement time-varying adjustments or within-hospital-day matching. Because time of day information was incomplete and would misclassify exposure timing, and because conditioning on post-index treatment changes would no longer estimate the effect of the initial antibiotic class selected at admission.

## 5. Conclusions

In this large, multicenter cohort of hospitalized patients with COVID-19, initial empirical fluoroquinolone use was associated with significantly increased rates of critical short-term outcomes compared with 3GCs, even after extensive confounding adjustment. While fluoroquinolones remain valuable in certain high-risk cases, these findings underscore the importance of judicious empirical antibiotic selection and prompt reassessment based on clinical and microbiological evidence. Collectively, these findings offer clear guidance for stewardship teams to limit unnecessary broad-spectrum use, promote guideline-based prescribing, and reinforce systematic reassessment and monitoring during respiratory viral surges, especially in settings with high empirical use despite low bacterial coinfection rates.

## Data Availability

The data are not publicly available due to privacy or ethical restrictions.

## Supplementary Materials

The following supporting information can be downloaded at: https://www.mdpi.com/article/10.3390/ph18101588/s1, Tables S1A–S12A: Baseline characteristics before and after propensity score adjustment: fluoroquinolones vs. third-generation cephalosporins across 12 hospitals (AUMC, EUMC, GNUH, ISH, JCMJ, KDH, KHMC, KWMC, MJH, SCHBC, SCHCA, and WKUH); Tables S1B–S9B and S12B: Baseline characteristics before and after propensity score adjustment: aminopenicillin/β-lactamase inhibitor combinations vs. third-generation cephalosporins across 10 hospitals (AUMC, EUMC, GNUH, ISH, JCMJ, KDH, KHMC, KWMC, MJH, and WKUH. Note: Tables S10B and S11B are not included due to the small sample sizes from SCHBC and SCHCA); Tables S13A–S22A: Baseline characteristics before and after propensity-score adjustment with expanded severity covariates: fluoroquinolones vs. third-generation cephalosporins across 10 hospitals (GNUH, ISH, JCMJ, KDH, KHMC, KWMC, MJH, SCHBC, SCHCA, and WKUH); Tables S13B–S19B and S22B: Baseline characteristics before and after propensity-score adjustment with expanded severity covariates: aminopenicillin/β-lactamase inhibitor combinations vs. third-generation cephalosporins across 8 hospitals (GNUH, ISH, JCMJ, KDH, KHMC, KWMC, MJH, and WKUH. Note: Tables S20B and S21B are not included due to the small sample sizes from SCHBC and SCHCA); Table S23: List of third-generation cephalosporin drug concepts included in the analysis; Table S24: List of fluoroquinolones drug concepts included in the analysis; Table S25: List of aminopenicillin/β-lactamase inhibitor combinations drug concepts included in the analysis; Table S26: Negative control outcomes selected for the study; Figures S1A–S12A: Love plots of standardized mean differences before and after propensity score adjustment: FQs vs. 3GCs across 12 hospitals (AUMC, EUMC, GNUH, ISH, JCMJ, KDH, KHMC, KWMC, MJH, SCHBC, SCHCA, and WKUH); Figures S1B–S9B and S12B: Love plots of standardized mean differences before and after propensity score adjustment: PEN-BLis vs. 3GCs across 10 hospitals (AUMC, EUMC, GNUH, ISH, JCMJ, KDH, KHMC, KWMC, MJH, and WKUH. Note: Figures S10B and S11B are not included due to the small sample sizes from SCHBC and SCHCA); Figure S13A,B: Kaplan–Meier analysis of primary outcomes after propensity score matching: 3GCs vs. FQs; Figure S14A,B: Kaplan–Meier analysis of primary outcomes after propensity score matching: 3GCs vs. PEN-BLis; Figure S15A–C: Kaplan–Meier analysis of secondary outcomes after propensity score matching: 3GCs vs. FQs; Figure S16A,B: Kaplan–Meier analysis of secondary outcomes after propensity score matching: 3GCs vs. PEN-BLis; Figures S17A,B–S20A,B: Comparative risks of primary and secondary outcomes after excluding patients with evidence of bacterial co-infection (early positive cultures or elevated procalcitonin); Figures S21A,B and S22A,B: Comparative risks of primary and secondary outcomes during the Omicron period; Figures S23 and S24: Comparative risks of primary and secondary outcomes between ceftriaxone and levofloxacin/moxifloxacin; Figures S25A,B and S26A,B: Comparative risks of primary and secondary outcomes: as-treated analysis.

## Abbreviations

The following abbreviations are used in this manuscript:

3GCs	Third-Generation Cephalosporins
AUMC	Ajou University Medical Center
CI	Confidence Interval
EUMC	Ewha Womans University Medical Center
FQs	Fluoroquinolones
GNUH	Gyeongsang National University Hospital
HR	Hazard Ratio
ICU	Intensive Care Unit
IRB	Institutional Review Board
ISH	International St. Mary’s Hospital
JCMJ	Jecheon Myongji Hospital
KDH	Kangdong Sacred Heart Hospital
KHMC	Kyunghee University Medical Center
KWMC	Kangwon National University Medical Center
MJH	Myongji Hospital
OMOP-CDM	Observational Medical Outcomes Partnership Common Data Model
OHDSI	Observational Health Data Sciences and Informatics
PEN–BLis	Aminopenicillins with β-lactamase inhibitors
PS	Propensity Score
SCHBC	Soonchunhyang University Hospital Bucheon Center
SCHCA	Soonchunhyang University Hospital Cheonan Center
WKUH	Wonkwang University Hospital
