# Supplementary material for: Comparative Safety of Empirical Antibiotic Classes in Newly Hospitalized COVID-19 Patients
Source: Pharmaceuticals (Basel). 2025 Oct 21;18(10):1588. doi: 10.3390/ph18101588 (PMC12566716; doi:10.3390/ph18101588)
Supplement: Supplementary file 1 [file pharmaceuticals-18-01588-s001.zip › pharmaceuticals-3837189-supplementary.pdf]

Supplementary Table S1A. Baseline characteristics before and after propensity score adjustment: fluoroquinolones vs third-generation cephalosporins (AUMC)

Characteristic	Before PS adjustment			After PS adjustment		
	Target (%)	Comparator (%)	Std. Diff	Target (%)	Comparator (%)	Std. Diff
**Age group**						
20-24	-7.2%	-1.2%	0.05	-7.7%	-1.0%	0.08
35-39	-7.2%	-1.2%	0.07	-7.7%	-1.0%	0.07
40-44	-7.2%	3.1%	0.07	-7.7%	1.6%	0.17
45-49	-7.2%	3.3%	-0.02	-7.7%	2.5%	0.03
50-54	-7.2%	7.3%	-0.29	-7.7%	4.5%	-0.18
55-59	-7.2%	5.7%	-0.14	-7.7%	6.3%	-0.15
60-64	-7.2%	9.7%	-0.28	-7.7%	5.7%	-0.13
65-69	17.4%	10.4%	0.20	15.4%	13.2%	0.06
70-74	7.2%	10.4%	-0.11	7.7%	10.9%	-0.11
75-79	21.7%	12.6%	0.25	21.5%	21.7%	0.00
80-84	14.5%	15.2%	-0.02	15.4%	10.7%	0.14
85-89	15.9%	10.4%	0.16	15.4%	12.5%	0.09
90-94	-7.2%	6.4%	-0.03	-7.7%	6.7%	-0.02
**Index Year**						
2020	-7.2%	-1.2%	0.10	-7.7%	-1.0%	0.06
2021	-7.2%	9.5%	-0.20	-7.7%	8.9%	-0.17
2022	43.5%	41.9%	0.03	46.2%	43.0%	0.06
2023	24.6%	33.2%	-0.19	26.2%	25.8%	0.01
2024	24.6%	14.5%	0.26	20.0%	20.7%	-0.02
2025	-7.2%	-1.2%	0.10	-7.7%	-1.0%	0.09
**Comorbidities**						
Acute hypoxemic respiratory failure	10.1%	2.8%	0.30	10.8%	9.4%	0.05
Acute kidney injury	-7.2%	9.0%	-0.19	-7.7%	8.1%	-0.14
Acute pulmonary edema	-7.2%	-1.2%	0.10	-7.7%	-1.0%	0.13
Acute respiratory distress syndrome	-7.2%	-1.2%	0.27	-7.7%	2.6%	0.17
Acute respiratory failure	-7.2%	-1.2%	0.22	-7.7%	-1.0%	0.23
Agranulocytosis	-7.2%	2.8%	-0.10	-7.7%	4.6%	-0.18
Aplastic anemia	-7.2%	-1.2%	0.14	-7.7%	1.8%	-0.02
Ascites	-7.2%	-1.2%	0.07	-7.7%	-1.0%	0.13
Bronchopneumonia	-7.2%	-1.2%	0.02	-7.7%	2.1%	-0.05
Chronic obstructive lung disease	7.2%	1.7%	0.27	7.7%	4.5%	0.13
Congestive heart failure	-7.2%	-1.2%	0.05	-7.7%	1.1%	0.04
End stage renal disease on dialysis due to type 2 diabetes mellitus	-7.2%	4.0%	-0.06	-7.7%	3.9%	-0.05
End-stage renal disease	-7.2%	1.4%	0.00	-7.7%	4.1%	-0.15
Heart failure	-7.2%	5.9%	-0.07	-7.7%	6.5%	-0.08
Hepatorenal syndrome	-7.2%	-1.2%	0.13	-7.7%	-1.0%	0.15
Idiopathic pulmonary fibrosis	-7.2%	-1.2%	0.26	-7.7%	-1.0%	0.25
Ketoacidotic coma due to type 2 diabetes mellitus	-7.2%	-1.2%	0.13	-7.7%	-1.0%	0.14
Left heart failure	-7.2%	-1.2%	0.10	-7.7%	-1.0%	0.10
Myelodysplastic syndrome, NOS, of bone marrow	-7.2%	-1.2%	0.17	-7.7%	1.0%	0.15
Pneumonia	55.1%	29.1%	0.54	52.3%	59.9%	-0.15
Pneumonia due to Pseudomonas	-7.2%	-1.2%	0.13	-7.7%	-1.0%	0.10
Pulmonary embolism	-7.2%	-1.2%	0.14	-7.7%	-1.0%	0.16
Pulmonary emphysema	-7.2%	-1.2%	0.19	-7.7%	-1.0%	0.22
Renal failure syndrome	-7.2%	3.6%	0.11	-7.7%	3.6%	-0.03
Septic shock	11.6%	3.3%	0.32	10.8%	9.5%	0.04
Shock	-7.2%	-1.2%	0.13	-7.7%	-1.0%	0.10
Thrombophlebitis of deep veins of lower extremity	-7.2%	1.4%	0.00	-7.7%	2.5%	-0.07
Traumatic shock	-7.2%	-1.2%	0.07	-7.7%	-1.0%	0.09
Viral pneumonia	27.5%	18.0%	0.23	27.7%	27.8%	0.00
**Medications**						
Dexamethasone	31.9%	24.9%	0.16	33.8%	29.0%	0.11
Hydrocortisone	-7.2%	-1.2%	0.13	-7.7%	-1.0%	0.16
Methylprednisolone	8.7%	1.4%	0.34	-7.7%	4.1%	0.03
Prednisolone	-7.2%	2.8%	-0.10	-7.7%	3.6%	-0.13

AUMC, Ajou University Medical Center; PS, Propensity Score; Std. Diff, Standardized Difference.

Supplementary Table S1B. Baseline characteristics before and after propensity score adjustment: aminopenicillin/β-lactamase inhibitor combinations vs third-generation cephalosporins (AUMC)

Characteristic	Before PS adjustment			After PS adjustment		
	Target (%)	Comparator (%)	Std. Diff	Target (%)	Comparator (%)	Std. Diff
**Age group**						
30-34	-55.6%	-1.2%	0.45	-55.6%	-1.2%	0.45
45-49	-55.6%	3.3%	0.59	-55.6%	3.3%	0.59
50-54	-55.6%	7.3%	0.13	-55.6%	7.4%	0.13
60-64	-55.6%	9.7%	0.35	-55.6%	9.7%	0.35
70-74	-55.6%	10.4%	0.02	-55.6%	10.4%	0.02
**Index Year**						
2021	-55.6%	9.5%	0.35	-55.6%	9.5%	0.35
2022	-55.6%	41.9%	0.05	-55.6%	42.0%	0.05
2023	-55.6%	33.2%	-0.25	-55.6%	33.0%	-0.24
2024	-55.6%	14.5%	-0.10	-55.6%	14.5%	-0.10
**Comorbidities**						
Acute kidney injury	-55.6%	9.0%	0.07	-55.6%	9.0%	0.07
Acute respiratory distress syndrome	-55.6%	-1.2%	0.44	-55.6%	-1.2%	0.44
Acute respiratory failure	-55.6%	-1.2%	0.48	-55.6%	-1.2%	0.48
Bronchopneumonia	-55.6%	-1.2%	0.42	-55.6%	-1.2%	0.42
End stage renal disease on dialysis due to type 2 diabetes mellitus	-55.6%	4.0%	0.56	-55.6%	4.0%	0.56
End-stage renal disease	-55.6%	1.4%	0.41	-55.6%	1.4%	0.41
Hypertensive renal failure	-55.6%	-1.2%	0.42	-55.6%	-1.2%	0.42
Pneumonia	-55.6%	29.1%	0.09	-55.6%	29.2%	0.09
Pneumonia and influenza	-55.6%	-1.2%	0.47	-55.6%	-1.2%	0.47
Pulmonary edema	-55.6%	-1.2%	0.45	-55.6%	-1.2%	0.45
Septic shock	-55.6%	3.3%	0.31	-55.6%	3.3%	0.30
**Medications**						
Dexamethasone	-55.6%	24.9%	-0.06	-55.6%	25.0%	-0.06
Methylprednisolone	-55.6%	-1.2%	0.47	-55.6%	-1.2%	0.47

AUMC, Ajou University Medical Center; PS, Propensity Score; Std. Diff, Standardized Difference.

Supplementary Table S2A. Baseline characteristics before and after propensity score adjustment: fluoroquinolones vs third-generation cephalosporins (EUMC)

Characteristic	Before PS adjustment			After PS adjustment		
	Target (%)	Comparator (%)	Std. Diff	Target (%)	Comparator (%)	Std. Diff
**Age group**						
00-04	-2.0%	-0.8%	-0.03	-2.5%	0.9%	-0.05
15-19	-2.0%	1.0%	-0.07	-2.5%	1.0%	-0.06
20-24	-2.0%	1.1%	0.01	-2.5%	-0.8%	0.07
25-29	-2.0%	1.6%	-0.08	-2.5%	1.0%	0.00
30-34	2.0%	1.5%	0.04	2.5%	0.9%	0.13
35-39	2.4%	2.9%	-0.03	2.5%	2.6%	-0.01
40-44	-2.0%	2.6%	-0.07	-2.5%	2.4%	-0.03
45-49	-2.0%	2.0%	-0.06	-2.5%	1.3%	0.01
50-54	4.0%	3.4%	0.03	3.4%	2.4%	0.06
55-59	-2.0%	3.4%	-0.15	-2.5%	2.5%	-0.12
60-64	6.4%	7.5%	-0.04	5.9%	7.1%	-0.05
65-69	8.0%	8.8%	-0.03	7.9%	7.1%	0.03
70-74	10.0%	8.0%	0.07	8.9%	8.3%	0.02
75-79	17.7%	13.5%	0.12	17.2%	16.7%	0.01
80-84	19.3%	19.5%	-0.01	20.2%	20.4%	-0.01
85-89	16.1%	14.6%	0.04	16.7%	15.6%	0.03
90-94	5.6%	5.9%	-0.01	5.4%	6.0%	-0.02
95-99	-2.0%	2.1%	-0.04	-2.5%	2.9%	-0.10
**Index Year**						
2020	-2.0%	0.8%	0.039	-2.5%	1.1%	0.037
2021	2.8%	3.7%	-0.052	2.5%	3.4%	-0.057
2022	32.9%	39.3%	-0.134	34.5%	36.3%	-0.039
2023	38.6%	39.5%	-0.02	38.4%	36.7%	0.036
2024	22.9%	15.9%	0.177	21.7%	21.2%	0.012
2025	-2.0%	-0.8%	0.091	-2.5%	1.3%	0.013
**Comorbidities**						
Acidosis	2.0%	1.1%	0.07	-2.5%	1.1%	0.07
Acidosis due to type 2 diabetes mellitus	-2.0%	-0.8%	-0.01	-2.5%	-0.8%	0.05
Acute exacerbation of chronic obstructive pulmonary disease	6.8%	1.8%	0.25	4.4%	3.3%	0.06
Acute kidney injury	9.6%	10.4%	-0.03	10.8%	11.1%	-0.01
Acute respiratory distress syndrome	2.8%	-0.8%	0.18	-2.5%	-0.8%	0.11
Acute respiratory failure	-2.0%	-0.8%	0.06	-2.5%	-0.8%	0.05
Agranulocytosis	2.0%	0.8%	0.10	2.5%	-0.8%	0.16
Aplastic anemia	-2.0%	-0.8%	0.06	-2.5%	-0.8%	0.05
Ascites	-2.0%	-0.8%	0.08	-2.5%	-0.8%	0.11
Bacterial pneumonia	4.8%	1.8%	0.17	3.4%	3.7%	-0.01
Chronic obstructive pulmonary disease	8.4%	3.6%	0.21	5.4%	5.7%	-0.01
Chronic respiratory failure	2.4%	-0.8%	0.14	-2.5%	-0.8%	0.13
Congestive heart failure	2.4%	1.8%	0.04	-2.5%	2.7%	-0.05
End stage renal disease on dialysis due to type 2 diabetes mellitus	-2.0%	-0.8%	0.09	-2.5%	-0.8%	0.06
Febrile neutropenia	-2.0%	-0.8%	0.05	-2.5%	-0.8%	0.08
Heart failure	10.8%	7.2%	0.13	11.8%	9.9%	0.06
Hypovolemic shock	-2.0%	-0.8%	0.08	-2.5%	-0.8%	0.13
Left heart failure	-2.0%	-0.8%	0.06	-2.5%	-0.8%	0.05
Moderate chronic obstructive pulmonary disease	-2.0%	-0.8%	0.06	-2.5%	1.0%	-0.06
Plasma cell myeloma of bone marrow	-2.0%	-0.8%	0.09	-2.5%	-0.8%	0.06
Pneumonia	48.6%	16.9%	0.72	38.9%	42.1%	-0.07
Pneumonia caused by Human metapneumovirus	-2.0%	-0.8%	0.05	-2.5%	-0.8%	0.06
Pneumonia caused by Klebsiella pneumoniae	-2.0%	-0.8%	0.09	-2.5%	-0.8%	0.07
Pulmonary edema	-2.0%	1.5%	-0.02	-2.5%	3.1%	-0.15
Pulmonary embolism	4.8%	1.5%	0.19	4.4%	2.9%	0.08
Pulmonary emphysema	4.0%	1.5%	0.16	4.4%	2.6%	0.10
Renal failure syndrome	-2.0%	-0.8%	0.01	-2.5%	-0.8%	-0.01
Respiratory failure	2.4%	2.0%	0.03	3.0%	2.5%	0.03
Secondary thrombocytopenia	-2.0%	-0.8%	0.05	-2.5%	-0.8%	0.07
Septic shock	6.0%	4.4%	0.07	5.9%	5.6%	0.01
Thrombophlebitis of deep veins of lower extremity	2.0%	2.4%	-0.03	-2.5%	3.4%	-0.09
Viral pneumonia	-2.0%	-0.8%	0.11	-2.5%	-0.8%	0.03
White blood cell disorder	-2.0%	-0.8%	0.06	-2.5%	-0.8%	0.04
**Medications**						
Dexamethasone	-2.0%	-0.8%	0.04	-2.5%	-0.8%	0.08
Deflazacort	-2.0%	-0.8%	0.013	-2.5%	-0.8%	-0.013
Hydrocortisone	-2.0%	-0.8%	0.045	-2.5%	-0.8%	0.067
Methotrexate	-2.0%	-0.8%	0.013	-2.5%	-0.8%	-0.013
Methylprednisolone	16.9%	2.6%	0.496	7.4%	7.9%	-0.019
Prednisolone	2.4%	3.7%	-0.077	-2.5%	3.9%	-0.114

EUMC, Ewha Womans University Medical Center; PS, Propensity Score; Std. Diff, Standardized Difference.

Supplementary Table S2B. Baseline characteristics before and after propensity score adjustment: aminopenicillin/β-lactamase inhibitor combinations vs third-generation cephalosporins (EUMC)

Characteristic	Before PS adjustment			After PS adjustment		
	Target (%)	Comparator (%)	Std. Diff	Target (%)	Comparator (%)	Std. Diff
**Age group**						
15-19	5.4%	1.0%	0.25	-5.0%	5.0%	-0.05
25-29	10.8%	1.6%	0.39	11.0%	9.2%	0.06
30-34	8.1%	1.5%	0.32	9.0%	7.2%	0.06
35-39	-4.5%	2.9%	-0.01	-5.0%	2.9%	0.01
40-44	9.9%	2.6%	0.31	5.0%	8.5%	-0.14
45-49	7.2%	2.0%	0.25	6.0%	7.1%	-0.04
50-54	7.2%	3.4%	0.17	8.0%	9.6%	-0.06
55-59	-4.5%	3.4%	-0.04	-5.0%	1.7%	0.08
60-64	6.3%	7.5%	-0.05	7.0%	4.8%	0.09
65-69	10.8%	8.8%	0.07	12.0%	10.8%	0.04
70-74	4.5%	8.0%	-0.14	5.0%	4.4%	0.03
75-79	6.3%	13.5%	-0.24	7.0%	6.6%	0.02
80-84	6.3%	19.5%	-0.40	7.0%	10.0%	-0.11
85-89	-4.5%	14.6%	-0.43	-5.0%	2.3%	0.04
90-94	-4.5%	5.9%	-0.11	-5.0%	3.2%	0.04
**Index Year**						
2020	-4.5%	0.8%	0.01	-5.0%	2.1%	-0.09
2022	62.2%	39.3%	0.47	58.0%	58.4%	-0.01
2023	29.7%	39.5%	-0.21	33.0%	32.2%	0.02
2024	6.3%	15.9%	-0.31	7.0%	6.5%	0.02
2025	-4.5%	-0.8%	0.03	-5.0%	0.4%	0.07
**Comorbidities**						
Acute exacerbation of chronic obstructive pulmonary disease	-4.5%	1.8%	-0.08	-5.0%	0.7%	0.03
Acute kidney injury	-4.5%	10.4%	-0.42	-5.0%	1.5%	-0.05
Agranulocytosis	-4.5%	0.8%	0.01	-5.0%	0.6%	0.05
Aplastic anemia	-4.5%	-0.8%	0.07	-5.0%	0.5%	0.05
Bacterial pneumonia	-4.5%	1.8%	0.00	-5.0%	1.4%	0.05
Chronic obstructive pulmonary disease	-4.5%	3.6%	-0.18	-5.0%	1.0%	0.01
Chronic obstructive pulmonary disease with acute lower respiratory infection	-4.5%	-0.8%	0.10	-5.0%	-0.2%	0.14
Congestive heart failure	-4.5%	1.8%	-0.08	-5.0%	0.7%	0.03
Heart failure	-4.5%	7.2%	-0.21	-5.0%	1.1%	0.14
Pneumonia	7.2%	16.9%	-0.30	8.0%	8.3%	-0.01
Swelling of upper limb	-4.5%	-0.8%	0.10	-5.0%	-0.2%	0.13
Thrombophlebitis of deep veins of lower extremity	-4.5%	2.4%	-0.12	-5.0%	0.4%	0.07
White blood cell disorder	-4.5%	-0.8%	0.14	-5.0%	1.3%	0.05
**Medications**						
Dexamethasone	-4.5%	14.3%	-0.47	-5.0%	2.2%	-0.02
Deflazacort	-4.5%	-0.8%	0.07	-5.0%	-0.2%	0.11
Methotrexate	-4.5%	-0.8%	0.07	-5.0%	0.2%	0.10
Methylprednisolone	-4.5%	2.6%	-0.13	-5.0%	2.1%	-0.09

EUMC, Ewha Womans University Medical Center; PS, Propensity Score; Std. Diff, Standardized Difference.

Supplementary Table S3A. Baseline characteristics before and after propensity score adjustment: fluoroquinolones vs third-generation cephalosporins (GNUH)

Characteristic	Before PS adjustment			After PS adjustment		
	Target (%)	Comparator (%)	Std. Diff	Target (%)	Comparator (%)	Std. Diff
**Age group**						
40-44	-4.5%	-2.6%	-0.02	-5.7%	-2.8%	0.06
45-49	-4.5%	-2.6%	0.04	-5.7%	-2.8%	-0.01
50-54	-4.5%	4.2%	-0.14	-5.7%	3.4%	-0.07
55-59	-4.5%	3.1%	0.03	-5.7%	4.8%	-0.01
60-64	7.2%	7.8%	-0.02	9.2%	8.0%	0.04
65-69	7.2%	10.4%	-0.11	8.0%	11.3%	-0.11
75-79	12.6%	14.1%	-0.04	11.5%	13.6%	-0.06
80-84	20.7%	20.8%	0.00	16.1%	19.3%	-0.09
85-89	20.7%	16.1%	0.12	19.5%	18.6%	0.02
90-94	9.9%	6.2%	0.14	10.3%	5.2%	0.19
95-99	-4.5%	-2.6%	0.12	-5.7%	-2.8%	0.17
**Index Year**						
2021	-4.5%	-2.6%	0.04	-5.7%	-2.8%	0.11
2022	37.8%	50.5%	-0.26	37.9%	46.4%	-0.17
2023	50.5%	39.1%	0.23	48.3%	44.3%	0.08
2024	9.0%	7.8%	0.04	10.3%	7.3%	0.11
**Comorbidities**						
Acute exacerbation of chronic obstructive pulmonary disease	-4.5%	3.6%	-0.05	-5.7%	4.2%	-0.04
Acute kidney injury	8.1%	6.8%	0.05	8.0%	5.0%	0.12
Acute respiratory distress syndrome	-4.5%	-2.6%	0.22	-5.7%	-2.8%	0.21
Acute respiratory failure	4.5%	4.2%	0.02	-5.7%	4.8%	-0.14
Bacterial pneumonia	9.9%	-2.6%	0.37	5.7%	-2.8%	0.21
Chronic obstructive pulmonary disease	11.7%	9.4%	0.08	10.3%	8.8%	0.05
Congestive heart failure	-4.5%	2.6%	-0.06	-5.7%	-2.8%	-0.03
End-stage renal disease	4.5%	3.1%	0.07	-5.7%	-2.8%	0.10
Heart failure	-4.5%	4.2%	-0.03	-5.7%	4.4%	-0.12
Left heart failure	-4.5%	4.2%	-0.14	-5.7%	3.4%	-0.07
Localized edema	-4.5%	-2.6%	0.05	-5.7%	-2.8%	0.06
Pneumonia	36.0%	23.4%	0.28	27.6%	35.1%	-0.16
Pulmonary embolism	-4.5%	2.6%	0.01	-5.7%	-2.8%	-0.08
Pulmonary emphysema	-4.5%	-2.6%	-0.01	-5.7%	-2.8%	-0.03
Thrombophlebitis of deep veins of lower extremity	-4.5%	-2.6%	0.08	-5.7%	-2.8%	0.08
Viral pneumonia	22.5%	11.5%	0.30	14.9%	19.0%	-0.11
**Medications**						
Dexamethasone	26.1%	18.8%	0.18	23.0%	20.3%	0.07
fludrocortisone	-4.5%	-2.6%	-0.01	-5.7%	-2.8%	-0.09
Methylprednisolone	5.4%	-2.6%	0.21	-5.7%	-2.8%	0.15
Prednisolone	-4.5%	3.1%	-0.09	-5.7%	-2.8%	-0.01

GNUH, Gyeongsang National University Hospital; PS, Propensity Score; Std. Diff, Standardized Difference.

Supplementary Table S3B. Baseline characteristics before and after propensity score adjustment: aminopenicillin/β-lactamase inhibitor combinations vs third-generation cephalosporins (GNUH)

Characteristic	Before PS adjustment			After PS adjustment		
	Target (%)	Comparator	Std. Diff	Target (%)	Comparator	Std. Diff
**Age group**						
50-54	-83.3%	4.2%	0.42	-83.3%	4.2%	0.42
55-59	-83.3%	3.1%	0.47	-83.3%	3.2%	0.46
60-64	-83.3%	7.8%	0.27	-83.3%	7.9%	0.27
65-69	-83.3%	10.4%	0.18	-83.3%	10.5%	0.18
80-84	-83.3%	20.8%	-0.11	-83.3%	21.0%	-0.11
90-94	-83.3%	6.2%	0.33	-83.3%	6.3%	0.33
**Index Year**						
2022	-83.3%	50.5%	-0.35	-83.3%	50.8%	-0.36
2023	-83.3%	39.1%	0.58	-83.3%	38.7%	0.58
**Comorbidities**						
Pneumonia	-83.3%	23.4%	0.22	-83.3%	23.5%	0.22
Viral pneumonia	-83.3%	11.5%	0.15	-83.3%	11.5%	0.15

GNUH, Gyeongsang National University Hospital; PS, Propensity Score; Std. Diff, Standardized Difference.

Supplementary Table S4A. Baseline characteristics before and after propensity score adjustment: fluoroquinolones vs third-generation cephalosporins (ISH)

Characteristic	Before PS adjustment			After PS adjustment		
	Target (%)	Comparator (%)	Std. Diff	Target (%)	Comparator (%)	Std. Diff
**Age group**						
20-24	-8.9%	2.3%	0.23	-8.9%	3.8%	0.15
25-29	-8.9%	3.0%	0.03	-8.9%	4.7%	-0.06
30-34	-8.9%	2.1%	0.18	-8.9%	2.7%	0.13
45-49	-8.9%	3.0%	0.19	-8.9%	3.5%	0.16
50-54	-8.9%	4.8%	0.02	-8.9%	6.9%	-0.07
55-59	-8.9%	4.8%	-0.06	-8.9%	5.7%	-0.10
65-69	14.3%	6.4%	0.26	14.3%	8.3%	0.19
70-74	-8.9%	10.8%	-0.20	-8.9%	11.2%	-0.21
75-79	14.3%	12.0%	0.07	14.3%	11.0%	0.10
80-84	16.1%	13.8%	0.06	16.1%	12.1%	0.12
85-89	12.5%	16.8%	-0.12	12.5%	12.4%	0.00
90-94	-8.9%	6.7%	-0.06	-8.9%	4.3%	0.05
**Index Year**						
2020	14.3%	5.5%	0.30	14.3%	9.0%	0.17
2021	-8.9%	2.3%	-0.04	-8.9%	1.8%	0.00
2022	41.1%	31.0%	0.21	41.1%	35.6%	0.11
2023	23.2%	42.1%	-0.41	23.2%	39.5%	-0.36
2024	17.9%	17.5%	0.01	17.9%	12.9%	0.14
2025	-8.9%	1.6%	0.01	-8.9%	1.2%	0.05
**Comorbidities**						
Acute kidney injury	-8.9%	10.6%	-0.12	-8.9%	7.1%	0.00
Acute respiratory distress syndrome	-8.9%	1.8%	0.00	-8.9%	0.8%	0.09
Congestive heart failure	-8.9%	4.4%	-0.04	-8.9%	4.2%	-0.03
Febrile neutropenia	-8.9%	-1.1%	0.16	-8.9%	-0.8%	0.13
Heart failure	-8.9%	6.9%	-0.25	-8.9%	5.2%	-0.19
Pneumonia	28.6%	57.5%	-0.61	28.6%	27.8%	0.02
Pneumonia and influenza	-8.9%	2.3%	0.08	-8.9%	1.6%	0.13
Pulmonary embolism	-8.9%	-1.1%	0.10	-8.9%	0.9%	0.08
Respiratory failure	8.9%	15.4%	-0.20	8.9%	7.7%	0.04
Septic shock	-8.9%	2.3%	-0.04	-8.9%	1.2%	0.05
Shock	-8.9%	-1.1%	0.16	-8.9%	-0.8%	0.17
Staphylococcal pneumonia	-8.9%	-1.1%	0.16	-8.9%	-0.8%	0.17
Thrombophlebitis of deep veins of lower extremity	-8.9%	1.1%	0.05	-8.9%	-0.8%	0.11
**Medications**						
Dexamethasone	21.4%	47.6%	-0.57	21.4%	22.2%	-0.02
Methylprednisolone	-8.9%	-1.1%	0.08	-8.9%	-0.8%	0.12
Prednisolone	-8.9%	2.8%	0.05	-8.9%	2.5%	0.06

ISH, International St. Mary's Hospital; PS, Propensity Score; Std. Diff, Standardized Difference.

Supplementary Table S4B. Baseline characteristics before and after propensity score adjustment: aminopenicillin/β-lactamase inhibitor combinations vs third-generation cephalosporins (ISH)

Characteristic	Before PS adjustment			After PS adjustment		
	Target (%)	Comparator	Std. Diff	Target	Comparator	Std.
**Age group**						
45-49	-29.4%	3.0%	0.34	-29.4%	3.0%	0.34
50-54	-29.4%	4.8%	0.25	-29.4%	4.9%	0.25
55-59	-29.4%	4.8%	0.05	-29.4%	4.6%	0.06
60-64	-29.4%	6.7%	-0.03	-29.4%	6.7%	-0.03
65-69	-29.4%	6.4%	0.19	-29.4%	6.5%	0.18
70-74	-29.4%	10.8%	0.20	-29.4%	10.9%	0.19
75-79	-29.4%	12.0%	-0.01	-29.4%	11.6%	0.01
80-84	-29.4%	13.8%	-0.27	-29.4%	13.7%	-0.27
85-89	-29.4%	16.8%	0.02	-29.4%	17.0%	0.02
**Index Year**						
2022	58.8%	31.0%	0.58	58.8%	31.1%	0.58
2023	35.3%	42.1%	-0.14	35.3%	42.3%	-0.14
2024	-29.4%	17.5%	-0.37	-29.4%	17.3%	-0.36
**Comorbidities**						
Acute kidney injury	-29.4%	10.6%	0.04	-29.4%	10.2%	0.05
Acute respiratory distress syndrome	-29.4%	1.8%	0.40	-29.4%	1.6%	0.41
Aplastic anemia	-29.4%	-1.1%	0.29	-29.4%	-1.2%	0.29
Chronic obstructive lung disease	-29.4%	5.3%	0.40	-29.4%	5.4%	0.39
Congestive heart failure	-29.4%	4.4%	0.07	-29.4%	4.4%	0.07
Cor pulmonale	-29.4%	-1.1%	0.33	-29.4%	-1.2%	0.33
Heart failure	-29.4%	6.9%	-0.04	-29.4%	6.9%	-0.04
Left heart failure	-29.4%	2.5%	0.17	-29.4%	2.6%	0.17
Pneumococcal pneumonia	-29.4%	-1.1%	0.29	-29.4%	-1.2%	0.29
Pneumonia	76.5%	57.5%	0.41	76.5%	57.6%	0.41
Pneumonia due to Klebsiella pneumoniae	-29.4%	1.4%	0.24	-29.4%	1.4%	0.24
Pneumonia due to Pseudomonas	-29.4%	1.4%	0.24	-29.4%	1.4%	0.24
Pulmonary edema	-29.4%	2.3%	0.18	-29.4%	2.3%	0.18
Pulmonary embolism	-29.4%	-1.1%	0.47	-29.4%	-1.2%	0.47
Renal failure syndrome	-29.4%	4.6%	0.26	-29.4%	4.2%	0.28
Respiratory failure	41.2%	15.4%	0.60	41.2%	15.3%	0.60
Septic shock	-29.4%	2.3%	0.38	-29.4%	2.3%	0.38
**Medications**						
Dexamethasone	47.1%	47.6%	-0.01	47.1%	47.3%	0.00
Methylprednisolone	-29.4%	-1.1%	0.28	-29.4%	-1.2%	0.28
Prednisolone	-29.4%	2.8%	0.35	-29.4%	2.8%	0.35

ISH, International St. Mary's Hospital; PS, Propensity Score; Std. Diff, Standardized Difference.

Supplementary Table S5A. Baseline characteristics before and after propensity score adjustment: fluoroquinolones vs third-generation cephalosporins (JCMJ)

Characteristic	Before PS adjustment			After PS adjustment		
	Target (%)	Comparator (%)	Std. Diff	Target (%)	Comparator (%)	Std. Diff
**Age group**						
25-29	-2.8%	-4.6%	0.10	-5.1%	-4.7%	0.24
35-39	-2.8%	-4.6%	-0.12	-5.1%	-4.7%	-0.07
40-44	-2.8%	-4.6%	0.07	-5.1%	-4.7%	0.08
45-49	-2.8%	-4.6%	-0.17	-5.1%	-4.7%	-0.12
50-54	6.1%	-4.6%	0.16	-5.1%	-4.7%	0.03
55-59	6.6%	5.5%	0.05	8.2%	6.1%	0.08
60-64	8.8%	11.9%	-0.10	9.2%	11.7%	-0.08
70-74	9.4%	11.0%	-0.05	8.2%	11.2%	-0.10
75-79	18.2%	12.8%	0.15	16.3%	12.8%	0.10
80-84	11.6%	18.3%	-0.19	15.3%	14.3%	0.03
85-89	11.6%	11.0%	0.02	9.2%	12.2%	-0.10
90-94	3.9%	-4.6%	0.06	-5.1%	-4.7%	-0.07
**Index Year**						
2015	2.8%	4.6%	-0.10	-5.1%	-4.7%	-0.08
2016	6.6%	4.6%	0.09	6.1%	5.1%	0.04
2017	7.2%	6.4%	0.03	10.2%	7.1%	0.11
2018	5.0%	11.9%	-0.25	8.2%	9.2%	-0.04
2019	6.6%	10.1%	-0.13	9.2%	10.7%	-0.05
2020	9.4%	11.0%	-0.05	11.2%	11.7%	-0.02
2021	3.9%	5.5%	-0.08	5.1%	5.6%	-0.02
2022	14.4%	17.4%	-0.08	16.3%	18.4%	-0.05
**Comorbidities**						
Acute exacerbation of chronic obstructive airways disease	-2.8%	-4.6%	0.10	-5.1%	-4.7%	0.08
Acute renal failure syndrome	8.3%	13.8%	-0.18	9.2%	12.8%	-0.11
Bacterial pneumonia	5.0%	5.5%	-0.02	7.1%	5.6%	0.06
Chronic obstructive lung disease	6.6%	-4.6%	0.18	6.1%	-4.7%	0.15
Congestive heart failure	-2.8%	-4.6%	-0.08	-5.1%	-4.7%	0.03
Heart failure	11.0%	9.2%	0.06	11.2%	9.2%	0.07
Hypertensive heart disease with congestive heart failure	-2.8%	-4.6%	-0.06	-5.1%	-4.7%	-0.05
Mild chronic obstructive pulmonary disease	-2.8%	-4.6%	0.07	-5.1%	-4.7%	0.08
Pneumonia	47.0%	33.9%	0.27	36.7%	37.8%	-0.02
Pulmonary edema	3.9%	5.5%	-0.08	-5.1%	5.1%	-0.05
Septic shock	4.4%	-4.6%	0.04	-5.1%	-4.7%	0.03
Viral pneumonia	-2.8%	-4.6%	-0.09	-5.1%	-4.7%	-0.17
**Medications**						
Dexamethasone	6.1%	-4.6%	0.11	-5.1%	-4.7%	-0.06

JCMJ, Jecheon Myongji Hospital; PS, Propensity Score; Std. Diff, Standardized Difference.

Supplementary Table S5B. Baseline characteristics before and after propensity score adjustment: aminopenicillin/β-lactamase inhibitor combinations vs third-generation cephalosporins (JCMJ)

Characteristic	Before PS adjustment			After PS adjustment		
	Target (%)	Comparator	Std. Diff	Target (%)	Comparator	Std. Diff
**Age group**						
25-29	-17.2%	-4.6%	0.51	-17.2%	-3.3%	0.50
30-34	-17.2%	-4.6%	0.04	-17.2%	4.0%	-0.03
35-39	-17.2%	-4.6%	0.31	-17.2%	4.0%	0.25
40-44	-17.2%	-4.6%	0.31	-17.2%	-3.3%	0.26
45-49	-17.2%	-4.6%	0.26	-17.2%	5.2%	0.19
50-54	-17.2%	-4.6%	0.19	-17.2%	3.4%	0.16
55-59	-17.2%	5.5%	0.18	-17.2%	6.0%	0.16
60-64	-17.2%	11.9%	-0.32	-17.2%	14.6%	-0.40
70-74	20.7%	11.0%	0.27	20.7%	11.8%	0.24
75-79	-17.2%	12.8%	-0.20	-17.2%	9.7%	-0.10
80-84	-17.2%	18.3%	-0.35	-17.2%	18.9%	-0.36
**Index Year**						
2015	-17.2%	4.6%	-0.06	-17.2%	4.0%	-0.03
2016	-17.2%	4.6%	-0.06	-17.2%	5.4%	-0.10
2017	17.2%	6.4%	0.34	17.2%	4.6%	0.42
2018	20.7%	11.9%	0.24	20.7%	12.3%	0.23
2019	-17.2%	10.1%	-0.27	-17.2%	10.6%	-0.28
2020	17.2%	11.0%	0.18	17.2%	11.2%	0.17
2021	-17.2%	5.5%	-0.10	-17.2%	7.2%	-0.17
2022	-17.2%	17.4%	-0.21	-17.2%	20.4%	-0.28
2023	-17.2%	26.6%	-0.43	-17.2%	22.9%	-0.34
2024	-17.2%	-4.6%	0.17	-17.2%	-3.3%	0.24
**Comorbidities**						
Acute hypercapnic respiratory failure	-17.2%	-4.6%	0.10	-17.2%	-3.3%	0.13
Ascites	-17.2%	-4.6%	0.10	-17.2%	-3.3%	0.07
Ascites due to alcoholic cirrhosis	-17.2%	-4.6%	0.10	-17.2%	-3.3%	0.07
Bacterial pneumonia	-17.2%	5.5%	0.06	-17.2%	4.3%	0.11
Bronchopneumonia	-17.2%	-4.6%	0.17	-17.2%	-3.3%	0.15
Chronic obstructive lung disease	-17.2%	-4.6%	0.04	-17.2%	-3.3%	0.18
Congestive heart failure	-17.2%	-4.6%	0.19	-17.2%	3.4%	0.16
Decompensated cirrhosis of liver	-17.2%	-4.6%	0.17	-17.2%	-3.3%	0.15
Heart failure	-17.2%	9.2%	-0.08	-17.2%	6.9%	0.00
Mild chronic obstructive pulmonary disease	-17.2%	-4.6%	0.17	-17.2%	-3.3%	0.24
Pulmonary edema	-17.2%	5.5%	-0.10	-17.2%	5.7%	-0.11
Shock	-17.2%	-4.6%	0.17	-17.2%	-3.3%	0.15
**Medications**						
Dexamethasone	-17.2%	-4.6%	-0.01	-17.2%	-3.3%	0.03
Methylprednisolone	-17.2%	-4.6%	0.04	-17.2%	-3.3%	0.18
Prednisolone	-17.2%	-4.6%	0.31	-17.2%	-3.3%	0.30

JCMJ, Jecheon Myongji Hospital; PS, Propensity Score; Std. Diff, Standardized Difference.

Supplementary Table S6A. Baseline characteristics before and after propensity score adjustment: fluoroquinolones vs third-generation cephalosporins (KDH)

Characteristic	Before PS adjustment			After PS adjustment		
	Target (%)	Comparator (%)	Std. Diff	Target (%)	Comparator (%)	Std. Diff
**Age group**						
35-39	-4.5%	-4.5%	-0.08	-6.6%	-3.6%	-0.05
40-44	-4.5%	-4.5%	-0.06	-6.6%	-3.6%	-0.04
45-49	4.5%	-4.5%	0.05	-6.6%	-3.6%	0.10
50-54	-4.5%	-4.5%	0.06	-6.6%	-3.6%	-0.09
55-59	4.5%	6.3%	-0.08	-6.6%	6.2%	-0.04
60-64	9.0%	8.1%	0.03	7.9%	8.6%	-0.02
65-69	8.1%	10.8%	-0.09	-6.6%	9.0%	-0.15
70-74	9.0%	7.2%	0.07	9.2%	8.6%	0.02
75-79	14.4%	13.5%	0.03	10.5%	13.2%	-0.08
80-84	17.1%	16.2%	0.02	19.7%	15.1%	0.12
85-89	17.1%	14.4%	0.07	17.1%	13.0%	0.11
90-94	4.5%	5.4%	-0.04	6.6%	7.2%	-0.03
**Index Year**						
2021	12.6%	7.2%	0.18	10.5%	6.6%	0.14
2022	45.0%	55.0%	-0.20	40.8%	57.9%	-0.35
2024	13.5%	11.7%	0.05	13.2%	12.3%	0.03
**Comorbidities**						
Acute respiratory failure	-4.5%	-4.5%	0.14	-6.6%	-3.6%	0.09
Ascites	-4.5%	-4.5%	-0.06	-6.6%	-3.6%	-0.01
Ascites due to alcoholic cirrhosis	-4.5%	-4.5%	-0.14	-6.6%	-3.6%	-0.07
Chronic obstructive lung disease	5.4%	4.5%	0.04	6.6%	5.9%	0.03
Heart failure	5.4%	8.1%	-0.11	-6.6%	8.4%	-0.25
Hepatic failure	-4.5%	-4.5%	-0.08	-6.6%	-3.6%	-0.05
Hypertensive heart disease with congestive heart failure	-4.5%	-4.5%	-0.18	-6.6%	3.9%	-0.16
Hypertensive renal failure	-4.5%	-4.5%	-0.18	-6.6%	-3.6%	-0.11
Moderate chronic obstructive pulmonary disease	-4.5%	-4.5%	0.14	-6.6%	-3.6%	0.12
Pneumonia	41.4%	27.9%	0.29	30.3%	34.5%	-0.09
Pulmonary embolism	-4.5%	-4.5%	-0.11	-6.6%	3.9%	-0.07
Pulmonary emphysema	-4.5%	-4.5%	0.08	-6.6%	-3.6%	0.10
Viral pneumonia	-4.5%	-4.5%	0.08	-6.6%	-3.6%	0.07
**Medications**						
Methylprednisolone	-4.5%	-4.5%	0.08	-6.6%	-3.6%	0.16
Prednisolone	-4.5%	5.4%	-0.19	-6.6%	4.6%	-0.20

KDH, Kangdong Sacred Heart Hospital; PS, Propensity Score; Std. Diff, Standardized Difference.

Supplementary Table S6B. Baseline characteristics before and after propensity score adjustment: aminopenicillin/β-lactamase inhibitor combinations vs third-generation cephalosporins (KDH)

Characteristic	Before PS adjustment			After PS adjustment		
	Target	Comparator	Std. Diff	Target (%)	Comparator	Std.
**Age group**						
20-24	-38.5%	-4.5%	0.50	-38.5%	-6.9%	0.51
25-29	-38.5%	-4.5%	0.28	-38.5%	-6.9%	0.23
30-34	-38.5%	-4.5%	0.34	-38.5%	-6.9%	0.30
35-39	-38.5%	-4.5%	0.28	-38.5%	-6.9%	0.30
55-59	-38.5%	6.3%	0.05	-38.5%	6.9%	0.03
65-69	-38.5%	10.8%	0.14	-38.5%	8.2%	0.22
70-74	-38.5%	7.2%	0.02	-38.5%	8.7%	-0.04
75-79	-38.5%	13.5%	-0.19	-38.5%	12.6%	-0.16
80-84	-38.5%	16.2%	-0.27	-38.5%	16.7%	-0.28
**Index Year**						
2022	61.5%	55.0%	0.13	61.5%	58.5%	0.06
2023	-38.5%	26.1%	0.10	-38.5%	18.2%	0.29
2024	-38.5%	11.7%	-0.14	-38.5%	13.6%	-0.19
**Comorbidities**						
Heart failure	-38.5%	8.1%	-0.02	-38.5%	-6.9%	0.30
Pneumococcal pneumonia	-38.5%	-4.5%	0.34	-38.5%	-6.9%	0.31
Pneumonia	-38.5%	27.9%	-0.31	-38.5%	27.2%	-0.29
**Medications**						
Dexamethasone	-38.5%	22.5%	-0.42	-38.5%	20.5%	-0.37
Methylprednisolone	-38.5%	-4.5%	0.34	-38.5%	-6.9%	0.30
Prednisolone	-38.5%	5.4%	0.09	-38.5%	-6.9%	0.14

KDH, Kangdong Sacred Heart Hospital; PS, Propensity Score; Std. Diff, Standardized Difference.

Supplementary Table S7A. Baseline characteristics before and after propensity score adjustment: fluoroquinolones vs third-generation cephalosporins (KHMC)

Characteristic	Before PS adjustment			After PS adjustment		
	Target (%)	Comparator (%)	Std. Diff	Target (%)	Comparator (%)	Std. Diff
**Age group**						
20-24	-7.2%	-3.1%	0.08	-8.5%	-2.1%	0.14
35-39	-7.2%	-3.1%	-0.03	-8.5%	-2.1%	0.06
40-44	-7.2%	-3.1%	0.02	-8.5%	3.3%	0.01
45-49	-7.2%	3.1%	0.13	-8.5%	3.6%	0.15
50-54	-7.2%	3.1%	-0.11	-8.5%	-2.1%	0.00
55-59	-7.2%	6.3%	-0.02	-8.5%	8.7%	-0.14
60-64	10.1%	6.9%	0.12	10.2%	4.0%	0.24
70-74	8.7%	13.8%	-0.16	10.2%	15.6%	-0.16
75-79	18.8%	17.0%	0.05	15.3%	15.9%	-0.02
80-84	18.8%	16.4%	0.07	18.6%	20.2%	-0.04
85-89	17.4%	13.8%	0.10	20.3%	13.6%	0.18
90-94	-7.2%	-3.1%	-0.08	-8.5%	-2.1%	0.00
**Index Year**						
2021	-7.2%	19.5%	-0.42	-8.5%	8.2%	-0.06
2022	20.3%	39.0%	-0.42	22.0%	20.2%	0.05
2023	46.4%	30.2%	0.34	54.2%	45.7%	0.17
2024	27.5%	10.1%	0.46	16.9%	23.7%	-0.17
**Comorbidities**						
Acidosis	-7.2%	3.1%	-0.11	-8.5%	2.7%	-0.07
Acute renal failure syndrome	11.6%	17.0%	-0.15	13.6%	15.2%	-0.05
Bacterial pneumonia	-7.2%	-3.1%	0.02	-8.5%	2.4%	-0.05
Bronchopneumonia	-7.2%	-3.1%	-0.08	-8.5%	4.7%	-0.17
Chronic obstructive lung disease	7.2%	5.7%	0.07	-8.5%	4.4%	0.03
Chronic renal failure	-7.2%	3.1%	-0.11	-8.5%	2.2%	-0.04
End stage renal disease on dialysis due to type 2 diabetes mellitus	-7.2%	-3.1%	0.02	-8.5%	-2.1%	0.14
End-stage renal disease	-7.2%	-3.1%	-0.08	-8.5%	-2.1%	0.03
Heart failure	-7.2%	12.6%	-0.30	-8.5%	10.6%	-0.21
Hepatic failure	-7.2%	3.8%	-0.15	-8.5%	3.7%	-0.12
Hypertensive heart disease with congestive heart failure	-7.2%	6.3%	-0.25	-8.5%	5.5%	-0.21
Hypertensive renal failure	-7.2%	-3.1%	0.17	-8.5%	-2.1%	0.18
Idiopathic pulmonary fibrosis	-7.2%	-3.1%	0.02	-8.5%	-2.1%	0.11
Localized edema	-7.2%	-3.1%	0.19	-8.5%	-2.1%	0.11
Moderate chronic obstructive pulmonary disease	-7.2%	3.1%	0.13	-8.5%	-2.1%	0.11
Pneumonia	39.1%	30.8%	0.18	32.2%	26.5%	0.13
Pulmonary edema	-7.2%	-3.1%	-0.03	-8.5%	2.5%	-0.06
Pulmonary embolism	-7.2%	-3.1%	0.02	-8.5%	-2.1%	0.16
Severe chronic obstructive pulmonary disease	-7.2%	-3.1%	0.08	-8.5%	-2.1%	0.15
Thrombophlebitis of deep veins of lower extremity	-7.2%	-3.1%	-0.03	-8.5%	-2.1%	0.11
**Medications**						
Dexamethasone	7.2%	27.0%	-0.54	8.5%	10.1%	-0.06
Hydrocortisone	-7.2%	-3.1%	0.08	-8.5%	-2.1%	0.08
Methylprednisolone	-7.2%	-3.1%	0.25	-8.5%	-2.1%	0.19
Prednisolone	-7.2%	5.0%	-0.03	-8.5%	4.3%	0.04

KHMC, Kyunghee University Medical Center; PS, Propensity Score; Std. Diff, Standardized Difference.

Supplementary Table S7B. Baseline characteristics before and after propensity score adjustment: aminopenicillin/β-lactamase inhibitor combinations vs third-generation cephalosporins (KHMC)

Characteristic	Before PS adjustment			After PS adjustment		
	Target (%)	Comparator (%)	Std. Diff	Target (%)	Comparator (%)	Std. Diff
**Age group**						
15-19	-18.5%	-3.1%	0.21	-38.5%	-2.9%	0.38
45-49	-18.5%	3.1%	0.19	-38.5%	9.2%	-0.06
50-54	-18.5%	3.1%	0.03	-38.5%	3.3%	0.19
55-59	-18.5%	6.3%	-0.12	-38.5%	11.3%	-0.12
60-64	29.6%	6.9%	0.62	-38.5%	22.8%	-0.19
70-74	-18.5%	13.8%	-0.36	-38.5%	8.7%	-0.04
75-79	-18.5%	17.0%	-0.06	-38.5%	14.7%	0.39
80-84	-18.5%	16.4%	-0.43	-38.5%	6.3%	0.06
85-89	-18.5%	13.8%	-0.36	-38.5%	9.4%	-0.06
**Index Year**						
2022	63.0%	39.0%	0.49	53.8%	47.1%	0.13
2023	25.9%	30.2%	-0.10	-38.5%	32.9%	-0.22
2024	-18.5%	10.1%	0.03	-38.5%	15.8%	0.18
**Comorbidities**						
Acute renal failure due to tubular necrosis	-18.5%	-3.1%	0.21	-38.5%	-2.9%	0.30
Acute renal failure syndrome	-18.5%	17.0%	-0.45	-38.5%	7.4%	0.01
Heart failure	-18.5%	12.6%	-0.17	-38.5%	7.2%	0.02
Hypertensive heart disease with congestive heart failure	-18.5%	6.3%	-0.12	-38.5%	-2.9%	0.33

KHMC, Kyunghee University Medical Center; PS, Propensity Score; Std. Diff, Standardized Difference.

Supplementary Table S8A. Baseline characteristics before and after propensity score adjustment: fluoroquinolones vs third-generation cephalosporins (KWMC)

Characteristic	Before PS adjustment			After PS adjustment		
	Target (%)	Comparator (%)	Std. Diff	Target (%)	Comparator (%)	Std. Diff
**Age group**						
20-24	-2.2%	-2.0%	-0.03	-4.1%	-1.7%	-0.05
30-34	-2.2%	2.0%	-0.05	-4.1%	-1.7%	-0.01
40-44	-2.2%	-2.0%	-0.02	-4.1%	-1.7%	0.06
45-49	-2.2%	-2.0%	0.01	-4.1%	1.8%	0.05
50-54	2.7%	3.2%	-0.03	-4.1%	2.5%	-0.06
55-59	4.0%	4.4%	-0.02	5.7%	3.2%	0.12
60-64	3.1%	3.2%	-0.01	-4.1%	3.0%	0.02
65-69	8.0%	6.4%	0.06	8.1%	6.9%	0.05
70-74	8.4%	7.2%	0.05	7.3%	8.1%	-0.03
75-79	13.3%	15.5%	-0.06	16.3%	15.3%	0.03
80-84	22.1%	21.1%	0.02	19.5%	25.8%	-0.15
85-89	16.4%	15.5%	0.02	14.6%	13.2%	0.04
90-94	11.5%	9.6%	0.06	14.6%	9.7%	0.15
95-99	4.4%	2.8%	0.09	-4.1%	3.4%	-0.06
**Index Year**						
2020	-2.2%	-2.0%	0.13	-4.1%	-1.7%	0.13
2021	10.6%	2.0%	0.36	-4.1%	3.7%	0.02
2022	53.5%	43.4%	0.20	47.2%	44.9%	0.05
2023	34.1%	54.2%	-0.41	46.3%	50.6%	-0.09
**Comorbidities**						
Acidosis	3.5%	2.0%	0.09	-4.1%	2.4%	-0.06
Acute exacerbation of chronic obstructive pulmonary disease	-2.2%	-2.0%	0.01	-4.1%	-1.7%	-0.02
Acute kidney injury	23.0%	19.9%	0.08	22.0%	22.4%	-0.01
Acute pulmonary edema	-2.2%	-2.0%	-0.05	-4.1%	-1.7%	0.03
Acute respiratory distress syndrome	2.7%	-2.0%	0.19	-4.1%	-1.7%	0.13
Agranulocytosis	-2.2%	-2.0%	0.05	-4.1%	-1.7%	0.12
Ascites	-2.2%	-2.0%	-0.06	-4.1%	-1.7%	-0.04
Ascites due to alcoholic cirrhosis	-2.2%	-2.0%	-0.03	-4.1%	-1.7%	0.03
Bacterial pneumonia	2.2%	2.0%	0.02	-4.1%	-1.7%	0.00
Blood coagulation disorder	-2.2%	-2.0%	-0.05	-4.1%	-1.7%	0.05
Chronic obstructive pulmonary disease	4.4%	4.8%	-0.02	-4.1%	5.6%	-0.07
Chronic obstructive pulmonary disease with acute lower respiratory infection	-2.2%	-2.0%	-0.06	-4.1%	2.0%	-0.03
Congestive heart failure	2.2%	-2.0%	0.12	-4.1%	-1.7%	0.06
Diastolic heart failure	3.1%	-2.0%	0.13	-4.1%	1.8%	0.09
End stage renal disease on dialysis due to type 2 diabetes mellitus	-2.2%	-2.0%	-0.05	-4.1%	-1.7%	-0.04
Heart failure	12.8%	12.0%	0.03	13.8%	12.9%	0.03
Hypertensive heart disease with congestive heart failure	-2.2%	-2.0%	0.10	-4.1%	-1.7%	0.05
Hypovolemic shock	-2.2%	-2.0%	-0.05	-4.1%	-1.7%	0.03
Left heart failure	3.1%	2.8%	0.02	-4.1%	4.0%	0.00
Mild chronic obstructive pulmonary disease	-2.2%	-2.0%	-0.12	-4.1%	-1.7%	-0.06
Moderate chronic obstructive pulmonary disease	4.0%	2.8%	0.07	-4.1%	3.9%	0.01
Pneumonia	63.7%	40.2%	0.48	57.7%	61.7%	-0.08
Pulmonary edema	2.7%	3.6%	-0.05	-4.1%	4.7%	-0.18
Pulmonary embolism	2.2%	-2.0%	0.16	-4.1%	-1.7%	0.13
Renal failure syndrome	-2.2%	2.4%	-0.04	-4.1%	2.3%	-0.05
Septic shock	6.6%	-2.0%	0.31	-4.1%	-1.7%	0.05
Severe chronic obstructive pulmonary disease	-2.2%	-2.0%	0.01	-4.1%	2.0%	0.08
Shock	-2.2%	-2.0%	0.10	-4.1%	-1.7%	0.07
Thrombophlebitis of deep veins of lower extremity	2.2%	2.0%	0.02	-4.1%	2.9%	-0.03
Viral pneumonia	25.2%	10.4%	0.40	18.7%	16.9%	0.05
White blood cell disorder	-2.2%	-2.0%	0.01	-4.1%	-1.7%	0.09
**Medications**						
Dexamethasone	42.5%	6.4%	0.93	13.8%	13.0%	0.02
Hydrocortisone	-2.2%	-2.0%	0.01	-4.1%	-1.7%	0.07
Methylprednisolone	4.4%	2.0%	0.14	4.9%	3.0%	0.09
Prednisolone	-2.2%	-2.0%	0.05	-4.1%	-1.7%	-0.04

KWMC, Gangwon National University Medical Center; PS, Propensity Score; Std. Diff, Standardized Difference.

Supplementary Table S8B. Baseline characteristics before and after propensity score adjustment: aminopenicillin/β-lactamase inhibitor combinations vs third-generation cephalosporins (KWMC)

Characteristic	Before PS adjustment			After PS adjustment		
	Target (%)	Comparator	Std. Diff	Target	Comparator	Std.
**Age group**						
15-19	-15.6%	-2.0%	0.30	-16.1%	1.6%	0.25
20-24	-15.6%	-2.0%	0.27	-16.1%	2.2%	0.21
25-29	-15.6%	2.0%	0.07	-16.1%	3.0%	0.01
35-39	-15.6%	2.0%	0.07	-16.1%	2.6%	0.04
45-49	-15.6%	-2.0%	0.13	-16.1%	-1.3%	0.24
50-54	-15.6%	3.2%	0.00	-16.1%	4.7%	-0.08
55-59	-15.6%	4.4%	0.30	-16.1%	5.2%	0.27
60-64	-15.6%	3.2%	0.00	-16.1%	2.5%	0.05
65-69	-15.6%	6.4%	0.11	-16.1%	5.5%	0.16
70-74	-15.6%	7.2%	0.08	-16.1%	4.9%	0.18
75-79	-15.6%	15.5%	-0.30	-16.1%	12.2%	-0.20
80-84	-15.6%	21.1%	-0.57	-16.1%	15.3%	-0.42
85-89	-15.6%	15.5%	-0.19	-16.1%	13.1%	-0.11
90-94	-15.6%	9.6%	-0.12	-16.1%	9.0%	-0.09
95-99	-15.6%	2.8%	0.02	-16.1%	4.6%	-0.07
**Index Year**						
2023	43.8%	54.2%	-0.21	45.2%	43.0%	0.04
**Comorbidities**						
Diastolic heart failure	-15.6%	-2.0%	0.13	-16.1%	-1.3%	0.16
Heart failure	-15.6%	12.0%	-0.20	-16.1%	8.7%	-0.08
Pneumonia	-15.6%	40.2%	-0.66	-16.1%	9.5%	0.11
Viral pneumonia	-15.6%	10.4%	-0.29	-16.1%	7.1%	-0.17
**Medications**						
Dexamethasone	-15.6%	6.4%	0.11	-16.1%	6.1%	0.14
Prednisolone	-15.6%	-2.0%	0.27	-16.1%	-1.3%	0.28

KWMC, Gangwon National University Medical Center; PS, Propensity Score; Std. Diff, Standardized Difference.

Supplementary Table S9A. Baseline characteristics before and after propensity score adjustment: fluoroquinolones vs third-generation cephalosporins (MJH)

Characteristic	Before PS adjustment			After PS adjustment		
	Target (%)	Comparator (%)	Std. Diff	Target (%)	Comparator (%)	Std. Diff
**Age group**						
20-24	-2.5%	-2.5%	0.06	-3.8%	-2.4%	0.14
25-29	-2.5%	-2.5%	-0.06	-3.8%	-2.4%	0.03
40-44	-2.5%	-2.5%	-0.06	-3.8%	-2.4%	0.02
45-49	-2.5%	-2.5%	-0.10	-3.8%	-2.4%	-0.09
50-54	-2.5%	2.9%	-0.14	-3.8%	3.4%	-0.12
55-59	3.4%	3.9%	-0.03	4.6%	4.2%	0.02
60-64	4.4%	5.4%	-0.05	-3.8%	5.1%	-0.10
70-74	8.3%	5.9%	0.10	9.2%	6.1%	0.12
75-79	15.2%	14.7%	0.01	13.0%	14.1%	-0.03
80-84	19.6%	22.5%	-0.07	18.3%	21.8%	-0.09
85-89	22.1%	17.6%	0.11	20.6%	19.3%	0.03
90-94	9.8%	10.8%	-0.03	10.7%	10.8%	0.00
95-99	5.9%	2.9%	0.14	6.9%	3.8%	0.14
**Index Year**						
2020	-2.5%	3.9%	-0.12	-3.8%	3.8%	-0.09
2022	36.3%	38.2%	-0.04	38.2%	38.4%	-0.01
2023	35.8%	38.2%	-0.05	37.4%	37.8%	-0.01
2024	20.1%	13.7%	0.17	15.3%	14.1%	0.03
**Comorbidities**						
Acidosis	-2.5%	-2.5%	0.06	-3.8%	-2.4%	0.07
Acute exacerbation of chronic obstructive airways disease	-2.5%	-2.5%	0.13	-3.8%	-2.4%	0.05
Acute renal failure syndrome	12.7%	10.8%	0.06	10.7%	8.1%	0.09
Ascites	-2.5%	-2.5%	-0.06	-3.8%	-2.4%	-0.03
Bacterial pneumonia	2.5%	-2.5%	0.11	-3.8%	-2.4%	0.06
Bronchopneumonia	-2.5%	-2.5%	0.06	-3.8%	-2.4%	0.05
Chronic obstructive lung disease	4.9%	4.4%	0.02	3.8%	5.7%	-0.09
Congestive heart failure	7.8%	-2.5%	0.28	3.8%	3.1%	0.04
Heart failure	17.6%	14.7%	0.08	15.3%	14.6%	0.02
Hepatic failure	2.5%	2.9%	-0.03	-3.8%	2.7%	-0.08
Hypertensive heart disease with congestive heart failure	3.4%	2.5%	0.06	3.8%	3.3%	0.03
Left heart failure	14.7%	15.2%	-0.01	13.0%	16.4%	-0.10
Moderate chronic obstructive pulmonary disease	-2.5%	-2.5%	0.08	-3.8%	-2.4%	0.03
Pneumonia	37.7%	19.6%	0.41	28.2%	29.0%	-0.02
Pulmonary emphysema	-2.5%	-2.5%	0.13	-3.8%	-2.4%	0.07
Renal failure syndrome	-2.5%	-2.5%	0.05	-3.8%	-2.4%	0.03
Right ventricular failure	-2.5%	-2.5%	0.05	-3.8%	-2.4%	0.02
Septic shock	5.9%	-2.5%	0.27	-3.8%	-2.4%	0.06
Severe chronic obstructive pulmonary disease	2.9%	-2.5%	0.10	-3.8%	-2.4%	0.11
Viral pneumonia	28.4%	13.2%	0.38	20.6%	20.2%	0.01
**Medications**						
deflazacort	-2.5%	-2.5%	0.06	-3.8%	-2.4%	0.07
Hydrocortisone	-2.5%	-2.5%	-0.06	-3.8%	-2.4%	0.02
Methotrexate	-2.5%	-2.5%	0.10	-3.8%	-2.4%	0.14
Methylprednisolone	6.4%	4.4%	0.09	6.9%	5.2%	0.07
Prednisolone	2.9%	2.5%	0.03	-3.8%	3.3%	-0.12

MJH, Myongji Hospital; PS, Propensity Score; Std. Diff, Standardized Difference.

Supplementary Table S9B. Baseline characteristics before and after propensity score adjustment: aminopenicillin/β-lactamase inhibitor combinations vs third-generation cephalosporins (MJH)

Characteristic	Before PS adjustment			After PS adjustment		
	Target	Comparator	Std. Diff	Target (%)	Comparator	Std.
**Age group**						
40-44	-45.5%	-2.5%	0.38	-45.5%	-2.4%	0.38
45-49	-45.5%	-2.5%	0.59	-45.5%	-2.4%	0.59
60-64	-45.5%	5.4%	0.41	-45.5%	5.4%	0.40
65-69	-45.5%	7.4%	0.33	-45.5%	7.3%	0.33
70-74	-45.5%	5.9%	0.39	-45.5%	5.9%	0.38
80-84	-45.5%	22.5%	-0.38	-45.5%	22.5%	-0.37
90-94	-45.5%	10.8%	-0.06	-45.5%	10.7%	-0.06
**Index Year**						
2021	54.5%	5.4%	1.27	54.5%	5.4%	1.27
2022	-45.5%	38.2%	-0.04	-45.5%	38.2%	-0.04
2024	-45.5%	13.7%	-0.15	-45.5%	13.9%	-0.15
**Comorbidities**						
Acute renal failure syndrome	-45.5%	10.8%	-0.06	-45.5%	10.7%	-0.06
Acute respiratory failure	-45.5%	-2.5%	0.41	-45.5%	-2.4%	0.41
Bacterial pneumonia	-45.5%	-2.5%	0.38	-45.5%	-2.4%	0.38
Congestive heart failure	-45.5%	-2.5%	0.32	-45.5%	-2.4%	0.31
Left heart failure	-45.5%	15.2%	0.08	-45.5%	15.1%	0.08
Renal failure syndrome	-45.5%	-2.5%	0.38	-45.5%	-2.4%	0.38
Viral pneumonia	45.5%	13.2%	0.76	45.5%	13.2%	0.76
**Medications**						
Dexamethasone	54.5%	20.1%	0.76	54.5%	20.0%	0.76
Prednisolone	-45.5%	2.5%	0.29	-45.5%	2.5%	0.29

MJH, Myongji Hospital; PS, Propensity Score; Std. Diff, Standardized Difference.

Supplementary Table S10A. Baseline characteristics before and after propensity score adjustment: fluoroquinolones vs third-generation cephalosporins (SCHBC)

Characteristic	Before PS adjustment			After PS adjustment		
	Target (%)	Comparator (%)	Std. Diff	Target (%)	Comparator (%)	Std. Diff
**Age group**						
25-29	-11.4%	-3.2%	0.07	-11.6%	-3.2%	0.07
35-39	-11.4%	-3.2%	0.15	-11.6%	-3.2%	0.15
40-44	-11.4%	3.9%	0.03	-11.6%	3.9%	0.04
45-49	-11.4%	3.9%	-0.09	-11.6%	3.5%	-0.07
50-54	-11.4%	4.5%	-0.12	-11.6%	4.7%	-0.13
55-59	11.4%	5.2%	0.23	11.6%	5.0%	0.24
60-64	-11.4%	16.8%	-0.51	-11.6%	17.4%	-0.52
65-69	15.9%	13.5%	0.07	14.0%	13.4%	0.02
70-74	-11.4%	7.7%	0.05	-11.6%	7.8%	0.06
75-79	-11.4%	8.4%	0.03	-11.6%	8.1%	0.04
85-89	15.9%	12.3%	0.11	16.3%	12.6%	0.11
90-94	-11.4%	4.5%	0.18	-11.6%	4.7%	0.18
**Index Year**						
2021	-11.4%	7.1%	-0.01	-11.6%	7.4%	-0.11
2022	79.5%	66.5%	0.30	81.4%	66.7%	0.34
2023	13.6%	22.6%	-0.23	14.0%	22.3%	-0.22
**Comorbidities**						
Acidosis	-11.4%	-3.2%	0.02	-11.6%	-3.2%	0.04
Acute exacerbation of chronic obstructive airways disease	-11.4%	-3.2%	-0.02	-11.6%	-3.2%	-0.01
Acute hypoxemic respiratory failure	-11.4%	-3.2%	0.28	-11.6%	-3.2%	0.29
Acute renal failure syndrome	27.3%	20.6%	0.16	27.9%	20.7%	0.17
Acute respiratory failure	11.4%	6.5%	0.17	11.6%	6.4%	0.18
Agranulocytosis	-11.4%	-3.2%	0.07	-11.6%	-3.2%	0.07
Ascites	-11.4%	6.5%	-0.21	-11.6%	6.8%	-0.22
Ascites due to alcoholic cirrhosis	-11.4%	5.2%	-0.15	-11.6%	5.2%	-0.15
Chronic obstructive lung disease	-11.4%	7.7%	-0.04	-11.6%	8.1%	-0.04
Congestive heart failure	15.9%	3.2%	0.44	16.3%	-3.2%	0.46
Disseminated intravascular coagulation	-11.4%	-3.2%	0.24	-11.6%	-3.2%	0.23
End stage renal disease on dialysis due to type 2 diabetes mellitus	-11.4%	7.1%	-0.01	-11.6%	7.4%	-0.02
Heart failure	-11.4%	-3.2%	0.28	-11.6%	-3.2%	0.28
Hepatic failure	-11.4%	5.8%	-0.18	-11.6%	5.8%	-0.18
Hepatorenal syndrome	-11.4%	-3.2%	0.07	-11.6%	-3.2%	0.09
Hypertensive renal failure	-11.4%	-3.2%	0.19	-11.6%	-3.2%	0.19
Mild chronic obstructive pulmonary disease	-11.4%	-3.2%	0.33	-11.6%	-3.2%	0.33
Pneumonia	45.5%	34.8%	0.22	44.2%	34.1%	0.21
Pulmonary edema	-11.4%	-3.2%	0.40	-11.6%	-3.2%	0.40
Pulmonary embolism	-11.4%	-3.2%	0.07	-11.6%	-3.2%	0.07
Pulmonary emphysema	-11.4%	3.2%	-0.06	-11.6%	3.3%	-0.06
Septic shock	-11.4%	-3.2%	0.28	-11.6%	-3.2%	0.28
Systolic heart failure	-11.4%	-3.2%	0.25	-11.6%	-3.2%	0.24
Thrombophlebitis of deep veins of lower extremity	-11.4%	9.0%	-0.18	-11.6%	9.7%	-0.20
Viral pneumonia	18.2%	17.4%	0.02	18.6%	15.9%	0.07
**Medications**						
Dexamethasone	11.4%	24.5%	-0.35	11.6%	24.4%	-0.34
Methylprednisolone	-11.4%	5.2%	0.15	-11.6%	5.2%	0.07
Prednisolone	-11.4%	3.9%	0.03	-11.6%	4.3%	0.02

SCHBC, Soonchunhyang University Hospital Bucheon Center; PS, Propensity Score; Std. Diff, Standardized Difference.

Supplementary Table S11A. Baseline characteristics before and after propensity score adjustment: fluoroquinolones vs third-generation cephalosporins (SCHCA)

Characteristic	Before PS adjustment			After PS adjustment		
	Target (%)	Comparator (%)	Std. Diff	Target (%)	Comparator (%)	Std. Diff
**Age group**						
20-24	-8.8%	-2.7%	0.01	-9.8%	-2.4%	0.04
25-29	-8.8%	-2.7%	0.31	-9.8%	-2.4%	0.34
35-39	-8.8%	-2.7%	0.12	-9.8%	-2.4%	0.18
45-49	-8.8%	2.7%	0.05	-9.8%	2.5%	0.08
50-54	-8.8%	7.5%	-0.18	-9.8%	5.9%	-0.09
55-59	-8.8%	7.0%	-0.26	-9.8%	10.1%	-0.35
60-64	-8.8%	8.6%	-0.13	-9.8%	6.6%	-0.03
65-69	15.8%	10.2%	0.17	11.8%	12.9%	-0.04
70-74	10.5%	8.6%	0.07	11.8%	8.7%	0.10
75-79	12.3%	12.3%	0.00	11.8%	12.3%	-0.02
80-84	21.1%	19.8%	0.03	19.6%	20.2%	-0.02
85-89	-8.8%	10.7%	-0.13	-9.8%	6.5%	0.05
90-94	-8.8%	4.3%	0.12	-9.8%	4.9%	0.12
**Index Year**						
2021	8.8%	11.2%	-0.08	-9.8%	8.9%	-0.04
2022	78.9%	75.9%	0.07	82.4%	77.5%	0.12
2023	10.5%	12.3%	-0.06	-9.8%	11.6%	-0.13
**Comorbidities**						
Acute exacerbation of chronic obstructive airways disease	-8.8%	-2.7%	0.29	-9.8%	-2.4%	0.34
Acute hypoxemic respiratory failure	19.3%	5.9%	0.41	11.8%	13.9%	-0.06
Acute pulmonary edema	-8.8%	-2.7%	0.06	-9.8%	-2.4%	0.04
Acute renal failure syndrome	28.1%	14.4%	0.34	27.5%	29.4%	-0.04
Acute respiratory distress syndrome	-8.8%	-2.7%	0.08	-9.8%	3.9%	-0.12
Acute respiratory failure	-8.8%	-2.7%	0.06	-9.8%	-2.4%	0.17
Ascites	-8.8%	-2.7%	-0.03	-9.8%	2.5%	-0.03
Chronic obstructive lung disease	-8.8%	4.8%	0.09	-9.8%	3.5%	0.11
Congestive heart failure	10.5%	6.4%	0.15	11.8%	10.6%	0.04
Diastolic heart failure	-8.8%	-2.7%	0.12	-9.8%	-2.4%	0.18
End stage renal disease on dialysis due to type 2 diabetes mellitus	-8.8%	2.7%	0.05	-9.8%	3.4%	0.03
Heart failure	-8.8%	-2.7%	-0.03	-9.8%	3.1%	-0.07
Hypertensive heart disease with congestive heart failure	-8.8%	-2.7%	0.21	-9.8%	-2.4%	0.27
Hypertensive renal failure	-8.8%	2.7%	-0.06	-9.8%	2.5%	-0.03
Hypovolemic shock	-8.8%	-2.7%	0.12	-9.8%	-2.4%	0.13
Idiopathic pulmonary fibrosis	-8.8%	-2.7%	0.12	-9.8%	-2.4%	0.13
Ketoacidosis due to type 2 diabetes mellitus	-8.8%	-2.7%	0.12	-9.8%	-2.4%	0.13
Moderate chronic obstructive pulmonary disease	-8.8%	-2.7%	0.08	-9.8%	2.6%	0.07
Pneumonia	45.6%	41.2%	0.09	41.2%	42.2%	-0.02
Pneumonia due to Pseudomonas	-8.8%	-2.7%	0.21	-9.8%	-2.4%	0.13
Pulmonary edema	-8.8%	2.7%	0.05	-9.8%	3.4%	0.03
Pulmonary emphysema	-8.8%	-2.7%	0.24	-9.8%	-2.4%	0.27
Renal failure syndrome	-8.8%	4.3%	-0.04	-9.8%	4.3%	-0.02
Septic shock	-8.8%	2.7%	0.20	-9.8%	-2.4%	0.19
Severe chronic obstructive pulmonary disease	-8.8%	-2.7%	0.08	-9.8%	-2.4%	0.13
Systolic heart failure	-8.8%	-2.7%	0.27	-9.8%	-2.4%	0.29
Viral pneumonia	-8.8%	9.6%	-0.25	-9.8%	6.5%	-0.23
**Medications**						
Dexamethasone	17.5%	31.0%	-0.32	15.7%	17.6%	-0.05
Hydrocortisone	-8.8%	-2.7%	0.12	-9.8%	-2.4%	0.13
Methylprednisolone	-8.8%	2.7%	0.20	-9.8%	-2.4%	0.19
Prednisolone	-8.8%	-2.7%	0.01	-9.8%	3.4%	-0.09

SCHCA, Soonchunhyang University Hospital Cheonan Center; PS, Propensity Score; Std. Diff, Standardized Difference.

Supplementary Table S12A. Baseline characteristics before and after propensity score adjustment: fluoroquinolones vs third-generation cephalosporins (WKUH)

Characteristic	Before PS adjustment			After PS adjustment		
	Target (%)	Comparator (%)	Std. Diff	Target (%)	Comparator (%)	Std. Diff
**Age group**						
30-34	-2.7%	-2.1%	0.00	-4.3%	-2.1%	0.04
40-44	-2.7%	-2.1%	0.03	-4.3%	-2.1%	0.04
45-49	-2.7%	3.8%	-0.13	-4.3%	3.6%	-0.06
50-54	-2.7%	2.5%	-0.02	-4.3%	-2.1%	0.05
55-59	4.9%	5.8%	-0.04	6.0%	5.7%	0.01
65-69	10.3%	8.3%	0.07	9.4%	7.8%	0.06
70-74	9.2%	10.0%	-0.03	8.5%	10.5%	-0.07
75-79	15.2%	12.5%	0.08	16.2%	13.2%	0.08
80-84	20.1%	17.1%	0.08	20.5%	15.8%	0.12
85-89	14.7%	14.6%	0.00	12.8%	15.4%	-0.07
90-94	5.4%	6.7%	-0.05	5.1%	5.6%	-0.02
**Index Year**						
2020	-2.7%	-2.1%	0.07	-4.3%	-2.1%	-0.07
2022	53.8%	49.2%	0.09	50.4%	44.0%	0.13
2023	39.1%	45.0%	-0.12	43.6%	48.7%	-0.10
2024	4.9%	3.8%	0.06	5.1%	5.0%	0.01
**Comorbidities**						
Acidosis	8.7%	4.6%	0.17	6.8%	6.0%	0.04
Acute hypoxemic respiratory failure	10.3%	-2.1%	0.42	-4.3%	-2.1%	0.13
Acute renal failure syndrome	16.8%	13.3%	0.10	12.8%	14.8%	-0.06
Agranulocytosis	-2.7%	-2.1%	0.04	-4.3%	-2.1%	-0.09
Ascites due to alcoholic cirrhosis	-2.7%	-2.1%	-0.04	-4.3%	-2.1%	0.03
Bacterial pneumonia	-2.7%	-2.1%	0.03	-4.3%	-2.1%	0.02
Bronchopneumonia	-2.7%	2.1%	0.01	-4.3%	2.7%	-0.14
Chronic obstructive lung disease	3.8%	2.5%	0.08	-4.3%	3.8%	-0.13
Congestive heart failure	3.3%	2.5%	0.05	-4.3%	-2.1%	0.11
Diastolic heart failure	-2.7%	-2.1%	0.12	-4.3%	-2.1%	0.14
End stage renal disease on dialysis due to type 2 diabetes mellitus	-2.7%	-2.1%	-0.05	-4.3%	-2.1%	-0.10
Heart failure	9.2%	5.4%	0.15	8.5%	5.6%	0.12
Hypertensive heart disease with congestive heart failure	-2.7%	-2.1%	0.02	-4.3%	-2.1%	0.08
Ketoacidosis due to type 2 diabetes mellitus	-2.7%	-2.1%	0.02	-4.3%	-2.1%	0.08
Left heart failure	-2.7%	-2.1%	-0.04	-4.3%	-2.1%	0.03
Localized edema	-2.7%	-2.1%	0.04	-4.3%	2.1%	0.03
Pneumonia	56.0%	25.0%	0.67	38.5%	39.3%	-0.02
Pulmonary edema	-2.7%	-2.1%	0.07	-4.3%	-2.1%	0.11
Pulmonary embolism	2.7%	-2.1%	0.07	-4.3%	-2.1%	0.03
Septic shock	6.5%	5.0%	0.07	-4.3%	6.3%	-0.09
Thrombophlebitis of deep veins of lower extremity	-2.7%	-2.1%	0.08	-4.3%	-2.1%	0.05
Viral pneumonia	17.4%	8.3%	0.27	11.1%	11.5%	-0.01
**Medications**						
Dexamethasone	12.0%	5.0%	0.25	6.0%	7.7%	-0.07
Deflazacort	-2.7%	-2.1%	-0.04	-4.3%	-2.1%	-0.04
Methylprednisolone	2.7%	-2.1%	0.11	-4.3%	-2.1%	0.09
Prednisolone	3.3%	3.3%	0.00	-4.3%	2.1%	0.08

WKUH, Wonkwang University Hospital; PS, Propensity Score; Std. Diff, Standardized Difference.

Supplementary Table S12B. Baseline characteristics before and after propensity score adjustment: aminopenicillin/β-lactamase inhibitor combinations vs third-generation cephalosporins (WKUH)

Characteristic	Before PS adjustment			After PS adjustment		
	Target (%)	Comparator (%)	Std. Diff	Target (%)	Comparator (%)	Std. Diff
**Age group**						
15-19	-29.4%	-2.1%	0.25	-29.4%	-2.4%	0.24
20-24	-29.4%	-2.1%	0.22	-29.4%	-2.4%	0.20
25-29	-29.4%	2.1%	0.20	-29.4%	2.5%	0.17
45-49	-29.4%	3.8%	0.10	-29.4%	4.3%	0.07
50-54	-29.4%	2.5%	0.37	-29.4%	2.9%	0.35
55-59	-29.4%	5.8%	0.37	-29.4%	6.8%	0.34
60-64	-29.4%	8.3%	0.28	-29.4%	9.6%	0.24
65-69	-29.4%	8.3%	-0.10	-29.4%	9.2%	-0.12
70-74	-29.4%	10.0%	0.06	-29.4%	11.0%	0.02
80-84	-29.4%	17.1%	-0.15	-29.4%	16.7%	-0.14
**Index Year**						
2022	47.1%	49.2%	-0.04	47.1%	44.6%	0.05
2023	47.1%	45.0%	0.04	47.1%	49.5%	-0.05
2024	-29.4%	3.8%	0.10	-29.4%	3.4%	0.12
**Comorbidities**						
Acidosis	-29.4%	4.6%	0.06	-29.4%	3.9%	0.09
Acute renal failure syndrome	-29.4%	13.3%	-0.26	-29.4%	12.6%	-0.23
Bacterial pneumonia	-29.4%	-2.1%	0.25	-29.4%	-2.4%	0.24
Diabetic ketoacidosis	-29.4%	-2.1%	0.28	-29.4%	-2.4%	0.27
Ketoacidosis due to type 2 diabetes mellitus	-29.4%	-2.1%	0.32	-29.4%	-2.4%	0.31
Pneumonia	-29.4%	25.0%	-0.55	-29.4%	20.2%	-0.43
**Medications**						
Dexamethasone	-29.4%	5.0%	0.04	-29.4%	4.4%	0.07
Hydrocortisone	-29.4%	-2.1%	0.32	-29.4%	-2.4%	0.31

WKUH, Wonkwang University Hospital; PS, Propensity Score; Std. Diff, Standardized Difference.

Supplementary Table S13A. Baseline characteristics before and after propensity-score adjustment with expanded severity covariates: fluoroquinolones vs third-generation cephalosporins (GNUH)

Characteristic	Before PS adjustment			After PS adjustment		
	Target (%)	Comparator (%)	Std. Diff	Target (%)	Comparator (%)	Std. Diff
**Age group**						
40-44	-4.5%	-2.6%	-0.02	-5.8%	-2.7%	0.06
45-49	-4.5%	-2.6%	0.04	-5.8%	-2.7%	0.01
50-54	-4.5%	4.2%	-0.14	-5.8%	3.9%	-0.09
55-59	-4.5%	3.1%	0.03	-5.8%	4.8%	-0.01
60-64	7.2%	7.8%	-0.02	9.3%	7.6%	0.06
65-69	7.2%	10.4%	-0.11	8.0%	11.3%	-0.11
75-79	12.6%	14.1%	-0.04	11.6%	13.0%	-0.04
80-84	20.7%	20.8%	0.00	16.3%	21.1%	-0.12
90-94	9.9%	6.2%	0.14	10.5%	5.1%	0.20
95-99	-4.5%	-2.6%	0.12	-5.8%	-2.7%	0.17
**Index Year**						
2021	-4.5%	-2.6%	0.04	-5.8%	-2.7%	0.11
2022	37.8%	50.5%	-0.26	38.4%	45.6%	-0.15
2023	50.5%	39.1%	0.23	47.7%	45.3%	0.05
2024	9.0%	7.8%	0.04	10.5%	6.9%	0.13
**Oxygenation**						
Oxygen saturation in Arterial blood by Pulse oximetry	100.0%	99.5%	0.10	100.0%	99.6%	0.09
**Comorbidities**						
Acute exacerbation of chronic obstructive pulmonary disease	-4.5%	3.6%	-0.05	-5.8%	3.7%	-0.01
Acute kidney injury	8.1%	6.8%	0.05	9.3%	4.8%	0.17
Acute respiratory distress syndrome	-4.5%	-2.6%	0.22	-5.8%	-2.7%	0.21
Acute respiratory failure	4.5%	4.2%	0.02	-5.8%	4.7%	-0.13
Bacterial pneumonia	9.9%	-2.6%	0.37	-5.8%	-2.7%	0.13
Chronic obstructive pulmonary disease	11.7%	9.4%	0.08	10.5%	8.5%	0.07
Congestive heart failure	-4.5%	-2.6%	-0.06	-5.8%	3.1%	-0.05
End-stage renal disease	4.5%	3.1%	0.07	-5.8%	2.7%	0.15
Heart failure	-4.5%	4.2%	-0.03	-5.8%	4.1%	-0.04
Left heart failure	-4.5%	4.2%	-0.14	-5.8%	3.3%	-0.06
Pneumonia	36.0%	23.4%	0.28	27.9%	33.5%	-0.12
Pulmonary embolism	-4.5%	2.6%	0.01	-5.8%	-2.7%	-0.08
Pulmonary emphysema	-4.5%	-2.6%	-0.01	-5.8%	-2.7%	-0.03
Thrombophlebitis of deep veins of lower extremity	-4.5%	-2.6%	0.08	-5.8%	2.7%	0.05
Viral pneumonia	22.5%	11.5%	0.30	15.1%	17.4%	-0.06
**Medications**						
Dexamethasone	26.1%	18.8%	0.18	22.1%	20.5%	0.04
fludrocortisone	-4.5%	-2.6%	-0.01	-5.8%	-2.7%	-0.09
Methylprednisolone	5.4%	-2.6%	0.21	-5.8%	-2.7%	0.15
Prednisolone	-4.5%	3.1%	-0.09	-5.8%	-2.7%	-0.01

GNUH, Gyeongsang National University Hospital; PS, Propensity Score; Std. Diff, Standardized Difference.

Supplementary Table S13B. Baseline characteristics before and after propensity-score adjustment with expanded severity covariates: aminopenicillin/β-lactamase inhibitor combinations vs third-generation cephalosporins (GNUH)

Characteristic	Before PS adjustment			After PS adjustment		
	Target (%)	Comparator	Std. Diff	Target (%)	Comparator	Std. Diff
**Age group**						
50-54	-83.3%	4.2%	0.42	-83.3%	4.2%	0.42
55-59	-83.3%	3.1%	0.47	-83.3%	3.2%	0.46
60-64	-83.3%	7.8%	0.27	-83.3%	7.9%	0.27
65-69	-83.3%	10.4%	0.18	-83.3%	10.5%	0.18
80-84	-83.3%	20.8%	-0.11	-83.3%	21.0%	-0.11
90-94	-83.3%	6.2%	0.33	-83.3%	6.3%	0.33
**Index Year**						
2022	-83.3%	50.5%	-0.35	-83.3%	50.8%	-0.36
2023	-83.3%	39.1%	0.58	-83.3%	38.7%	0.58
**Oxygenation**						
Oxygen saturation in Arterial blood by Pulse oximetry	100.0%	99.5%	0.10	100.0%	99.5%	0.10
**Comorbidities**						
Pneumonia	-83.3%	23.4%	0.22	-83.3%	23.5%	0.22
Viral pneumonia	-83.3%	11.5%	0.15	-83.3%	11.5%	0.15

GNUH, Gyeongsang National University Hospital; PS, Propensity Score; Std. Diff, Standardized Difference.

Supplementary Table S14A. Baseline characteristics before and after propensity-score adjustment with expanded severity covariates: fluoroquinolones vs third-generation cephalosporins (ISH)

Characteristic	Before PS adjustment			After PS adjustment		
	Target (%)	Comparator (%)	Std. Diff	Target (%)	Comparator (%)	Std. Diff
**Age group**						
20-24	-8.9%	2.3%	0.23	-8.9%	4.5%	0.11
25-29	-8.9%	3.0%	0.03	-8.9%	5.4%	-0.09
30-34	-8.9%	2.1%	0.18	-8.9%	3.1%	0.11
45-49	-8.9%	3.0%	0.19	-8.9%	3.7%	0.15
50-54	-8.9%	4.8%	0.02	-8.9%	7.3%	-0.08
55-59	-8.9%	4.8%	-0.06	-8.9%	5.9%	-0.11
65-69	14.3%	6.4%	0.26	14.3%	7.4%	0.22
70-74	-8.9%	10.8%	-0.20	-8.9%	11.1%	-0.21
75-79	14.3%	12.0%	0.07	14.3%	11.2%	0.09
80-84	16.1%	13.8%	0.06	16.1%	11.5%	0.13
85-89	12.5%	16.8%	-0.12	12.5%	11.2%	0.04
90-94	-8.9%	6.7%	-0.06	-8.9%	4.0%	0.07
**Index Year**						
2020	14.3%	5.5%	0.30	14.3%	9.2%	0.16
2021	-8.9%	2.3%	-0.04	-8.9%	1.7%	0.00
2022	41.1%	31.0%	0.21	41.1%	35.6%	0.11
2023	23.2%	42.1%	-0.41	23.2%	39.8%	-0.36
2024	17.9%	17.5%	0.01	17.9%	12.4%	0.15
2025	-8.9%	1.6%	0.01	-8.9%	1.2%	0.04
**Laboratory**						
C-reactive protein	100.0%	97.7%	0.22	100.0%	95.9%	0.29
**Vital signs**						
Body temperature	51.8%	51.3%	0.01	51.8%	42.0%	0.20
Diastolic blood pressure	51.8%	55.2%	-0.07	51.8%	46.8%	0.10
Heart rate	51.8%	55.2%	-0.07	51.8%	46.8%	0.10
Systolic blood pressure	51.8%	55.2%	-0.07	51.8%	46.8%	0.10
Respiratory rate	48.2%	47.8%	0.01	48.2%	38.8%	0.19
**Oxygenation**						
Oxygen saturation in Arterial blood by Pulse oximetry	39.3%	41.6%	-0.05	39.3%	30.9%	0.18
**Comorbidities**						
Acute kidney injury	-8.9%	10.6%	-0.12	-8.9%	7.0%	0.01
Acute respiratory distress syndrome	-8.9%	1.8%	0.00	-8.9%	0.7%	0.10
Congestive heart failure	-8.9%	4.4%	-0.04	-8.9%	4.1%	-0.03
Febrile neutropenia	-8.9%	-1.1%	0.16	-8.9%	-0.7%	0.14
Heart failure	-8.9%	6.9%	-0.25	-8.9%	5.0%	-0.18
Pneumonia	28.6%	57.5%	-0.61	28.6%	27.8%	0.03
Pneumonia and influenza	-8.9%	2.3%	0.08	-8.9%	1.5%	0.14
Pulmonary embolism	-8.9%	-1.1%	0.10	-8.9%	0.8%	0.09
Respiratory failure	8.9%	15.4%	-0.20	8.9%	7.4%	0.06
Septic shock	-8.9%	2.3%	-0.04	-8.9%	1.1%	0.06
Shock	-8.9%	-1.1%	0.16	-8.9%	-0.7%	0.17
Staphylococcal pneumonia	-8.9%	-1.1%	0.16	-8.9%	-0.7%	0.17
Thrombophlebitis of deep veins of lower extremity	-8.9%	1.1%	0.05	-8.9%	-0.7%	0.12
**Medications**						
Dexamethasone	21.4%	47.6%	-0.57	21.4%	22.8%	-0.03
Methylprednisolone	-8.9%	-1.1%	0.08	-8.9%	-0.7%	0.13
Prednisolone	-8.9%	2.8%	0.05	-8.9%	2.9%	0.04

ISH, International St. Mary's Hospital; PS, Propensity Score; Std. Diff, Standardized Difference.

Supplementary Table S14B. Baseline characteristics before and after propensity-score adjustment with expanded severity covariates: aminopenicillin/β-lactamase inhibitor combinations vs third-generation cephalosporins (ISH)

Characteristic	Before PS adjustment			After PS adjustment		
	Target (%)	Comparator	Std. Diff	Target	Comparator	Std.
**Age group**						
45-49	-29.4%	3.0%	0.34	-29.4%	2.9%	0.35
50-54	-29.4%	4.8%	0.25	-29.4%	4.8%	0.25
55-59	-29.4%	4.8%	0.05	-29.4%	4.8%	0.05
60-64	-29.4%	6.7%	-0.03	-29.4%	6.7%	-0.03
65-69	-29.4%	6.4%	0.19	-29.4%	6.7%	0.18
70-74	-29.4%	10.8%	0.20	-29.4%	11.2%	0.19
75-79	-29.4%	12.0%	-0.01	-29.4%	11.9%	0.00
80-84	-29.4%	13.8%	-0.27	-29.4%	13.9%	-0.27
85-89	-29.4%	16.8%	0.02	-29.4%	17.3%	0.01
**Index Year**						
2022	58.8%	31.0%	0.58	58.8%	31.1%	0.57
2023	35.3%	42.1%	-0.14	35.3%	42.8%	-0.16
2024	-29.4%	17.5%	-0.37	-29.4%	17.9%	-0.38
**Vital signs**						
Body temperature	64.7%	51.3%	0.28	64.7%	52.3%	0.25
Diastolic blood pressure	64.7%	55.2%	0.20	64.7%	56.1%	0.18
Heart rate	64.7%	55.2%	0.20	64.7%	56.1%	0.18
Systolic blood pressure	64.7%	55.2%	0.20	64.7%	56.1%	0.18
Respiratory rate	52.9%	47.8%	0.10	52.9%	49.0%	0.08
**Oxygenation**						
Oxygen saturation in Arterial blood by Pulse oximetry	52.9%	41.6%	0.23	52.9%	42.6%	0.21
**Comorbidities**						
Acute kidney injury	-29.4%	10.6%	0.04	-29.4%	10.2%	0.05
Acute respiratory distress syndrome	-29.4%	1.8%	0.40	-29.4%	1.7%	0.41
Aplastic anemia	-29.4%	-1.1%	0.29	-29.4%	-1.2%	0.29
Chronic obstructive lung disease	-29.4%	5.3%	0.40	-29.4%	5.4%	0.39
Congestive heart failure	-29.4%	4.4%	0.07	-29.4%	4.5%	0.06
Cor pulmonale	-29.4%	-1.1%	0.33	-29.4%	-1.2%	0.33
Heart failure	-29.4%	6.9%	-0.04	-29.4%	7.1%	-0.05
Left heart failure	-29.4%	2.5%	0.17	-29.4%	2.6%	0.16
Pneumococcal pneumonia	-29.4%	-1.1%	0.29	-29.4%	-1.2%	0.29
Pneumonia	76.5%	57.5%	0.41	76.5%	58.5%	0.39
Pneumonia due to Klebsiella pneumoniae	-29.4%	1.4%	0.24	-29.4%	1.4%	0.24
Pneumonia due to Pseudomonas	-29.4%	1.4%	0.24	-29.4%	1.4%	0.24
Pulmonary edema	-29.4%	2.3%	0.18	-29.4%	2.4%	0.18
Pulmonary embolism	-29.4%	-1.1%	0.47	-29.4%	-1.2%	0.47
Renal failure syndrome	-29.4%	4.6%	0.26	-29.4%	4.3%	0.28
Respiratory failure	41.2%	15.4%	0.60	41.2%	15.7%	0.60
Septic shock	-29.4%	2.3%	0.38	-29.4%	2.4%	0.37
**Medications**						
Dexamethasone	47.1%	47.6%	-0.01	47.1%	48.4%	-0.03
Methylprednisolone	-29.4%	-1.1%	0.28	-29.4%	-1.2%	0.28
Prednisolone	-29.4%	2.8%	0.35	-29.4%	2.6%	0.36

ISH, International St. Mary's Hospital; PS, Propensity Score; Std. Diff, Standardized Difference.

Supplementary Table S15A. Baseline characteristics before and after propensity-score adjustment with expanded severity covariates: fluoroquinolones vs third-generation cephalosporins (JCMJ)

Characteristic	Before PS adjustment			After PS adjustment		
	Target (%)	Comparator (%)	Std. Diff	Target (%)	Comparator (%)	Std. Diff
**Age group**						
20-24	-2.8%	-4.6%	0.03	-5.1%	-4.7%	0.07
25-29	-2.8%	-4.6%	0.10	-5.2%	-4.7%	0.06
30-34	-2.8%	-4.6%	0.07	-5.2%	-4.7%	0.15
40-44	-2.8%	-4.6%	0.07	-5.2%	-4.7%	0.15
45-49	-2.8%	-4.6%	-0.17	-5.2%	-4.7%	-0.12
55-59	6.6%	5.5%	0.05	10.4%	6.2%	0.15
60-64	8.8%	11.9%	-0.10	11.5%	11.6%	-0.01
65-69	10.5%	10.1%	0.01	10.4%	10.9%	-0.02
70-74	9.4%	11.0%	-0.05	7.3%	10.2%	-0.10
75-79	18.2%	12.8%	0.15	13.5%	12.5%	0.03
85-89	11.6%	11.0%	0.02	8.3%	12.5%	-0.14
90-94	3.9%	-4.6%	0.06	-5.2%	-4.7%	-0.07
**Index Year**						
2015	2.8%	4.6%	-0.10	-5.2%	-4.7%	-0.08
2016	6.6%	4.6%	0.09	7.3%	-4.7%	0.11
2017	7.2%	6.4%	0.03	10.4%	7.3%	0.11
2018	5.0%	11.9%	-0.25	9.4%	9.7%	-0.01
2019	6.6%	10.1%	-0.13	6.2%	10.4%	-0.15
2021	3.9%	5.5%	-0.08	5.2%	5.0%	0.01
2022	14.4%	17.4%	-0.08	19.8%	18.7%	0.03
2023	19.3%	26.6%	-0.17	22.9%	26.4%	-0.08
**Laboratory**						
C-reactive protein	92.3%	91.7%	0.02	91.7%	92.2%	-0.02
**Vital signs**						
Body temperature	87.3%	84.4%	0.08	84.4%	87.0%	-0.07
Diastolic blood pressure	89.5%	88.1%	0.05	86.5%	90.3%	-0.12
Heart rate	89.5%	88.1%	0.05	86.5%	90.3%	-0.12
Systolic blood pressure	89.5%	88.1%	0.05	86.5%	90.3%	-0.12
Respiratory rate	79.0%	65.1%	0.31	70.8%	71.9%	-0.02
**Comorbidities**						
Acute exacerbation of chronic obstructive airways disease	-2.8%	-4.6%	0.10	-5.2%	-4.7%	0.08
Acute renal failure syndrome	8.3%	13.8%	-0.18	9.4%	14.1%	-0.15
Bacterial pneumonia	5.0%	5.5%	-0.02	7.3%	5.6%	0.07
Chronic obstructive lung disease	6.6%	-4.6%	0.18	5.2%	-4.7%	0.10
Heart failure	11.0%	9.2%	0.06	11.5%	9.9%	0.05
Hypertensive heart disease with congestive heart failure	-2.8%	-4.6%	-0.06	-5.2%	-4.7%	-0.08
Mild chronic obstructive pulmonary disease	-2.8%	-4.6%	0.07	-5.2%	-4.7%	0.08
Pneumonia	47.0%	33.9%	0.27	41.7%	37.8%	0.08
Pulmonary edema	3.9%	5.5%	-0.08	5.2%	5.7%	-0.02
Septic shock	4.4%	-4.6%	0.04	-5.2%	-4.7%	0.03
Viral pneumonia	-2.8%	-4.6%	-0.09	-5.2%	-4.7%	-0.20
**Medications**						
Dexamethasone	6.1%	-4.6%	0.11	-5.2%	-4.7%	-0.12
Methylprednisolone	9.4%	-4.6%	0.28	-5.2%	-4.7%	-0.07

JCMJ, Jecheon Myongji Hospital; PS, Propensity Score; Std. Diff, Standardized Difference.

Supplementary Table S15B. Baseline characteristics before and after propensity-score adjustment with expanded severity covariates: aminopenicillin/β-lactamase inhibitor combinations vs third-generation cephalosporins (JCMJ)

Characteristic	Before PS adjustment			After PS adjustment		
	Target (%)	Comparator	Std. Diff	Target (%)	Comparator	Std. Diff
**Age group**						
25-29	-17.2%	-4.6%	0.51	-17.2%	-3.4%	0.50
30-34	-17.2%	-4.6%	0.04	-17.2%	4.0%	-0.03
35-39	-17.2%	-4.6%	0.31	-17.2%	4.0%	0.25
40-44	-17.2%	-4.6%	0.31	-17.2%	-3.4%	0.26
45-49	-17.2%	-4.6%	0.26	-17.2%	5.2%	0.19
50-54	-17.2%	-4.6%	0.19	-17.2%	3.4%	0.16
55-59	-17.2%	5.5%	0.18	-17.2%	6.0%	0.16
60-64	-17.2%	11.9%	-0.32	-17.2%	14.6%	-0.40
70-74	20.7%	11.0%	0.27	20.7%	11.8%	0.24
75-79	-17.2%	12.8%	-0.20	-17.2%	9.7%	-0.10
80-84	-17.2%	18.3%	-0.35	-17.2%	18.9%	-0.36
**Index Year**						
2015	-17.2%	4.6%	-0.06	-17.2%	4.0%	-0.03
2016	-17.2%	4.6%	-0.06	-17.2%	5.4%	-0.10
2017	17.2%	6.4%	0.34	17.2%	4.6%	0.42
2018	20.7%	11.9%	0.24	20.7%	12.3%	0.23
2019	-17.2%	10.1%	-0.27	-17.2%	10.6%	-0.28
2020	17.2%	11.0%	0.18	17.2%	11.2%	0.17
2021	-17.2%	5.5%	-0.10	-17.2%	7.2%	-0.17
2022	-17.2%	17.4%	-0.21	-17.2%	20.4%	-0.28
2023	-17.2%	26.6%	-0.43	-17.2%	22.9%	-0.34
2024	-17.2%	-4.6%	0.17	-17.2%	-3.4%	0.24
**Laboratory**						
C-reactive protein	86.2%	91.7%	-0.18	86.2%	89.4%	-0.10
**Vital signs**						
Body temperature	82.8%	84.4%	-0.04	82.8%	85.1%	-0.06
Diastolic blood pressure	82.8%	88.1%	-0.15	82.8%	87.4%	-0.13
Heart rate	82.8%	88.1%	-0.15	82.8%	87.4%	-0.13
Systolic blood pressure	82.8%	88.1%	-0.15	82.8%	87.4%	-0.13
Respiratory rate	55.2%	65.1%	-0.21	55.2%	62.2%	-0.14
**Comorbidities**						
Acute hypercapnic respiratory failure	-17.2%	-4.6%	0.10	-17.2%	-3.4%	0.13
Ascites	-17.2%	-4.6%	0.10	-17.2%	-3.4%	0.07
Ascites due to alcoholic cirrhosis	-17.2%	-4.6%	0.10	-17.2%	-3.4%	0.07
Bacterial pneumonia	-17.2%	5.5%	0.06	-17.2%	4.3%	0.11
Bronchopneumonia	-17.2%	-4.6%	0.17	-17.2%	-3.4%	0.15
Chronic obstructive lung disease	-17.2%	-4.6%	0.04	-17.2%	-3.4%	0.18
Congestive heart failure	-17.2%	-4.6%	0.19	-17.2%	3.4%	0.16
Decompensated cirrhosis of liver	-17.2%	-4.6%	0.17	-17.2%	-3.4%	0.15
Heart failure	-17.2%	9.2%	-0.08	-17.2%	6.9%	0.00
Mild chronic obstructive pulmonary disease	-17.2%	-4.6%	0.17	-17.2%	-3.4%	0.24
Pulmonary edema	-17.2%	5.5%	-0.10	-17.2%	5.7%	-0.11
Shock	-17.2%	-4.6%	0.17	-17.2%	-3.4%	0.15
**Medications**						
Dexamethasone	-17.2%	-4.6%	-0.01	-17.2%	-3.4%	0.03
Methylprednisolone	-17.2%	-4.6%	0.04	-17.2%	-3.4%	0.18
Prednisolone	-17.2%	-4.6%	0.31	-17.2%	-3.4%	0.30

JCMJ, Jecheon Myongji Hospital; PS, Propensity Score; Std. Diff, Standardized Difference.

Supplementary Table S16A. Baseline characteristics before and after propensity-score adjustment with expanded severity covariates: fluoroquinolones vs third-generation cephalosporins (KDH)

Characteristic	Before PS adjustment			After PS adjustment		
	Target (%)	Comparator (%)	Std. Diff	Target (%)	Comparator (%)	Std. Diff
**Age group**						
35-39	-4.5%	-4.5%	-0.08	-6.3%	-4.4%	-0.05
40-44	-4.5%	-4.5%	-0.06	-6.3%	-4.4%	-0.01
45-49	4.5%	-4.5%	0.05	-6.3%	-4.4%	0.07
50-54	-4.5%	-4.5%	0.06	-6.6%	-3.6%	-0.09
55-59	4.5%	6.3%	-0.08	-6.3%	5.9%	-0.10
60-64	9.0%	8.1%	0.03	8.9%	9.1%	-0.01
65-69	8.1%	10.8%	-0.09	-6.3%	9.3%	-0.16
70-74	9.0%	7.2%	0.07	8.9%	7.2%	0.06
75-79	14.4%	13.5%	0.03	12.7%	13.1%	-0.01
80-84	17.1%	16.2%	0.02	17.7%	16.5%	0.03
85-89	17.1%	14.4%	0.07	20.3%	13.3%	0.19
90-94	4.5%	5.4%	-0.04	-6.3%	6.8%	-0.07
95-99	-4.5%	-4.5%	-0.06	-6.3%	-4.4%	-0.07
**Index Year**						
2021	12.6%	7.2%	0.18	16.5%	8.2%	0.25
2022	45.0%	55.0%	-0.20	39.2%	54.4%	-0.31
2024	13.5%	11.7%	0.05	12.7%	14.3%	-0.05
**Vital signs**						
Body temperature	98.2%	100.0%	-0.19	97.5%	100.0%	-0.23
Diastolic blood pressure	98.2%	100.0%	-0.19	97.5%	100.0%	-0.23
Heart rate	98.2%	100.0%	-0.19	97.5%	100.0%	-0.23
Systolic blood pressure	98.2%	100.0%	-0.19	97.5%	100.0%	-0.23
Respiratory rate	98.2%	100.0%	-0.19	97.5%	100.0%	-0.23
**Comorbidities**						
Acute respiratory failure	-4.5%	-4.5%	0.14	-6.3%	-4.4%	0.09
Ascites	-4.5%	-4.5%	-0.06	-6.3%	-4.4%	0.09
Ascites due to alcoholic cirrhosis	-4.5%	-4.5%	-0.14	-6.3%	-4.4%	-0.05
Chronic obstructive lung disease	5.4%	4.5%	0.04	6.3%	5.1%	0.06
Heart failure	5.4%	8.1%	-0.11	-6.3%	7.8%	-0.17
Hepatic failure	-4.5%	-4.5%	-0.08	-6.3%	-4.4%	-0.05
Hypertensive heart disease with congestive heart failure	-4.5%	-4.5%	-0.18	-6.3%	-4.4%	-0.15
Hypertensive renal failure	-4.5%	-4.5%	-0.18	-6.3%	-4.4%	-0.15
Moderate chronic obstructive pulmonary disease	-4.5%	-4.5%	0.14	-6.3%	-4.4%	0.18
Pneumonia	41.4%	27.9%	0.29	27.8%	35.9%	-0.17
Pulmonary embolism	-4.5%	-4.5%	-0.11	-6.3%	-4.4%	-0.05
Pulmonary emphysema	-4.5%	-4.5%	0.08	-6.3%	-4.4%	0.07
**Medications**						
Methylprednisolone	44.1%	22.5%	0.47	31.6%	29.1%	0.06
Prednisolone	-4.5%	-4.5%	-0.08	-6.3%	-4.4%	-0.09

KDH, Kangdong Sacred Heart Hospital; PS, Propensity Score; Std. Diff, Standardized Difference.

Supplementary Table S16B. Baseline characteristics before and after propensity-score adjustment with expanded severity covariates: aminopenicillin/β-lactamase inhibitor combinations vs third-generation cephalosporins (KDH)

Characteristic	Before PS adjustment			After PS adjustment		
	Target	Comparator	Std. Diff	Target (%)	Comparator	Std.
**Age group**						
20-24	-38.5%	-4.5%	0.5	-38.5%	-4.8%	0.46
25-29	-38.5%	-4.5%	0.28	-38.5%	-4.8%	0.27
30-34	-38.5%	-4.5%	0.34	-38.5%	-4.8%	0.23
35-39	-38.5%	-4.5%	0.28	-38.5%	-4.8%	0.23
50-54	-38.5%	-4.5%	0.28	-38.5%	-4.8%	0.34
55-59	-38.5%	6.3%	0.05	-38.5%	6.4%	0.05
65-69	-38.5%	10.8%	0.14	-38.5%	7.9%	0.23
75-79	-38.5%	13.5%	-0.19	-38.5%	14.3%	-0.21
80-84	-38.5%	16.2%	-0.27	-38.5%	16.6%	-0.27
**Index Year**						
2022	61.5%	55.0%	0.13	61.5%	60.1%	0.03
2023	-38.5%	26.1%	0.10	-38.5%	25.4%	0.12
2024	-38.5%	11.7%	-0.14	-38.5%	7.5%	0.01
**Laboratory**						
C-reactive protein	92.3%	97.3%	-0.23	92.3%	91.0%	0.05
**Comorbidities**						
Heart failure	-38.5%	8.1%	-0.02	-38.5%	-4.8%	0.36
Pneumococcal pneumonia	-38.5%	-4.5%	0.34	-38.5%	-4.8%	0.36
Pneumonia	-38.5%	27.9%	-0.31	-38.5%	17.5%	-0.06
**Medications**						
Dexamethasone	-38.5%	22.5%	-0.42	-38.5%	12.0%	-0.15
Methylprednisolone	-38.5%	-4.5%	0.34	-38.5%	-4.8%	0.36

KDH, Kangdong Sacred Heart Hospital; PS, Propensity Score; Std. Diff, Standardized Difference.

Supplementary Table S17A. Baseline characteristics before and after propensity-score adjustment with expanded severity covariates: fluoroquinolones vs third-generation cephalosporins (KHMC)

Characteristic	Before PS adjustment			After PS adjustment		
	Target (%)	Comparator (%)	Std. Diff	Target (%)	Comparator (%)	Std. Diff
**Age group**						
20-24	-7.2%	-3.1%	0.08	-8.2%	-2.0%	0.13
35-39	-7.2%	-3.1%	-0.03	-8.2%	-2.1%	0.06
40-44	-7.2%	-3.1%	0.02	-8.2%	2.5%	0.05
45-49	-7.2%	3.1%	0.13	-8.2%	3.7%	0.13
50-54	-7.2%	3.1%	-0.11	-8.2%	-2.0%	0.00
55-59	-7.2%	6.3%	-0.02	-8.2%	9.9%	-0.19
60-64	10.1%	6.9%	0.12	9.8%	4.9%	0.19
65-69	-7.2%	11.3%	-0.33	-8.2%	8.8%	-0.23
70-74	8.7%	13.8%	-0.16	9.8%	14.2%	-0.13
75-79	18.8%	17.0%	0.05	14.8%	18.9%	-0.11
80-84	18.8%	16.4%	0.07	18.0%	18.8%	-0.02
85-89	17.4%	13.8%	0.10	19.7%	12.2%	0.20
90-94	-7.2%	-3.1%	-0.08	-8.2%	-2.0%	0.00
**Index Year**						
2021	-7.2%	19.5%	-0.42	-8.2%	7.4%	-0.03
2022	20.3%	39.0%	-0.42	21.3%	19.4%	0.05
2023	46.4%	30.2%	0.34	52.5%	51.6%	0.02
**Laboratory**						
C-reactive protein	98.6%	100.0%	-0.17	98.4%	100.0%	-0.18
**Comorbidities**						
Acidosis	-7.2%	3.1%	-0.11	-8.2%	2.6%	-0.07
Acute renal failure syndrome	11.6%	17.0%	-0.15	13.1%	15.9%	-0.08
Bacterial pneumonia	-7.2%	-3.1%	0.02	-8.2%	2.5%	-0.06
Bronchopneumonia	-7.2%	-3.1%	-0.08	-8.2%	4.5%	-0.17
Chronic obstructive lung disease	7.2%	5.7%	0.07	-8.2%	2.8%	0.11
Chronic renal failure	-7.2%	3.1%	-0.11	-8.2%	3.1%	-0.09
End stage renal disease on dialysis due to type 2 diabetes mellitus	-7.2%	-3.1%	0.02	-8.2%	-2.0%	0.16
End-stage renal disease	-7.2%	-3.1%	-0.08	-8.2%	-2.1%	0.03
Heart failure	-7.2%	12.6%	-0.30	-8.2%	9.4%	-0.18
Hepatic failure	-7.2%	3.8%	-0.15	-8.2%	3.5%	-0.12
Hypertensive heart disease with congestive heart failure	-7.2%	6.3%	-0.25	-8.2%	5.8%	-0.22
Hypertensive renal failure	-7.2%	-3.1%	0.17	-8.2%	-2.0%	0.17
Localized edema	-7.2%	-3.1%	0.19	-8.2%	2.0%	0.09
Moderate chronic obstructive pulmonary disease	-7.2%	3.1%	0.13	-8.2%	-2.0%	0.09
Pneumonia	39.1%	30.8%	0.18	31.1%	29.3%	0.04
Pulmonary embolism	-7.2%	-3.1%	0.02	-8.2%	-2.0%	0.15
Severe chronic obstructive pulmonary disease	-7.2%	-3.1%	0.08	-8.2%	-2.0%	0.15
Thrombophlebitis of deep veins of lower extremity	-7.2%	-3.1%	-0.03	-8.2%	-2.0%	0.08
**Medications**						
Dexamethasone	7.2%	27.0%	-0.54	8.2%	9.3%	-0.04
Methylprednisolone	-7.2%	-3.1%	0.14	-8.2%	-2.0%	0.18
Prednisolone	-7.2%	5.0%	-0.03	-8.2%	4.3%	0.03

KHMC, Kyunghee University Medical Center; PS, Propensity Score; Std. Diff, Standardized Difference.

Supplementary Table S17B. Baseline characteristics before and after propensity-score adjustment with expanded severity covariates: aminopenicillin/β-lactamase inhibitor combinations vs third-generation cephalosporins (KHMC)

Characteristic	Before PS adjustment			After PS adjustment		
	Target (%)	Comparator (%)	Std. Diff	Target (%)	Comparator (%)	Std. Diff
**Age group**						
20-24	-18.5%	-3.1%	0.46	-33.3%	-2.5%	0.27
45-49	-18.5%	3.1%	0.19	-33.3%	4.1%	0.33
50-54	-18.5%	3.1%	0.03	-33.3%	3.6%	0.14
55-59	-18.5%	6.3%	-0.12	-33.3%	6.7%	0.00
60-64	29.6%	6.9%	0.62	-33.3%	12.9%	0.01
70-74	-18.5%	13.8%	-0.36	-33.3%	12.0%	-0.18
75-79	-18.5%	17.0%	-0.06	-33.3%	13.6%	0.33
80-84	-18.5%	16.4%	-0.43	-33.3%	10.4%	-0.13
85-89	-18.5%	13.8%	-0.36	-33.3%	10.9%	-0.15
**Index Year**						
2022	63.0%	39.0%	0.49	53.3%	36.2%	0.35
2023	25.9%	30.2%	-0.10	33.3%	39.4%	-0.13
2024	-18.5%	10.1%	0.03	-33.3%	19.1%	-0.16
**Vital signs**						
Body temperature	100.0%	98.7%	0.16	100.0%	98.4%	0.18
Diastolic blood pressure	100.0%	99.4%	0.11	100.0%	99.7%	0.07
Heart rate	100.0%	99.4%	0.11	100.0%	99.7%	0.07
Systolic blood pressure	100.0%	99.4%	0.11	100.0%	99.7%	0.07
Respiratory rate	100.0%	98.7%	0.16	100.0%	98.4%	0.18
**Comorbidities**						
Acute renal failure due to tubular necrosis	-18.5%	-3.1%	0.21	-33.3%	-2.5%	0.35
Acute renal failure syndrome	-18.5%	17.0%	-0.45	-33.3%	7.8%	-0.04
Chronic renal failure	-18.5%	3.1%	0.03	-33.3%	2.8%	0.18
End-stage renal disease	-18.5%	-3.1%	0.07	-33.3%	-2.5%	0.33
Heart failure	-18.5%	12.6%	-0.17	-33.3%	7.0%	0.21
Hypertensive heart disease with congestive heart failure	-18.5%	6.3%	-0.12	-33.3%	-2.5%	0.25

KHMC, Kyunghee University Medical Center; PS, Propensity Score; Std. Diff, Standardized Difference.

Supplementary Table S18A. Baseline characteristics before and after propensity-score adjustment with expanded severity covariates: fluoroquinolones vs third-generation cephalosporins (KWMC)

Characteristic	Before PS adjustment			After PS adjustment		
	Target (%)	Comparator (%)	Std. Diff	Target (%)	Comparator (%)	Std. Diff
**Age group**						
20-24	-2.2%	-2.0%	-0.03	-4.0%	-1.7%	0.07
30-34	-2.2%	2.0%	-0.05	-4.0%	-1.7%	-0.03
40-44	-2.2%	-2.0%	-0.02	-4.0%	-1.7%	-0.02
45-49	-2.2%	-2.0%	0.01	-4.0%	1.8%	0.04
50-54	2.7%	3.2%	-0.03	-4.0%	2.5%	-0.01
55-59	4.0%	4.4%	-0.02	4.0%	3.1%	0.05
60-64	3.1%	3.2%	-0.01	4.0%	3.6%	0.02
65-69	8.0%	6.4%	0.06	9.5%	6.9%	0.10
70-74	8.4%	7.2%	0.05	7.1%	7.9%	-0.03
75-79	13.3%	15.5%	-0.06	15.1%	15.1%	0.00
80-84	22.1%	21.1%	0.02	19.0%	25.8%	-0.16
85-89	16.4%	15.5%	0.02	14.3%	13.8%	0.01
90-94	11.5%	9.6%	0.06	13.5%	10.0%	0.11
95-99	4.4%	2.8%	0.09	5.6%	3.3%	0.11
**Index Year**						
2020	-2.2%	-2.0%	0.13	-4.0%	-1.7%	0.13
2021	10.6%	2.0%	0.36	4.0%	2.4%	0.09
2022	53.5%	43.4%	0.20	45.2%	47.2%	-0.04
2023	34.1%	54.2%	-0.41	48.4%	49.6%	-0.02
**Vital signs**						
Body temperature	96.9%	100.0%	-0.25	99.2%	100.0%	-0.13
Diastolic blood pressure	96.9%	100.0%	-0.25	99.2%	100.0%	-0.13
Heart rate	96.9%	100.0%	-0.25	99.2%	100.0%	-0.13
Systolic blood pressure	96.9%	100.0%	-0.25	99.2%	100.0%	-0.13
Respiratory rate	96.9%	100.0%	-0.25	99.2%	100.0%	-0.13
**Comorbidities**						
Acidosis	3.5%	2.0%	0.09	-4.0%	-2.5%	0.04
Acute exacerbation of chronic obstructive pulmonary disease	-2.2%	-2.0%	0.01	-4.0%	-1.7%	0.07
Acute kidney injury	23.0%	19.9%	0.08	20.6%	22.3%	-0.04
Acute pulmonary edema	-2.2%	-2.0%	-0.05	-4.0%	-1.7%	0.02
Acute respiratory distress syndrome	2.7%	-2.0%	0.19	-4.0%	-1.7%	0.07
Agranulocytosis	-2.2%	-2.0%	0.05	-4.0%	-1.7%	0.13
Ascites	-2.2%	-2.0%	-0.06	-4.0%	-1.7%	-0.04
Ascites due to alcoholic cirrhosis	-2.2%	-2.0%	-0.03	-4.0%	-1.7%	0.08
Bacterial pneumonia	2.2%	2.0%	0.02	-4.0%	-1.7%	0.01
Chronic obstructive pulmonary disease	4.4%	4.8%	-0.02	4.0%	4.9%	-0.04
Chronic obstructive pulmonary disease with acute lower respiratory infection	-2.2%	-2.0%	-0.06	-4.0%	1.9%	-0.02
Congestive heart failure	2.2%	-2.0%	0.12	-4.0%	-1.7%	0.06
Diastolic heart failure	3.1%	-2.0%	0.13	-4.0%	2.0%	0.03
End stage renal disease on dialysis due to type 2 diabetes mellitus	-2.2%	-2.0%	-0.05	-4.0%	-1.7%	-0.04
Heart failure	12.8%	12.0%	0.03	15.1%	14.3%	0.02
Hypertensive heart disease with congestive heart failure	-2.2%	-2.0%	0.10	-4.0%	-1.7%	0.12
Hypovolemic shock	-2.2%	-2.0%	-0.05	-4.0%	-1.7%	-0.02
Left heart failure	3.1%	2.8%	0.02	4.0%	3.6%	0.02
Moderate chronic obstructive pulmonary disease	4.0%	2.8%	0.07	4.0%	3.8%	0.01
Pneumonia	63.7%	40.2%	0.48	59.5%	61.9%	-0.05
Pulmonary edema	2.7%	3.6%	-0.05	-4.0%	4.6%	-0.12
Pulmonary embolism	2.2%	-2.0%	0.16	-4.0%	-1.7%	0.07
Renal failure syndrome	-2.2%	2.4%	-0.04	-4.0%	2.6%	-0.14
Septic shock	6.6%	-2.0%	0.31	4.0%	-1.7%	0.20
Severe chronic obstructive pulmonary disease	-2.2%	-2.0%	0.01	-4.0%	1.8%	0.04
Shock	-2.2%	-2.0%	0.10	-4.0%	-1.7%	0.07
Thrombophlebitis of deep veins of lower extremity	2.2%	2.0%	0.02	-4.0%	2.9%	-0.03
Viral pneumonia	25.2%	10.4%	0.40	19.0%	16.8%	0.06
White blood cell disorder	-2.2%	-2.0%	0.01	-4.0%	-1.7%	0.09
**Medications**						
Dexamethasone	11.1%	5.6%	0.2	12.7%	9.5%	0.10
Hydrocortisone	-2.2%	-2.0%	0.01	-4.0%	-1.7%	0.13
Methylprednisolone	4.4%	2.0%	0.14	6.3%	2.5%	0.19
Prednisolone	-2.2%	-2.0%	0.05	-4.0%	-1.7%	-0.04

KWMC, Gangwon National University Medical Center; PS, Propensity Score; Std. Diff, Standardized Difference.

Supplementary Table S18B. Baseline characteristics before and after propensity-score adjustment with expanded severity covariates: aminopenicillin/β-lactamase inhibitor combinations vs third-generation cephalosporins (KWMC)

Characteristic	Before PS adjustment			After PS adjustment		
	Target (%)	Comparator	Std. Diff	Target	Comparator	Std.
**Age group**						
15-19	-15.6%	-2.0%	0.30	-15.6%	-0.9%	0.34
20-24	-15.6%	-2.0%	0.27	-15.6%	3.4%	0.13
25-29	-15.6%	2.0%	0.07	-15.6%	5.1%	-0.10
35-39	-15.6%	2.0%	0.07	-15.6%	3.5%	-0.02
40-44	-15.6%	-2.0%	0.44	-15.6%	3.4%	0.34
45-49	-15.6%	-2.0%	0.13	-15.6%	-0.9%	0.23
50-54	-15.6%	3.2%	0.00	-15.6%	9.9%	-0.27
55-59	-15.6%	4.4%	0.30	-15.6%	8.0%	0.15
60-64	-15.6%	3.2%	0.00	-15.6%	2.5%	0.04
65-69	-15.6%	6.4%	0.11	-15.6%	6.4%	0.11
70-74	-15.6%	7.2%	0.08	-15.6%	4.9%	0.18
75-79	-15.6%	15.5%	-0.30	-15.6%	12.0%	-0.20
80-84	-15.6%	21.1%	-0.57	-15.6%	11.7%	-0.33
85-89	-15.6%	15.5%	-0.19	-15.6%	12.7%	-0.10
90-94	-15.6%	9.6%	-0.12	-15.6%	8.3%	-0.08
95-99	-15.6%	2.8%	0.02	-15.6%	2.5%	0.04
**Index Year**						
2022	56.2%	43.4%	0.26	56.2%	49.5%	0.14
2023	43.8%	54.2%	-0.21	43.8%	46.7%	-0.06
**Comorbidities**						
Diastolic heart failure	-15.6%	-2.0%	0.13	-15.6%	1.7%	0.10
Heart failure	-15.6%	12.0%	-0.20	-15.6%	12.5%	-0.22
Mild chronic obstructive pulmonary disease	-15.6%	-2.0%	0.10	-15.6%	-0.9%	0.19
Pneumonia	-15.6%	40.2%	-0.66	-15.6%	17.6%	-0.14
Viral pneumonia	-15.6%	10.4%	-0.29	-15.6%	2.7%	0.02
**Medications**						
Dexamethasone	-15.6%	6.4%	0.11	-15.6%	1.9%	0.33
Prednisolone	-15.6%	-2.0%	0.27	-15.6%	-0.9%	0.33

KWMC, Gangwon National University Medical Center; PS, Propensity Score; Std. Diff, Standardized Difference.

Supplementary Table S19A. Baseline characteristics before and after propensity-score adjustment with expanded severity covariates: fluoroquinolones vs third-generation cephalosporins (MJH)

Characteristic	Before PS adjustment			After PS adjustment		
	Target (%)	Comparator (%)	Std. Diff	Target (%)	Comparator (%)	Std. Diff
**Age group**						
20-24	-2.5%	-2.5%	0.06	-3.9%	-1.7%	0.12
25-29	-2.5%	-2.5%	-0.06	-3.9%	-1.7%	0.11
40-44	-2.5%	-2.5%	-0.06	-3.9%	-1.7%	0.09
45-49	-2.5%	-2.5%	-0.10	-3.9%	2.3%	-0.13
50-54	-2.5%	2.9%	-0.14	-3.9%	3.1%	-0.10
55-59	3.4%	3.9%	-0.03	4.7%	4.1%	0.03
60-64	4.4%	5.4%	-0.05	-3.9%	4.3%	-0.02
70-74	8.3%	5.9%	0.10	10.2%	6.2%	0.14
75-79	15.2%	14.7%	0.01	13.3%	13.6%	-0.01
80-84	19.6%	22.5%	-0.07	18.8%	23.8%	-0.12
85-89	22.1%	17.6%	0.11	19.5%	18.1%	0.04
90-94	9.8%	10.8%	-0.03	10.9%	12.9%	-0.06
95-99	5.9%	2.9%	0.14	6.2%	3.8%	0.11
**Index Year**						
2020	-2.5%	3.9%	-0.12	-3.9%	3.9%	-0.14
2022	36.3%	38.2%	-0.04	35.9%	38.9%	-0.06
2023	35.8%	38.2%	-0.05	40.6%	36.4%	0.09
2024	20.1%	13.7%	0.17	16.4%	15.0%	0.04
**Oxygenation**						
Oxygen saturation in Arterial blood by Pulse oximetry	51.5%	31.4%	0.42	43.0%	45.2%	-0.05
**Comorbidities**						
Acidosis	-2.5%	-2.5%	0.06	-3.9%	-1.7%	0.07
Acute exacerbation of chronic obstructive airways disease	-2.5%	-2.5%	0.13	-3.9%	-1.7%	0.05
Acute renal failure syndrome	12.7%	10.8%	0.06	12.5%	10.6%	0.06
Ascites	-2.5%	-2.5%	-0.06	-3.9%	-1.7%	-0.07
Bacterial pneumonia	2.5%	-2.5%	0.11	-3.9%	-1.7%	0.06
Bronchopneumonia	-2.5%	-2.5%	0.06	-3.9%	-1.7%	0.05
Chronic obstructive lung disease	4.9%	4.4%	0.02	-3.9%	5.7%	-0.13
Congestive heart failure	7.8%	-2.5%	0.28	4.7%	3.1%	0.08
Diastolic heart failure	-2.5%	-2.5%	0.05	-3.9%	-1.7%	0.06
Heart failure	17.6%	14.7%	0.08	11.7%	17.8%	-0.17
Hepatic failure	2.5%	2.9%	-0.03	-3.9%	3.2%	-0.11
Hypertensive heart disease with congestive heart failure	3.4%	2.5%	0.06	3.9%	3.4%	0.03
Left heart failure	14.7%	15.2%	-0.01	12.5%	16.3%	-0.11
Moderate chronic obstructive pulmonary disease	-2.5%	-2.5%	0.08	-3.9%	-1.7%	0.13
Pneumonia	37.7%	19.6%	0.41	22.7%	29.7%	-0.16
Pulmonary emphysema	-2.5%	-2.5%	0.13	-3.9%	-1.7%	0.07
Renal failure syndrome	-2.5%	-2.5%	0.05	-3.9%	-1.7%	0.09
Right ventricular failure	-2.5%	-2.5%	0.05	-3.9%	-1.7%	0.07
Septic shock	5.9%	-2.5%	0.27	-3.9%	-1.7%	0.10
Severe chronic obstructive pulmonary disease	2.9%	-2.5%	0.10	-3.9%	-1.7%	0.01
**Medications**						
deflazacort	-2.5%	-2.5%	0.06	-3.9%	-1.7%	0.12
Hydrocortisone	-2.5%	-2.5%	-0.06	-3.9%	-1.7%	0.02
Methotrexate	-2.5%	-2.5%	0.10	-3.9%	-1.7%	0.12
Methylprednisolone	6.4%	4.4%	0.09	4.7%	5.0%	-0.01
Prednisolone	2.9%	2.5%	0.03	-3.9%	3.1%	-0.05

MJH, Myongji Hospital; PS, Propensity Score; Std. Diff, Standardized Difference.

Supplementary Table S19B. Baseline characteristics before and after propensity-score adjustment with expanded severity covariates: aminopenicillin/β-lactamase inhibitor combinations vs third-generation cephalosporins (MJH)

Characteristic	Before PS adjustment			After PS adjustment		
	Target	Comparator	Std. Diff	Target (%)	Comparator	Std.
**Age group**						
60-64	-45.5%	5.4%	0.41	-45.5%	5.4%	0.40
65-69	-45.5%	7.4%	0.33	-45.5%	7.3%	0.33
70-74	-45.5%	5.9%	0.39	-45.5%	5.9%	0.38
80-84	-45.5%	22.5%	-0.38	-45.5%	22.5%	-0.37
90-94	-45.5%	10.8%	-0.06	-45.5%	10.7%	-0.06
**Index Year**						
2021	54.5%	5.4%	1.27	54.5%	5.4%	1.27
2022	-45.5%	38.2%	-0.04	-45.5%	38.2%	-0.04
2024	-45.5%	13.7%	-0.15	-45.5%	13.9%	-0.15
**Oxygenation**						
Oxygen saturation in Arterial blood by Pulse oximetry	-45.5%-	31.4%	-0.58	-45.0%	31.7%	-0.35
**Comorbidities**						
Acute renal failure syndrome	-45.5%	10.8%	-0.06	-45.5%	10.7%	-0.06
Acute respiratory failure	-45.5%	-2.5%	0.41	-45.5%	-2.4%	0.41
Bacterial pneumonia	-45.5%	-2.5%	0.38	-45.5%	-2.4%	0.38
Congestive heart failure	-45.5%	-2.5%	0.32	-45.5%	-2.4%	0.31
Left heart failure	-45.5%	15.2%	0.08	-45.5%	15.1%	0.08
Renal failure syndrome	-45.5%	-2.5%	0.38	-45.5%	-2.4%	0.38
Viral pneumonia	45.5%	13.2%	0.76	45.5%	13.2%	0.76
**Medications**						
Dexamethasone	54.5%	20.1%	0.76	54.5%	20.0%	0.76
Prednisolone	-45.5%	2.5%	0.29	-45.5%	2.5%	0.29

MJH, Myongji Hospital; PS, Propensity Score; Std. Diff, Standardized Difference.

Supplementary Table S20A. Baseline characteristics before and after propensity-score adjustment with expanded severity covariates: fluoroquinolones vs third-generation cephalosporins (SCHBC)

Characteristic	Before PS adjustment			After PS adjustment		
	Target (%)	Comparator (%)	Std. Diff	Target (%)	Comparator (%)	Std. Diff
**Age group**						
25-29	-11.4%	-3.2%	0.07	-11.6%	-3.2%	0.07
35-39	-11.4%	-3.2%	0.15	-11.6%	-3.2%	0.15
40-44	-11.4%	3.9%	0.03	-11.6%	3.9%	0.04
45-49	-11.4%	3.9%	-0.09	-11.6%	3.5%	-0.07
50-54	-11.4%	4.5%	-0.12	-11.6%	4.7%	-0.13
55-59	11.4%	5.2%	0.23	11.6%	5.0%	0.24
60-64	-11.4%	16.8%	-0.51	-11.6%	17.4%	-0.52
65-69	15.9%	13.5%	0.07	14.0%	13.4%	0.02
70-74	-11.4%	7.7%	0.05	-11.6%	7.8%	0.06
75-79	-11.4%	8.4%	0.03	-11.6%	8.1%	0.04
85-89	15.9%	12.3%	0.11	16.3%	12.6%	0.11
90-94	-11.4%	4.5%	0.18	-11.6%	4.7%	0.18
**Index Year**						
2021	-11.4%	7.1%	-0.01	-11.6%	7.4%	-0.11
2022	79.5%	66.5%	0.30	81.4%	66.7%	0.34
2023	13.6%	22.6%	-0.23	14.0%	22.3%	-0.22
**Laboratory**						
C-reactive protein	95.5%	97.4%	-0.11	95.3%	97.7%	-0.13
**Oxygenation**						
Oxygen saturation in Arterial blood by Pulse oximetry	97.7%	96.8%	0.06	97.7%	96.7%	0.06
**Comorbidities**						
Acidosis	-11.4%	-3.2%	0.02	-11.6%	-3.2%	0.04
Acute exacerbation of chronic obstructive airways disease	-11.4%	-3.2%	-0.02	-11.6%	-3.2%	-0.01
Acute hypoxemic respiratory failure	-11.4%	-3.2%	0.28	-11.6%	-3.2%	0.29
Acute renal failure syndrome	27.3%	20.6%	0.16	27.9%	20.7%	0.17
Acute respiratory failure	11.4%	6.5%	0.17	11.6%	6.4%	0.18
Agranulocytosis	-11.4%	-3.2%	0.07	-11.6%	-3.2%	0.07
Ascites	-11.4%	6.5%	-0.21	-11.6%	6.8%	-0.22
Ascites due to alcoholic cirrhosis	-11.4%	5.2%	-0.15	-11.6%	5.2%	-0.15
Chronic obstructive lung disease	-11.4%	7.7%	-0.04	-11.6%	8.1%	-0.04
Congestive heart failure	15.9%	3.2%	0.44	16.3%	-3.2%	0.46
Disseminated intravascular coagulation	-11.4%	-3.2%	0.24	-11.6%	-3.2%	0.23
End stage renal disease on dialysis due to type 2 diabetes mellitus	-11.4%	7.1%	-0.01	-11.6%	7.4%	-0.02
Heart failure	-11.4%	-3.2%	0.28	-11.6%	-3.2%	0.28
Hepatic failure	-11.4%	5.8%	-0.18	-11.6%	5.8%	-0.18
Hepatorenal syndrome	-11.4%	-3.2%	0.07	-11.6%	-3.2%	0.09
Hypertensive renal failure	-11.4%	-3.2%	0.19	-11.6%	-3.2%	0.19
Mild chronic obstructive pulmonary disease	-11.4%	-3.2%	0.33	-11.6%	-3.2%	0.33
Pneumonia	45.5%	34.8%	0.22	44.2%	34.1%	0.21
Pulmonary edema	-11.4%	-3.2%	0.40	-11.6%	-3.2%	0.40
Pulmonary embolism	-11.4%	-3.2%	0.07	-11.6%	-3.2%	0.07
Pulmonary emphysema	-11.4%	3.2%	-0.06	-11.6%	3.3%	-0.06
Septic shock	-11.4%	-3.2%	0.28	-11.6%	-3.2%	0.28
Systolic heart failure	-11.4%	-3.2%	0.25	-11.6%	-3.2%	0.24
Thrombophlebitis of deep veins of lower extremity	-11.4%	9.0%	-0.18	-11.6%	9.7%	-0.20
Viral pneumonia	18.2%	17.4%	0.02	18.6%	15.9%	0.07
**Medications**						
Dexamethasone	11.4%	24.5%	-0.35	11.6%	24.4%	-0.34
Methylprednisolone	-11.4%	5.2%	0.15	-11.6%	5.2%	0.07
Prednisolone	-11.4%	3.9%	0.03	-11.6%	4.3%	0.02

SCHBC, Soonchunhyang University Hospital Bucheon Center; PS, Propensity Score; Std. Diff, Standardized Difference.

Supplementary Table S21A. Baseline characteristics before and after propensity-score adjustment with expanded severity covariates: fluoroquinolones vs third-generation cephalosporins (SCHCA)

Characteristic	Before PS adjustment			After PS adjustment		
	Target (%)	Comparator (%)	Std. Diff	Target (%)	Comparator (%)	Std. Diff
**Age group**						
20-24	-8.8%	-2.7%	0.01	-9.8%	-2.4%	0.04
25-29	-8.8%	-2.7%	0.31	-9.8%	-2.4%	0.34
35-39	-8.8%	-2.7%	0.12	-9.8%	-2.4%	0.18
45-49	-8.8%	2.7%	0.05	-9.8%	2.5%	0.08
50-54	-8.8%	7.5%	-0.18	-9.8%	5.7%	-0.08
55-59	-8.8%	7.0%	-0.26	-9.8%	9.2%	-0.32
60-64	-8.8%	8.6%	-0.13	-9.8%	6.6%	-0.03
65-69	15.8%	10.2%	0.17	11.8%	13.1%	-0.04
70-74	10.5%	8.6%	0.07	11.8%	8.4%	0.11
75-79	12.3%	12.3%	0.00	11.8%	13.3%	-0.05
80-84	21.1%	19.8%	0.03	19.6%	20.4%	-0.02
85-89	-8.8%	10.7%	-0.13	-9.8%	6.6%	0.05
90-94	-8.8%	4.3%	0.12	-9.8%	5.1%	0.11
**Index Year**						
2021	8.8%	11.2%	-0.08	-9.8%	8.7%	-0.03
2022	78.9%	75.9%	0.07	82.4%	77.5%	0.12
2023	10.5%	12.3%	-0.06	-9.8%	11.8%	-0.13
**Vital signs**						
Body temperature	100.0%	99.5%	0.10	100.0%	99.3%	0.11
**Laboratory**						
C-reactive protein	96.5%	100.0%	-0.27	96.1%	100.0%	-0.29
**Oxygenation**						
Oxygen saturation in Arterial blood by Pulse oximetry	96.5%	95.7%	0.04	96.1%	96.2%	-0.01
**Comorbidities**						
Acute exacerbation of chronic obstructive airways disease	-8.8%	-2.7%	0.29	-9.8%	-2.4%	0.34
Acute hypoxemic respiratory failure	19.3%	5.9%	0.41	11.8%	13.9%	-0.06
Acute pulmonary edema	-8.8%	-2.7%	0.06	-9.8%	-2.4%	0.04
Acute renal failure syndrome	28.1%	14.4%	0.34	27.5%	29.4%	-0.04
Acute respiratory distress syndrome	-8.8%	-2.7%	0.08	-9.8%	3.9%	-0.12
Acute respiratory failure	-8.8%	-2.7%	0.06	-9.8%	-2.4%	0.17
Ascites	-8.8%	-2.7%	-0.03	-9.8%	2.6%	-0.04
Chronic obstructive lung disease	-8.8%	4.8%	0.09	-9.8%	3.3%	0.12
Congestive heart failure	10.5%	6.4%	0.15	11.8%	10.7%	0.04
Diastolic heart failure	-8.8%	-2.7%	0.12	-9.8%	-2.4%	0.18
End stage renal disease on dialysis due to type 2 diabetes mellitus	-8.8%	2.7%	0.05	-9.8%	3.6%	0.02
Heart failure	-8.8%	-2.7%	-0.03	-9.8%	3.1%	-0.07
Hypertensive heart disease with congestive heart failure	-8.8%	-2.7%	0.21	-9.8%	-2.4%	0.27
Hypertensive renal failure	-8.8%	2.7%	-0.06	-9.8%	2.8%	-0.05
Hypovolemic shock	-8.8%	-2.7%	0.12	-9.8%	-2.4%	0.13
Ketoacidosis due to type 2 diabetes mellitus	-8.8%	-2.7%	0.12	-9.8%	-2.4%	0.13
Moderate chronic obstructive pulmonary disease	-8.8%	-2.7%	0.08	-9.8%	2.6%	0.07
Pneumonia	45.6%	41.2%	0.09	41.2%	42.7%	-0.03
Pneumonia due to Pseudomonas	-8.8%	-2.7%	0.21	-9.8%	-2.4%	0.13
Pulmonary edema	-8.8%	2.7%	0.05	-9.8%	3.4%	0.03
Pulmonary emphysema	-8.8%	-2.7%	0.24	-9.8%	-2.4%	0.27
Renal failure syndrome	-8.8%	4.3%	-0.04	-9.8%	4.3%	-0.02
Septic shock	-8.8%	2.7%	0.20	-9.8%	-2.4%	0.19
Severe chronic obstructive pulmonary disease	-8.8%	-2.7%	0.08	-9.8%	-2.4%	0.14
Systolic heart failure	-8.8%	-2.7%	0.27	-9.8%	-2.4%	0.29
Viral pneumonia	-8.8%	9.6%	-0.25	-9.8%	6.6%	-0.23
**Medications**						
Dexamethasone	17.5%	31.0%	-0.32	15.7%	17.6%	-0.05
Hydrocortisone	-8.8%	-2.7%	0.12	-9.8%	-2.4%	0.13
Methylprednisolone	-8.8%	2.7%	0.20	-9.8%	-2.4%	0.19
Prednisolone	-8.8%	-2.7%	0.01	-9.8%	3.4%	-0.09

SCHCA, Soonchunhyang University Hospital Cheonan Center; PS, Propensity Score; Std. Diff, Standardized Difference.

Supplementary Table S22A. Baseline characteristics before and after propensity-score adjustment with expanded severity covariates: fluoroquinolones vs third-generation cephalosporins (WKUH)

Characteristic	Before PS adjustment			After PS adjustment		
	Target (%)	Comparator (%)	Std. Diff	Target (%)	Comparator (%)	Std. Diff
**Age group**						
30-34	-2.7%	-2.1%	0.00	-4.2%	-2.1%	-0.03
40-44	-2.7%	-2.1%	0.03	-4.2%	-2.1%	0.06
45-49	-2.7%	3.8%	-0.13	-4.2%	3.3%	-0.05
50-54	-2.7%	2.5%	-0.02	-4.2%	-2.1%	0.10
55-59	4.9%	5.8%	-0.04	6.8%	5.3%	0.06
65-69	8.7%	8.3%	0.01	8.5%	8.8%	-0.01
70-74	10.3%	8.3%	0.07	9.3%	8.1%	0.05
75-79	9.2%	10.0%	-0.03	5.9%	9.6%	-0.14
80-84	15.2%	12.5%	0.08	16.1%	14.5%	0.04
85-89	20.1%	17.1%	0.08	21.2%	17.2%	0.10
90-94	14.7%	14.6%	0.00	14.4%	16.4%	0.06
**Index Year**						
2020	-2.7%	-2.1%	0.07	-4.3%	-2.1%	-0.07
2022	53.8%	49.2%	0.09	50.4%	44.0%	0.13
2023	39.1%	45.0%	-0.12	43.6%	48.7%	-0.10
2024	4.9%	3.8%	0.06	5.1%	5.0%	0.01
**Vital signs**						
Body temperature	93.5%	92.1%	0.05	90.7%	92.8%	-0.08
Diastolic blood pressure	94.6%	92.5%	0.08	92.4%	93.1%	-0.03
Heart rate	70.6%	91.7%	-0.56	70.6%	91.1%	-0.54
Systolic blood pressure	94.6%	92.5%	0.08	92.4%	93.1%	-0.03
Respiratory rate	94.0%	91.7%	0.09	91.5%	92.5%	-0.04
**Laboratory**						
C-reactive protein	99.5%	99.2%	0.04	99.2%	99.5%	-0.04
**Oxygenation**						
Oxygen saturation in Arterial blood by Pulse oximetry	94.0%	91.7%	0.09	91.5%	92.5%	-0.04
**Comorbidities**						
Acidosis	8.7%	4.6%	0.17	6.8%	6.0%	0.04
Acute hypoxemic respiratory failure	10.3%	-2.1%	0.42	-4.3%	-2.1%	0.13
Acute renal failure syndrome	16.8%	13.3%	0.10	12.8%	14.8%	-0.06
Agranulocytosis	-2.7%	-2.1%	0.04	-4.3%	-2.1%	-0.09
Ascites due to alcoholic cirrhosis	-2.7%	-2.1%	-0.04	-4.3%	-2.1%	0.03
Bacterial pneumonia	-2.7%	-2.1%	0.03	-4.3%	-2.1%	0.02
Bronchopneumonia	-2.7%	2.1%	0.01	-4.3%	2.7%	-0.14
Chronic obstructive lung disease	3.8%	2.5%	0.08	-4.3%	3.8%	-0.13
Congestive heart failure	3.3%	2.5%	0.05	-4.3%	-2.1%	0.11
Diastolic heart failure	-2.7%	-2.1%	0.12	-4.3%	-2.1%	0.14
End stage renal disease on dialysis due to type 2 diabetes mellitus	-2.7%	-2.1%	-0.05	-4.3%	-2.1%	-0.10
Heart failure	9.2%	5.4%	0.15	8.5%	5.6%	0.12
Hypertensive heart disease with congestive heart failure	-2.7%	-2.1%	0.02	-4.3%	-2.1%	0.08
Ketoacidosis due to type 2 diabetes mellitus	-2.7%	-2.1%	0.02	-4.3%	-2.1%	0.08
Left heart failure	-2.7%	-2.1%	-0.04	-4.3%	-2.1%	0.03
Localized edema	-2.7%	-2.1%	0.04	-4.3%	2.1%	0.03
Pneumonia	56.0%	25.0%	0.67	38.5%	39.3%	-0.02
Pulmonary edema	-2.7%	-2.1%	0.07	-4.3%	-2.1%	0.11
Pulmonary embolism	2.7%	-2.1%	0.07	-4.3%	-2.1%	0.03
Septic shock	6.5%	5.0%	0.07	-4.3%	6.3%	-0.09
Thrombophlebitis of deep veins of lower extremity	-2.7%	-2.1%	0.08	-4.3%	-2.1%	0.05
Viral pneumonia	17.4%	8.3%	0.27	11.1%	11.5%	-0.01
**Medications**						
Dexamethasone	12.0%	5.0%	0.25	6.0%	7.7%	-0.07
Deflazacort	-2.7%	-2.1%	-0.04	-4.3%	-2.1%	-0.04
Methylprednisolone	2.7%	-2.1%	0.11	-4.3%	-2.1%	0.09
Prednisolone	3.3%	3.3%	0.00	-4.3%	2.1%	0.08

WKUH, Wonkwang University Hospital; PS, Propensity Score; Std. Diff, Standardized Difference.

Supplementary Table S22B. Baseline characteristics before and after propensity-score adjustment with expanded severity covariates: aminopenicillin/β-lactamase inhibitor combinations vs third-generation cephalosporins (WKUH)

Characteristic	Before PS adjustment			After PS adjustment		
	Target (%)	Comparator (%)	Std. Diff	Target (%)	Comparator (%)	Std. Diff
**Age group**						
15-19	-29.4%	-2.1%	0.25	-29.4%	-2.2%	0.24
20-24	-29.4%	-2.1%	0.22	-29.4%	-2.2%	0.21
25-29	-29.4%	2.1%	0.20	-29.4%	2.3%	0.18
45-49	-29.4%	3.8%	0.10	-29.4%	4.0%	0.09
50-54	-29.4%	2.5%	0.37	-29.4%	2.7%	0.36
55-59	-29.4%	5.8%	0.37	-29.4%	6.3%	0.36
60-64	-29.4%	8.3%	0.28	-29.4%	9.0%	0.26
65-69	-29.4%	8.3%	-0.10	-29.4%	8.6%	-0.11
70-74	-29.4%	10.0%	0.06	-29.4%	10.3%	0.05
80-84	-29.4%	17.1%	-0.15	-29.4%	15.6%	-0.11
**Index Year**						
2022	47.1%	49.2%	-0.04	47.1%	44.6%	0.05
2023	47.1%	45.0%	0.04	47.1%	49.5%	-0.05
2024	-29.4%	3.8%	0.10	-29.4%	3.4%	0.12
**Vital signs**						
Body temperature	70.6%	92.1%	-0.57	70.6%	91.6%	-0.56
Diastolic blood pressure	70.6%	92.5%	-0.59	70.6%	92.0%	-0.57
Heart rate	70.6%	91.7%	-0.56	70.6%	91.1%	-0.54
Systolic blood pressure	70.6%	92.5%	-0.59	70.6%	92.0%	-0.57
Respiratory rate	70.6%	91.7%	-0.56	70.6%	91.1%	-0.54
**Laboratory**						
C-reactive protein	88.2%	92.1%	-0.13	88.2%	92.3%	-0.14
**Oxygenation**						
Oxygen saturation in Arterial blood by Pulse oximetry	64.7%	91.7%	-0.69	64.7%	91.1%	-0.67
**Comorbidities**						
Acidosis	-29.4%	4.6%	0.06	-29.4%	3.9%	0.09
Acute renal failure syndrome	-29.4%	13.3%	-0.26	-29.4%	12.6%	-0.23
Bacterial pneumonia	-29.4%	-2.1%	0.25	-29.4%	-2.4%	0.24
Diabetic ketoacidosis	-29.4%	-2.1%	0.28	-29.4%	-2.4%	0.27
Ketoacidosis due to type 2 diabetes mellitus	-29.4%	-2.1%	0.32	-29.4%	-2.4%	0.31
Pneumonia	-29.4%	25.0%	-0.55	-29.4%	20.2%	-0.43
**Medications**						
Dexamethasone	-29.4%	5.0%	0.04	-29.4%	4.4%	0.07
Hydrocortisone	-29.4%	-2.1%	0.32	-29.4%	-2.4%	0.31

WKUH, Wonkwang University Hospital; PS, Propensity Score; Std. Diff, Standardized Difference.

Supplementary Table S23. List of third-generation cephalosporin drug concepts included in the analysis

Concept Id	Name
36230001	Omnicef Pill
36230000	Omnicef Oral Product
36234790	Claforan Injectable Product
21602898	Ceftriaxone; parenteral
21602912	Ceftriaxone, combinations; systemic
42941916	Ceftriaxone Injection [TRIAXONE]
42941910	Ceftriaxone Injection [BORYUNG CEFTRIAXONE]
46287316	Ceftriaxone Injection
36217281	Ceftriaxone Injectable Product
1501779	Ceftriaxone and beta-lactamase inhibitor; systemic
42941849	Ceftriaxone 500 MG Injection [TRIAXONE]
46287321	Ceftriaxone 500 MG Injection
42941863	Ceftriaxone 500 MG [TRIAXONE]
46287320	Ceftriaxone 500 MG
42941696	Ceftriaxone 2000 MG Injection [TRIAXONE]
42941694	Ceftriaxone 2000 MG Injection [BORYUNG CEFTRIAXONE]
46287335	Ceftriaxone 2000 MG Injection
42941719	Ceftriaxone 2000 MG [TRIAXONE]
42941717	Ceftriaxone 2000 MG [BORYUNG CEFTRIAXONE]
46287334	Ceftriaxone 2000 MG
2048064	Ceftriaxone 20 MG/ML / Sodium chloride 9 MG/ML Injectable Solution [TRISONKIT]
2048063	Ceftriaxone 20 MG/ML / Sodium chloride 9 MG/ML Injectable Solution
2048065	Ceftriaxone 20 MG/ML / Sodium chloride 9 MG/ML [TRISONKIT]
21061798	Ceftriaxone 20 MG/ML
42941778	Ceftriaxone 1000 MG Injection [TRIAXONE]
42941772	Ceftriaxone 1000 MG Injection [BORYUNG CEFTRIAXONE]
46287328	Ceftriaxone 1000 MG Injection
42941830	Ceftriaxone 1000 MG [TRIAXONE]
42941825	Ceftriaxone 1000 MG [BORYUNG CEFTRIAXONE]
46287327	Ceftriaxone 1000 MG
2048073	Ceftriaxone / Sodium chloride Injectable Solution [TRISONKIT]
2048071	Ceftriaxone / Sodium chloride Injectable Solution
1777806	Ceftriaxone
21602901	Ceftizoxime; parenteral
42944738	Ceftizoxime Injection [EPOCELIN]
42944737	Ceftizoxime Injection
42944729	Ceftizoxime 1000 MG Injection [EPOCELIN]
42944728	Ceftizoxime 1000 MG Injection
42944733	Ceftizoxime 1000 MG [EPOCELIN]
44027343	Ceftizoxime 1000 MG
1777254	Ceftizoxime
21602907	Cefpodoxime; oral
36226275	Cefpodoxime Pill
42972424	Cefpodoxime Oral Tablet [BANAN]
40092561	Cefpodoxime Oral Tablet
42972436	Cefpodoxime Oral Suspension [BANAN]
40092559	Cefpodoxime Oral Suspension
36226274	Cefpodoxime Oral Product
36223942	Cefpodoxime Oral Liquid Product
715907	Cefpodoxime and beta-lactamase inhibitor; systemic
42972363	Cefpodoxime 100 MG Oral Tablet [BANAN]
19074932	Cefpodoxime 100 MG Oral Tablet
42972375	Cefpodoxime 100 MG [BANAN]
1749056	Cefpodoxime 100 MG
42972386	Cefpodoxime 10 MG/ML Oral Suspension [BANAN]
19074935	Cefpodoxime 10 MG/ML Oral Suspension
42972405	Cefpodoxime 10 MG/ML [BANAN]
19085867	Cefpodoxime 10 MG/ML
1749008	Cefpodoxime
21602905	Cefpiramide; parenteral
42959786	Cefpiramide sodium Injection [YUHAN CEFPIRAN]

42959784	Cefpiramide sodium Injection
42959776	Cefpiramide sodium 1000 MG Injection [YUHAN CEFPIRAN]
42959774	Cefpiramide sodium 1000 MG Injection
42959779	Cefpiramide sodium 1000 MG [YUHAN CEFPIRAN]
42959777	Cefpiramide sodium 1000 MG
43009045	Cefpiramide sodium
21602895	Cefotaxime; parenteral
42942227	Cefotaxime Injection [HANMI CEFOTAXIME SODIUM]
46275708	Cefotaxime Injection [Claforan]
46275705	Cefotaxime Injection
36217274	Cefotaxime Injectable Product
45893191	Cefotaxime and Beta-lactamase inhibitor; systemic
42942168	Cefotaxime 1000 MG Injection [HANMI CEFOTAXIME SODIUM]
46275709	Cefotaxime 1000 MG Injection [Claforan]
46275706	Cefotaxime 1000 MG Injection
42942187	Cefotaxime 1000 MG [HANMI CEFOTAXIME SODIUM]
46275707	Cefotaxime 1000 MG [Claforan]
46275704	Cefotaxime 1000 MG
1774470	Cefotaxime
21602906	Cefoperazone; parenteral
21602913	Cefoperazone and Beta-lactamase inhibitor; parenteral
42944070	Cefoperazone 500 MG / Sulbactam 500 MG Injection [SULPERAZON]
42944071	Cefoperazone 500 MG / Sulbactam 500 MG Injection [CEFOLATAM]
42944066	Cefoperazone 500 MG / Sulbactam 500 MG Injection [CEFOBACTAM]
42944062	Cefoperazone 500 MG / Sulbactam 500 MG Injection
42944079	Cefoperazone 500 MG / Sulbactam 500 MG [SULPERAZON]
42944080	Cefoperazone 500 MG / Sulbactam 500 MG [CEFOLATAM]
42944075	Cefoperazone 500 MG / Sulbactam 500 MG [CEFOBACTAM]
41170890	Cefoperazone 500 MG
42944089	Cefoperazone / Sulbactam Injection [SULPERAZON]
42944090	Cefoperazone / Sulbactam Injection [CEFOLATAM]
42944085	Cefoperazone / Sulbactam Injection [CEFOBACTAM]
42944083	Cefoperazone / Sulbactam Injection
1773402	Cefoperazone
21602903	Cefodizime; parenteral
42936683	Cefodizime Injection [NEWDIZIME]
42936682	Cefodizime Injection
2045164	Cefodizime 1000 MG Injection [NEWDIZIME] by Daewoong
42936680	Cefodizime 1000 MG Injection [NEWDIZIME]
42936679	Cefodizime 1000 MG Injection
42936681	Cefodizime 1000 MG [NEWDIZIME]
41076949	Cefodizime 1000 MG
19028286	Cefodizime
21602902	Cefixime; oral
36217270	Cefixime Pill
36217134	Cefixime Oral Product
42943374	Cefixime Oral Capsule [DONG-A SUPRAX]
40024406	Cefixime Oral Capsule
954914	Cefixime and Beta-lactamase inhibitor; oral
42943323	Cefixime 100 MG Oral Capsule [DONG-A SUPRAX]
42943315	Cefixime 100 MG Oral Capsule
42943343	Cefixime 100 MG [DONG-A SUPRAX]
40229691	Cefixime 100 MG
1796435	Cefixime
21602910	Cefditoren; oral
36223936	Cefditoren Pill
42972211	Cefditoren Oral Tablet [BORYUNG MEIACT]
40092488	Cefditoren Oral Tablet
36223935	Cefditoren Oral Product
42972210	Cefditoren Oral Granules [BORYUNG MEIACT]
42972209	Cefditoren Oral Granules
42972202	Cefditoren 10000 MG Oral Granules [BORYUNG MEIACT]
42972201	Cefditoren 10000 MG Oral Granules
42972204	Cefditoren 10000 MG [BORYUNG MEIACT]

42972203	Cefditoren 10000 MG
42972206	Cefditoren 100 MG Oral Tablet [BORYUNG MEIACT]
42972205	Cefditoren 100 MG Oral Tablet
42972208	Cefditoren 100 MG [BORYUNG MEIACT]
42972207	Cefditoren 100 MG
1747005	Cefditoren
21602909	Cefdinir; oral
36223934	Cefdinir Pill
36223933	Cefdinir Oral Product
42924605	Cefdinir Oral Granules [OMNICEF FOR CHILDREN]
42924604	Cefdinir Oral Granules
40092164	Cefdinir Oral Capsule [Omnicef]
40092162	Cefdinir Oral Capsule
42924579	Cefdinir 10000 MG Oral Granules [OMNICEF FOR CHILDREN]
42924578	Cefdinir 10000 MG Oral Granules
42924584	Cefdinir 10000 MG [OMNICEF FOR CHILDREN]
42924583	Cefdinir 10000 MG
42924589	Cefdinir 100 MG Oral Capsule [Omnicef]
42924590	Cefdinir 100 MG Oral Capsule
42924597	Cefdinir 100 MG [Omnicef]
42924540	Cefdinir 100 MG
1796458	Cefdinir
21602911	Cefcapene; oral
42961738	Cefcapene pivoxil hydrochloride hydrate Oral Tablet [FLOMOX]
42961736	Cefcapene pivoxil hydrochloride hydrate Oral Tablet
42961727	Cefcapene pivoxil hydrochloride hydrate 100 MG Oral Tablet [FLOMOX]
42961725	Cefcapene pivoxil hydrochloride hydrate 100 MG Oral Tablet
42961731	Cefcapene pivoxil hydrochloride hydrate 100 MG [FLOMOX]
42961729	Cefcapene pivoxil hydrochloride hydrate 100 MG
43009044	Cefcapene pivoxil hydrochloride hydrate
42918770	500 ML cefpodoxime 10 MG/ML Oral Suspension [BANAN]
42918769	500 ML cefpodoxime 10 MG/ML Oral Suspension
42918973	200 ML cefpodoxime 10 MG/ML Oral Suspension [BANAN]
41434076	200 ML cefpodoxime 10 MG/ML Oral Suspension
2025800	100 ML ceftriaxone 20 MG/ML / Sodium chloride 9 MG/ML Injectable Solution [TRISONKIT] by UK Chemipharm
2025799	100 ML ceftriaxone 20 MG/ML / Sodium chloride 9 MG/ML Injectable Solution [TRISONKIT]
2025798	100 ML ceftriaxone 20 MG/ML / Sodium chloride 9 MG/ML Injectable Solution

Supplementary Table S24. List of fluoroquinolones drug concepts included in the analysis

Concept Id	Name
715910	Tosufloxacin; oral
42962235	Tosufloxacin tosylate Oral Tablet [OZEX]
42962234	Tosufloxacin tosylate Oral Tablet
42962231	Tosufloxacin tosylate 150 MG Oral Tablet [OZEX]
42962230	Tosufloxacin tosylate 150 MG Oral Tablet
42962233	Tosufloxacin tosylate 150 MG [OZEX]
42962232	Tosufloxacin tosylate 150 MG
43009011	Tosufloxacin tosylate
21603008	Ofloxacin; systemic
42953087	Ofloxacin Oral Tablet [JEIL TARIVID]
40069653	Ofloxacin Oral Tablet
42952962	Ofloxacin 100 MG Oral Tablet [JEIL TARIVID]
19106088	Ofloxacin 100 MG Oral Tablet
42953002	Ofloxacin 100 MG [JEIL TARIVID]
19111091	Ofloxacin 100 MG
923081	Ofloxacin
21603021	Moxifloxacin; systemic
40057470	Moxifloxacin Oral Tablet [Avelox]
40057469	Moxifloxacin Oral Tablet
2051320	Moxifloxacin Injectable Solution [MOROXACIN]
42944574	Moxifloxacin Injectable Solution [Avelox]
36888537	Moxifloxacin Injectable Solution
1716904	Moxifloxacin 400 MG Oral Tablet [Avelox]
1716905	Moxifloxacin 400 MG Oral Tablet
19099248	Moxifloxacin 400 MG [Avelox]
19085094	Moxifloxacin 400 MG
2051264	Moxifloxacin 1.6 MG/ML Injectable Solution [MOROXACIN]
42944536	Moxifloxacin 1.6 MG/ML Injectable Solution [Avelox]
36890868	Moxifloxacin 1.6 MG/ML Injectable Solution
2051265	Moxifloxacin 1.6 MG/ML [MOROXACIN]
40165696	Moxifloxacin 1.6 MG/ML [Avelox]
19098000	Moxifloxacin 1.6 MG/ML
1716903	Moxifloxacin
21603019	levofloxacin; systemic
42965605	Levofloxacin Oral Tablet [LEVOBACTER]
2050211	Levofloxacin Oral Tablet [LEFLOXIN]
42965560	Levofloxacin Oral Tablet [JEIL CRAVIT]
42965579	Levofloxacin Oral Tablet [CRAVIT]
40001160	Levofloxacin Oral Tablet
42965641	Levofloxacin Injectable Solution [LEVOFLOXACIN CJ]
42965648	Levofloxacin Injectable Solution [LEVOFEXIN]
42965639	Levofloxacin Injectable Solution [CRAVIT]
42479758	Levofloxacin Injectable Solution
2050057	Levofloxacin 500 MG Oral Tablet [LEVOBACTER] by Seoul
2050056	Levofloxacin 500 MG Oral Tablet [LEVOBACTER]
42965215	Levofloxacin 500 MG Oral Tablet [CRAVIT]
1742255	Levofloxacin 500 MG Oral Tablet
2050082	Levofloxacin 500 MG [LEVOBACTER]
42965228	Levofloxacin 500 MG [CRAVIT]
19082484	Levofloxacin 500 MG
42965322	Levofloxacin 5 MG/ML Injectable Solution [LEVOFLOXACIN CJ]
42965330	Levofloxacin 5 MG/ML Injectable Solution [LEVOFEXIN]
42965320	Levofloxacin 5 MG/ML Injectable Solution [CRAVIT]
42479165	Levofloxacin 5 MG/ML Injectable Solution
42965359	Levofloxacin 5 MG/ML [LEVOKACIN]
42965358	Levofloxacin 5 MG/ML [LEVOFLOXACIN CJ]
42965376	Levofloxacin 5 MG/ML [LEVOFEXIN]
42965349	Levofloxacin 5 MG/ML [CRAVIT]
1742288	Levofloxacin 5 MG/ML
2050134	Levofloxacin 100 MG Oral Tablet [LEFLOXIN] by Handok
2050133	Levofloxacin 100 MG Oral Tablet [LEFLOXIN] by Aestura

2050132	Levofloxacin 100 MG Oral Tablet [LEFLOXIN]
42965387	Levofloxacin 100 MG Oral Tablet [JEIL CRAVIT]
19107185	Levofloxacin 100 MG Oral Tablet
2050160	Levofloxacin 100 MG [LEFLOXIN]
42965450	Levofloxacin 100 MG [JEIL CRAVIT]
19110433	Levofloxacin 100 MG
1742253	Levofloxacin
21603022	Gemifloxacin; oral
40059633	Gemifloxacin Oral Tablet [Factive]
40059632	Gemifloxacin Oral Tablet
42932492	Gemifloxacin Injection [Factive]
42932493	Gemifloxacin Injection
1716744	Gemifloxacin 320 MG Oral Tablet [Factive]
19124327	Gemifloxacin 320 MG Oral Tablet
19045211	Gemifloxacin 320 MG [Factive]
1716742	Gemifloxacin 320 MG
42932488	Gemifloxacin 200 MG Injection [Factive]
42932489	Gemifloxacin 200 MG Injection
42932490	Gemifloxacin 200 MG [Factive]
42932491	Gemifloxacin 200 MG
1716721	Gemifloxacin
21603007	Fluoroquinolones
21603015	Fleroxacin; systemic
19050750	Fleroxacin
21603009	Ciprofloxacin; systemic
42948348	Ciprofloxacin Oral Tablet [CYCIN]
40028726	Ciprofloxacin Oral Tablet
46287381	Ciprofloxacin Injection
42948390	Ciprofloxacin Injectable Solution [CITOPCIN]
42948395	Ciprofloxacin Injectable Solution [CHOONGWAE CIPROFLOXACIN]
40028715	Ciprofloxacin Injectable Solution
42948399	Ciprofloxacin Extended Release Oral Tablet [CIPROURO]
40127787	Ciprofloxacin Extended Release Oral Tablet
42948272	Ciprofloxacin 500 MG Extended Release Oral Tablet [CIPROURO]
1797580	Ciprofloxacin 500 MG Extended Release Oral Tablet
42948276	Ciprofloxacin 500 MG [CIPROURO]
1797557	Ciprofloxacin 500 MG
42948181	Ciprofloxacin 250 MG Oral Tablet [CYCIN]
1797515	Ciprofloxacin 250 MG Oral Tablet
42948238	Ciprofloxacin 250 MG [CYCIN]
1797556	Ciprofloxacin 250 MG
46287383	Ciprofloxacin 2 MG/ML Injection
42948291	Ciprofloxacin 2 MG/ML Injectable Solution [CITOPCIN]
42948296	Ciprofloxacin 2 MG/ML Injectable Solution [CHOONGWAE CIPROFLOXACIN]
36892783	Ciprofloxacin 2 MG/ML Injectable Solution
42948310	Ciprofloxacin 2 MG/ML [CITOPCIN]
42948315	Ciprofloxacin 2 MG/ML [CHOONGWAE CIPROFLOXACIN]
1797555	Ciprofloxacin 2 MG/ML
46287391	Ciprofloxacin 10 MG/ML Injection
40223510	Ciprofloxacin 10 MG/ML
1797513	Ciprofloxacin
2026276	50 ML levofloxacin 5 MG/ML Injectable Solution by CJ
2026271	50 ML levofloxacin 5 MG/ML Injectable Solution [CRAVIT] by Jeil
42919971	50 ML Levofloxacin 5 MG/ML Injectable Solution [CRAVIT]
36896008	50 ML Levofloxacin 5 MG/ML Injectable Solution
2026238	50 ML ciprofloxacin 2 MG/ML Injectable Solution [CITOPCIN] by CJ
42919791	50 ML Ciprofloxacin 2 MG/ML Injectable Solution [CITOPCIN]
36896011	50 ML Ciprofloxacin 2 MG/ML Injectable Solution
2025250	250 ML moxifloxacin 1.6 MG/ML Injectable Solution [MOROXACIN] by Hanmi
2025249	250 ML moxifloxacin 1.6 MG/ML Injectable Solution [MOROXACIN]
42918875	250 ML moxifloxacin 1.6 MG/ML Injectable Solution [Avelox]
41437171	250 ML moxifloxacin 1.6 MG/ML Injectable Solution
42918949	200 ML Ciprofloxacin 2 MG/ML Injectable Solution [CITOPCIN]
42918954	200 ML Ciprofloxacin 2 MG/ML Injectable Solution [CHOONGWAE CIPROFLOXACIN]

36896594	200 ML Ciprofloxacin 2 MG/ML Injectable Solution
42919103	150 ML Levofloxacin 5 MG/ML Injectable Solution [LEVOFLOXACIN CJ]
42919106	150 ML Levofloxacin 5 MG/ML Injectable Solution [LEVOFEXIN]
42919102	150 ML Levofloxacin 5 MG/ML Injectable Solution [CRAVIT]
42919096	150 ML Levofloxacin 5 MG/ML Injectable Solution
42919480	100 ML Levofloxacin 5 MG/ML Injectable Solution [LEVOFLOXACIN CJ]
2025823	100 ML levofloxacin 5 MG/ML Injectable Solution [LEVOFEXIN] by Ildong
42919487	100 ML Levofloxacin 5 MG/ML Injectable Solution [LEVOFEXIN]
2025834	100 ML levofloxacin 5 MG/ML Injectable Solution [CRAVIT] by Jeil
42919478	100 ML Levofloxacin 5 MG/ML Injectable Solution [CRAVIT]
42482686	100 ML Levofloxacin 5 MG/ML Injectable Solution
42919374	100 ML Ciprofloxacin 2 MG/ML Injectable Solution [CITOPCIN]
36896428	100 ML Ciprofloxacin 2 MG/ML Injectable Solution

Supplementary Table S25. List of aminopenicillin/β-lactamase inhibitor combinations drug concepts included in the analysis

Concept Id	Name
21602862	Ampicillin and Beta-lactamase inhibitor; parenteral
42966044	Ampicillin 500 MG / Sulbactam 250 MG Injection [Unasyn]
42966046	Ampicillin 500 MG / Sulbactam 250 MG Injection [RUKASYN]
42966045	Ampicillin 500 MG / Sulbactam 250 MG Injection
42966058	Ampicillin 500 MG / Sulbactam 250 MG [Unasyn]
42966059	Ampicillin 500 MG / Sulbactam 250 MG [RUKASYN]
42966036	Ampicillin 1000 MG / Sulbactam 500 MG Injection [BACTACIN]
46275616	Ampicillin 1000 MG / Sulbactam 500 MG Injection
42966042	Ampicillin 1000 MG / Sulbactam 500 MG [BACTACIN]
46275618	Ampicillin / Sulbactam Injection [Unasyn]
42966071	Ampicillin / Sulbactam Injection [RUKASYN]
42966076	Ampicillin / Sulbactam Injection [BACTACIN]
46275615	Ampicillin / Sulbactam Injection
21602863	Amoxicillin and Beta-lactamase inhibitor; systemic
2051755	Amoxicillin 6000 MG / Clavulanate 429 MG Oral Suspension [AMOCRANEO] by Kuhnil
2051754	Amoxicillin 6000 MG / Clavulanate 429 MG Oral Suspension [AMOCRANEO]
2051753	Amoxicillin 6000 MG / Clavulanate 429 MG Oral Suspension
2051788	Amoxicillin 6000 MG / Clavulanate 429 MG [AMOCRANEO]
42951267	Amoxicillin 500 MG / Sulbactam 250 MG Injection [SULTAMOX]
42951266	Amoxicillin 500 MG / Sulbactam 250 MG Injection
42951268	Amoxicillin 500 MG / Sulbactam 250 MG [SULTAMOX]
19133021	Amoxicillin 500 MG / Clavulanate 125 MG Oral Tablet [Augmentin]
1713694	Amoxicillin 500 MG / Clavulanate 125 MG Oral Tablet
19133020	Amoxicillin 500 MG / Clavulanate 125 MG [Augmentin]
42951308	Amoxicillin 500 MG / Clavulanate 100 MG Injection [AMOCLA]
21042264	Amoxicillin 500 MG / Clavulanate 100 MG Injection
42951314	Amoxicillin 500 MG / Clavulanate 100 MG [AMOCLA]
2051499	Amoxicillin 50 MG / Sulbactam 50 MG Oral Suspension [SULTAMOX] by Alvogen
2051498	Amoxicillin 50 MG / Sulbactam 50 MG Oral Suspension [SULTAMOX]
2051526	Amoxicillin 50 MG / Sulbactam 50 MG Oral Suspension
2051565	Amoxicillin 50 MG / Sulbactam 50 MG [SULTAMOX]
42951834	Amoxicillin 400 MG / Clavulanate 57 MG Oral Tablet [AMOXCLAN DUO]
42951831	Amoxicillin 400 MG / Clavulanate 57 MG Oral Tablet
42951857	Amoxicillin 400 MG / Clavulanate 57 MG [AMOXCLAN DUO]
42951526	Amoxicillin 40 MG/ML / Clavulanate 5.7 MG/ML Oral Suspension [Augmentin Duo]
1713697	Amoxicillin 40 MG/ML / Clavulanate 5.7 MG/ML Oral Suspension
42951608	Amoxicillin 40 MG/ML / Clavulanate 5.7 MG/ML [Augmentin Duo]
2052418	Amoxicillin 40 MG / Clavulanate 5.7 MG Oral Suspension [AMOCLA DUO] by Kuhnil
2052417	Amoxicillin 40 MG / Clavulanate 5.7 MG Oral Suspension [AMOCLA DUO]
2052421	Amoxicillin 40 MG / Clavulanate 5.7 MG Oral Suspension
2052510	Amoxicillin 40 MG / Clavulanate 5.7 MG [AMOCLA DUO]
42951225	Amoxicillin 250 MG / Sulbactam 250 MG Oral Tablet [SULTAMOX]
1836242	Amoxicillin 250 MG / Sulbactam 250 MG Oral Tablet
42951254	Amoxicillin 250 MG / Sulbactam 250 MG [SULTAMOX]
42951403	Amoxicillin 250 MG / Clavulanate 125 MG Oral Tablet [TIRAMOX]
42951394	Amoxicillin 250 MG / Clavulanate 125 MG Oral Tablet [AMOCLA]
1713671	Amoxicillin 250 MG / Clavulanate 125 MG Oral Tablet
42951480	Amoxicillin 250 MG / Clavulanate 125 MG [TIRAMOX]
42951471	Amoxicillin 250 MG / Clavulanate 125 MG [AMOCLA]
2052274	Amoxicillin 25 MG / Clavulanate 6.25 MG Oral Suspension [Augmentin] by Ilsung
2052273	Amoxicillin 25 MG / Clavulanate 6.25 MG Oral Suspension [Augmentin]
2052275	Amoxicillin 25 MG / Clavulanate 6.25 MG Oral Suspension
2052298	Amoxicillin 25 MG / Clavulanate 6.25 MG [Augmentin]
42951875	Amoxicillin 125 MG / Clavulanate 62.5 MG Oral Tablet [Augmentin]
42951876	Amoxicillin 125 MG / Clavulanate 62.5 MG Oral Tablet
42951879	Amoxicillin 125 MG / Clavulanate 62.5 MG [Augmentin]
42951893	Amoxicillin 120 MG/ML / Clavulanate 8.58 MG/ML Oral Suspension [AMOCLA NEO]
1760056	Amoxicillin 120 MG/ML / Clavulanate 8.58 MG/ML Oral Suspension
42951929	Amoxicillin 120 MG/ML / Clavulanate 8.58 MG/ML [AMOCLA NEO]
42951270	Amoxicillin 1000 MG / Sulbactam 500 MG Injection [SULTAMOX]

42951269	Amoxicillin 1000 MG / Sulbactam 500 MG Injection
42951271	Amoxicillin 1000 MG / Sulbactam 500 MG [SULTAMOX]
42951957	Amoxicillin 1000 MG / Clavulanate 200 MG Injection [TIRAMOX]
42951956	Amoxicillin 1000 MG / Clavulanate 200 MG Injection [AMOCLA]
21160118	Amoxicillin 1000 MG / Clavulanate 200 MG Injection
42951962	Amoxicillin 1000 MG / Clavulanate 200 MG [TIRAMOX]
42951961	Amoxicillin 1000 MG / Clavulanate 200 MG [AMOCLA]
42951295	Amoxicillin / Sulbactam Oral Tablet [SULTAMOX]
40009223	Amoxicillin / Sulbactam Oral Tablet
42951304	Amoxicillin / Sulbactam Oral Suspension [SULTAMOX]
40009222	Amoxicillin / Sulbactam Oral Suspension
42951273	Amoxicillin / Sulbactam Injection [SULTAMOX]
42951272	Amoxicillin / Sulbactam Injection
42952068	Amoxicillin / Clavulanate Oral Tablet [TIRAMOX]
40155192	Amoxicillin / Clavulanate Oral Tablet [Augmentin]
42951977	Amoxicillin / Clavulanate Oral Tablet [AMOXCLAN DUO]
42952056	Amoxicillin / Clavulanate Oral Tablet [AMOCLA]
40105046	Amoxicillin / Clavulanate Oral Tablet
40131078	Amoxicillin / Clavulanate Oral Suspension [Augmentin]
42952078	Amoxicillin / Clavulanate Oral Suspension [Augmentin Duo]
2052566	Amoxicillin / Clavulanate Oral Suspension [AMOCRANEO]
42952113	Amoxicillin / Clavulanate Oral Suspension [AMOCLA NEO]
42952081	Amoxicillin / Clavulanate Oral Suspension [AMOCLA DUO]
40131077	Amoxicillin / Clavulanate Oral Suspension
42951968	Amoxicillin / Clavulanate Injection [TIRAMOX]
42951967	Amoxicillin / Clavulanate Injection [AMOCLA]
42480682	Amoxicillin / Clavulanate Injection
42919823	50 ML Amoxicillin 40 MG/ML / Clavulanate 5.7 MG/ML Oral Suspension [Augmentin Duo]
42919824	50 ML Amoxicillin 40 MG/ML / Clavulanate 5.7 MG/ML Oral Suspension
42919917	50 ML Amoxicillin 120 MG/ML / Clavulanate 8.58 MG/ML Oral Suspension [AMOCLA NEO]
42919905	50 ML Amoxicillin 120 MG/ML / Clavulanate 8.58 MG/ML Oral Suspension

Supplementary Table S26. Negative control outcomes selected for the study

Concept Id	Name
440193	Wristdrop
4115367	Wrist joint pain
4248870	Visceroptosis
380038	Viral conjunctivitis
140641	Verruca vulgaris
194083	Vaginitis and vulvovaginitis
81893	Ulcerative colitis
4030042	Toxic goiter
437264	Tobacco dependence syndrome
134461	Tietze's disease
378427	Tear film insufficiency
72748	Strain of rotator cuff capsule
81151	Sprain of ankle
443172	Splinter of face, without major open wound
36713918	Somatic dysfunction of lumbar region
254443	Sjögren's syndrome
141932	Senile hyperkeratosis
4169984	Scar
438688	Sarcoidosis
380706	Regular astigmatism
4087647	Rectal mass
81634	Ptotic breast
439790	Psychalgia
46286594	Problem related to lifestyle
373478	Presbyopia
4202045	Postviral fatigue syndrome
4112731	Polyp of intestine
4091513	Passing flatus
192606	Paraplegia
75354	Osteochondropathy
438130	Opioid abuse
140648	Onychomycosis due to dermatophyte
4215978	Onychomycosis
40480893	Nonspecific tuberculin test reaction
136368	Non-toxic multinodular goiter
377572	Noise effects on inner ear
4209423	Nicotine dependence
4134455	Mononeuropathy
4103703	Melena
4156115	Malignant neoplasm of endocrine gland
4083487	Macular drusen
440638	Lyme disease
195873	Leukorrhea
443285	Lesion of cervix
438329	Late effect of motor vehicle accident
434203	Late effect of contusion
432593	Kwashiorkor
75576	Irritable bowel syndrome
196168	Irregular periods
434926	Iridocyclitis
444132	Injury of knee
139099	Ingrowing nail
4344500	Impingement syndrome of shoulder region
140480	Impetigo
374375	Impacted cerumen
4201717	Ileostomy present
4254542	Hypopituitarism
380688	Hypoglycemic coma
441788	Human papilloma virus infection
4012934	Homocystinuria
4038835	Hodgkin's disease (clinical)

4012570	High risk sexual behavior
440329	Herpes zoster without complication
4231770	Hereditary thrombophilia
441818	Hemangioma
433577	Hammer toe
74855	Genital herpes simplex
4166231	Genetic predisposition
40481632	Ganglion cyst
196456	Gallstone
4050747	Fracture of upper limb
259995	Foreign body in orifice
4092896	Feces contents abnormal
4345472	Epstein-Barr virus disease
4170770	Epidermoid cyst
4249170	Epicondylitis
73008	Enthesopathy
433527	Endometriosis (clinical)
200775	Endometrial hyperplasia
441589	Endocarditis
433111	Effects of hunger
45757370	Disproportion of reconstructed breast
443767	Disorder of eye due to diabetes mellitus
4115402	Difficulty sleeping
76786	Derangement of knee
260134	Croup
201606	Crohn's disease
78619	Contusion of knee
134438	Contact dermatitis
46269889	Complication due to Crohn's disease
4201390	Colostomy present
432303	Cocaine abuse
81378	Chondromalacia of patella
438531	Chondromalacia
140842	Changes in skin texture
381581	Chalazion
4213540	Cervical somatic dysfunction
434327	Cannabis abuse
73560	Calcaneal spur
133655	Burn of forearm
4110709	Benign epithelial neoplasm of skin
374923	Bell's palsy
378424	Astigmatism
77650	Aseptic necrosis of bone
437082	Ankylosing spondylitis
73241	Anal and rectal polyp
432595	Amyloidosis
4103640	Amputated foot
376707	Acute conjunctivitis
77965	Acquired trigger finger
137951	Acquired keratoderma
75911	Acquired hallux valgus
44783954	Acid reflux
436634	Acariasis
4092879	Absent kidney
4088290	Absence of breast
199192	Abrasion and/or friction burn of trunk without infection
436409	Abnormal pupil
434165	Abnormal cervical smear

Negative control outcomes are concepts known to not be associated with the target or comparator cohorts, such that we can assume the true relative risk between the two cohorts is 1. Once potential negative control candidates were selected, manual clinical review was performed to exclude any pairs that may have a causal relationship or were similar to the study outcome. The final list of 119 negative outcomes is described above.

Supplementary Figure S1A. Love plot of standardized mean differences before and after propensity score adjustment: fluoroquinolones vs third-generation cephalosporins (AUMC)

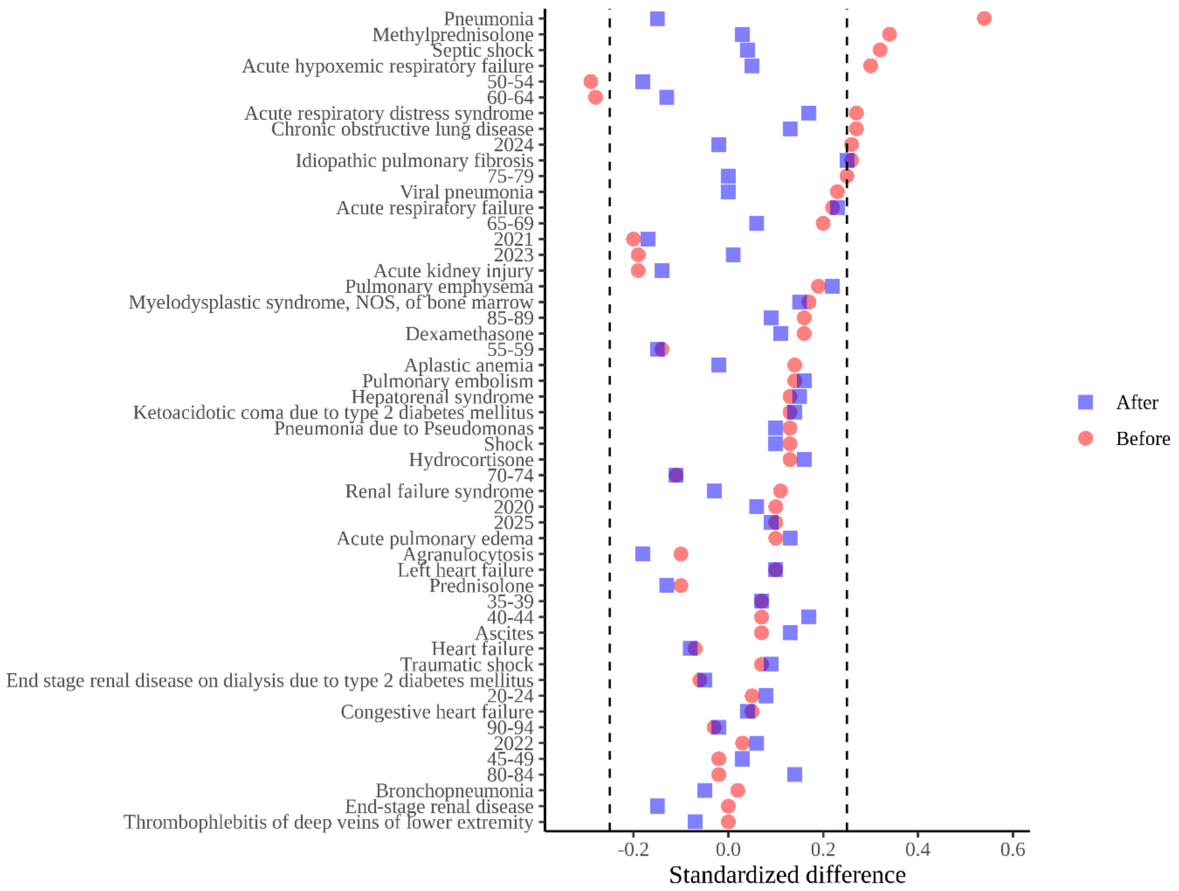

AUMC, Ajou University Medical Center.

Supplementary Figure S1B. Love plot of standardized mean differences before and after propensity score adjustment: aminopenicillin/β-lactamase inhibitor combinations vs third-generation cephalosporins (AUMC)

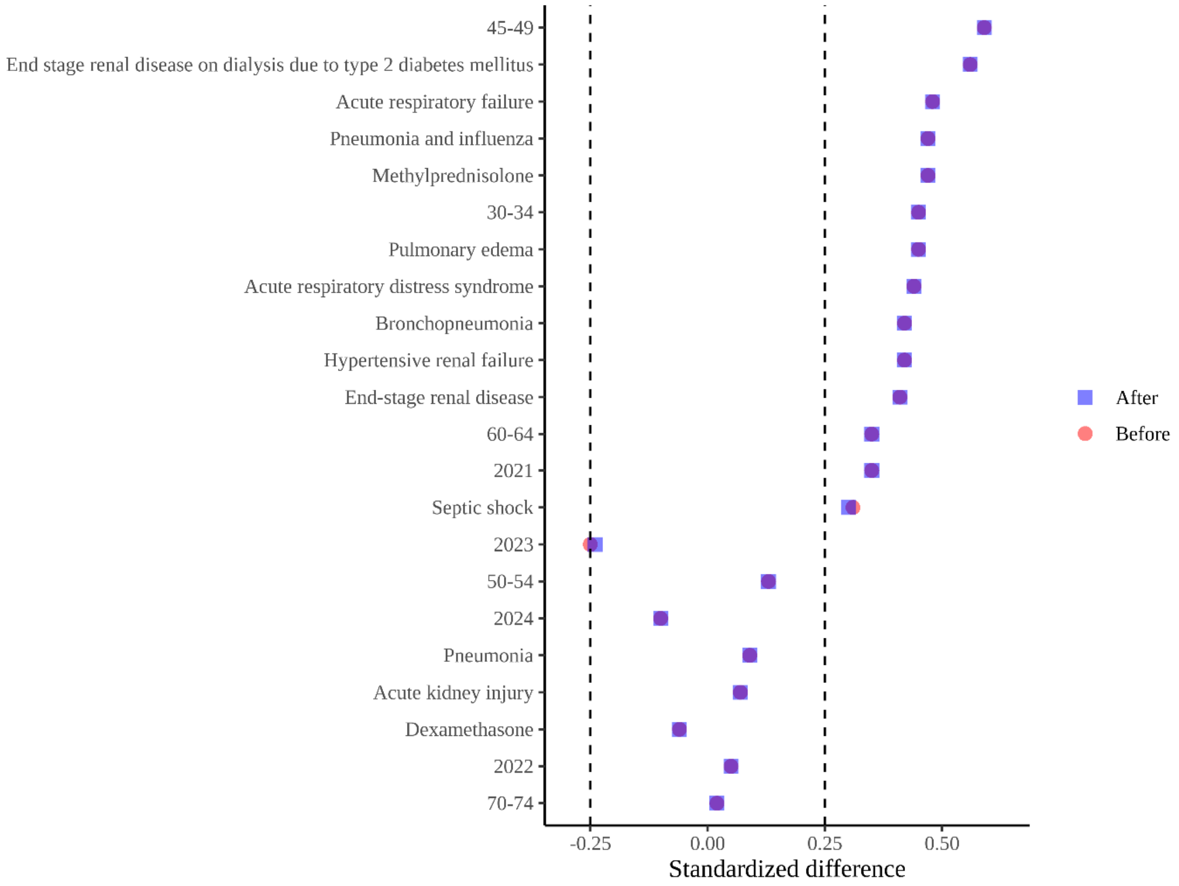

AUMC, Ajou University Medical Center.

Supplementary Figure S2A. Love plot of standardized mean differences before and after propensity score adjustment: fluoroquinolones vs third-generation cephalosporins (EUMC)

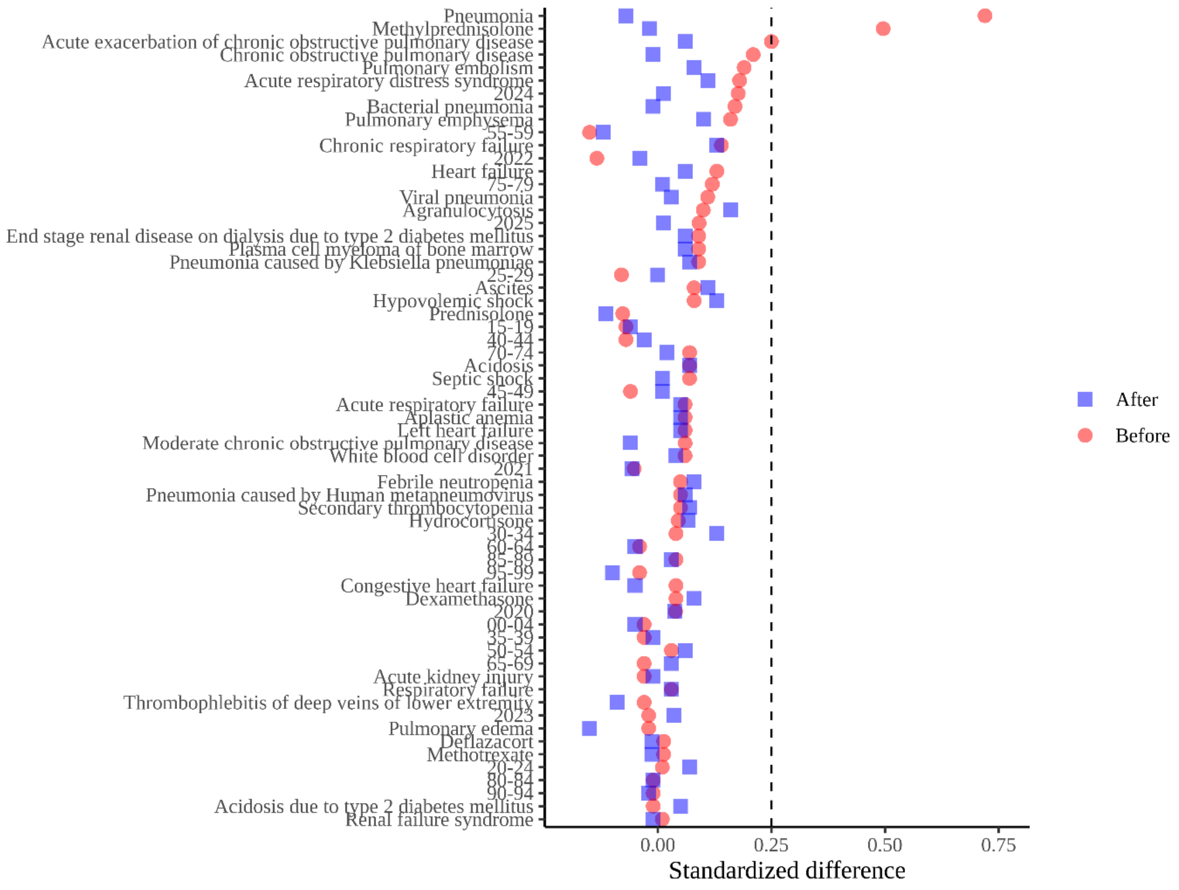

EUMC, Ewha Womans University Medical Center.

Supplementary Figure S2B. Love plot of standardized mean differences before and after propensity score adjustment: aminopenicillin/β-lactamase inhibitor combinations vs third-generation cephalosporins (EUMC)

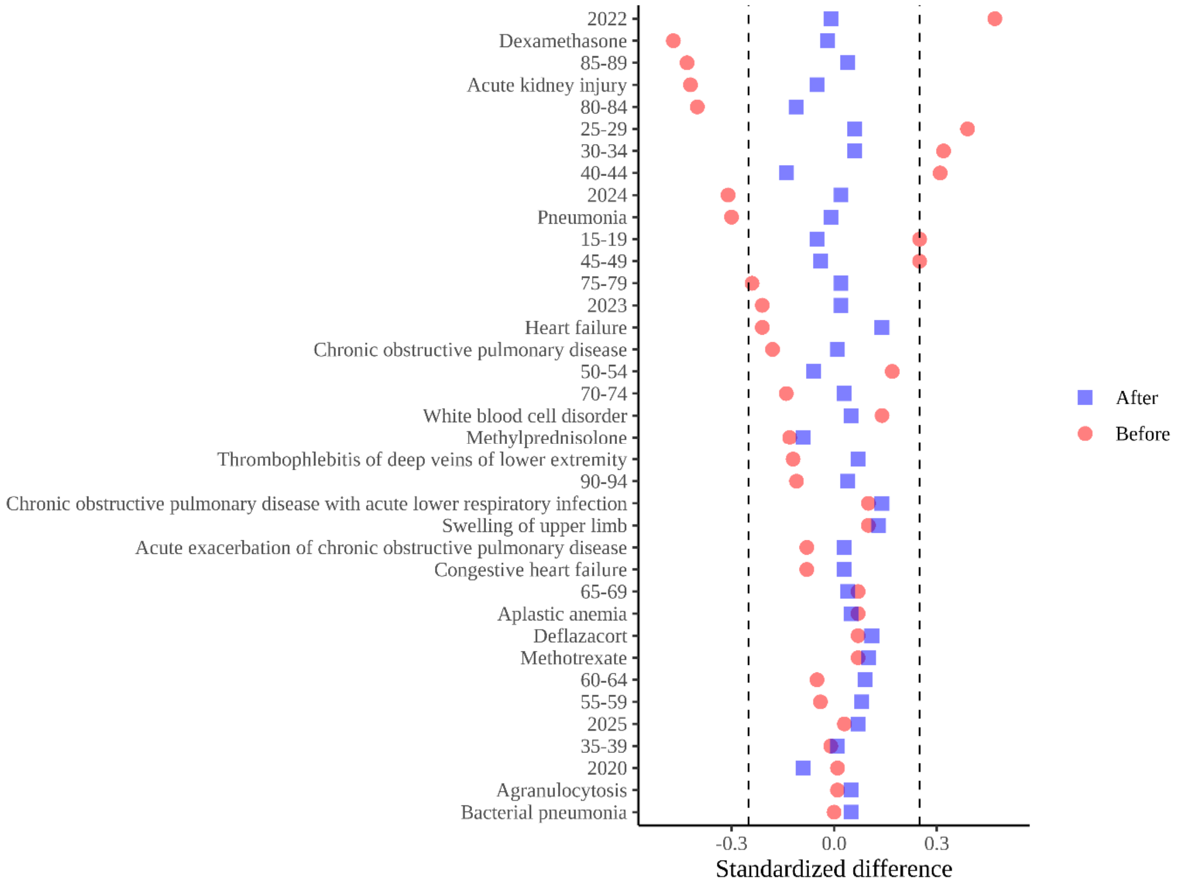

EUMC, Ewha Womans University Medical Center.

Supplementary Figure S3A. Love plot of standardized mean differences before and after propensity score adjustment: fluoroquinolones vs third-generation cephalosporins (GNUH)

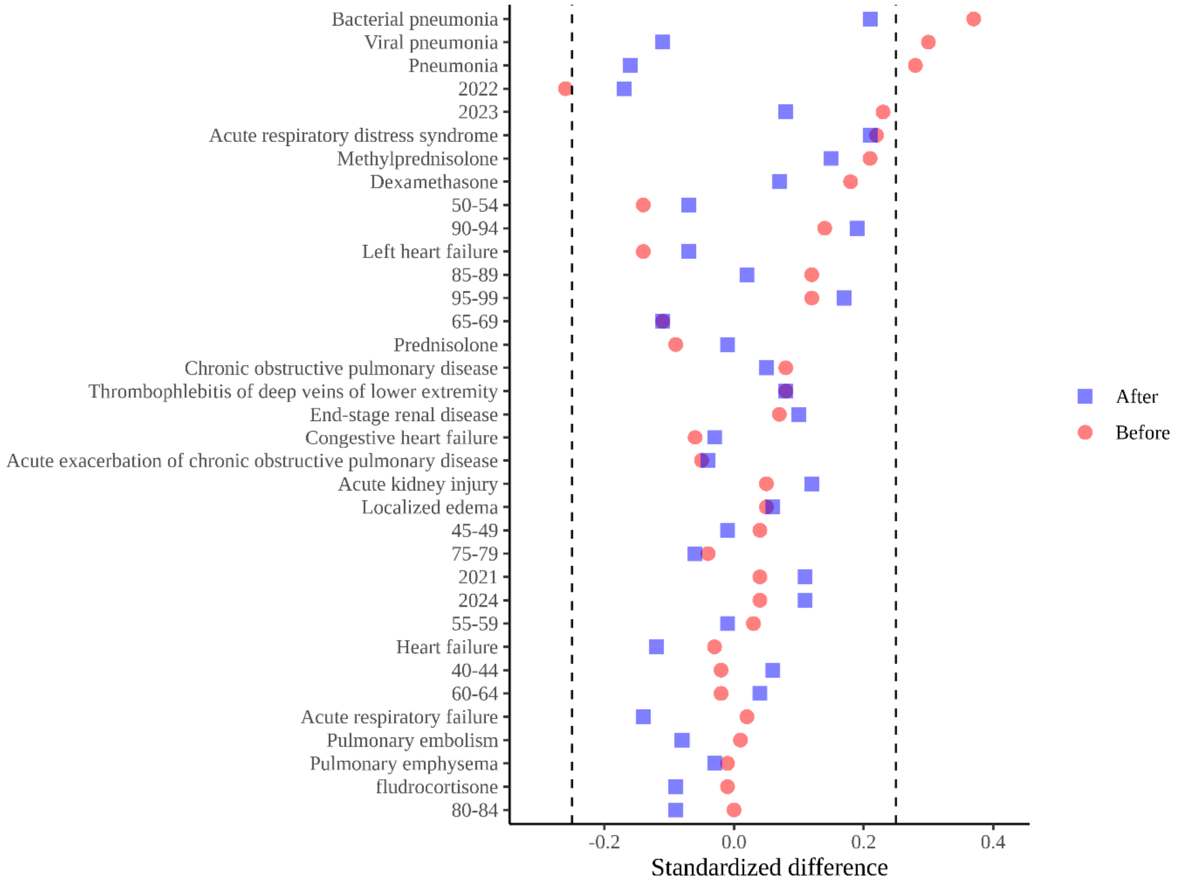

GNUH, Gyeongsang National University Hospital.

Supplementary Figure S3B. Love plot of standardized mean differences before and after propensity score adjustment: aminopenicillin/β-lactamase inhibitor combinations vs third-generation cephalosporins (GNUH)

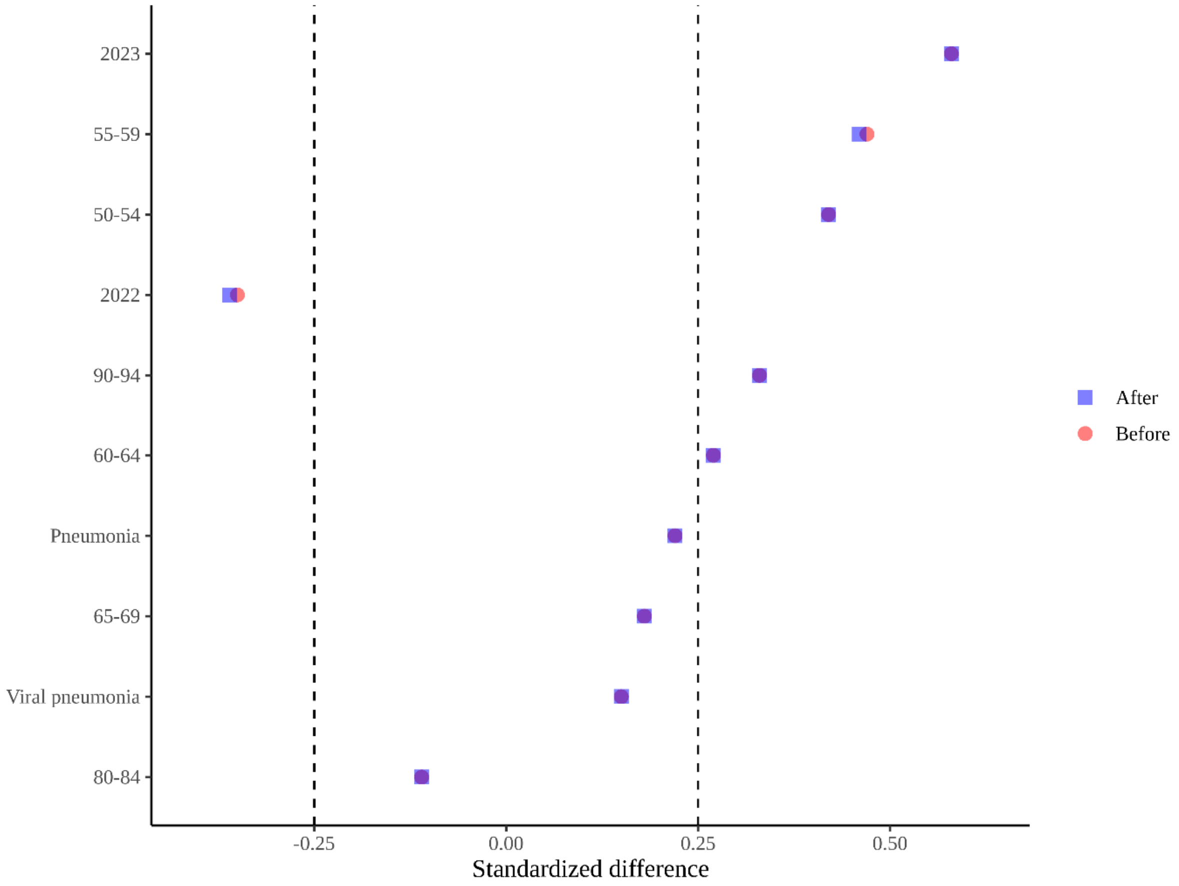

GNUH, Gyeongsang National University Hospital.

Supplementary Figure S4A. Love plot of standardized mean differences before and after propensity score adjustment: fluoroquinolones vs third-generation cephalosporins (ISH)

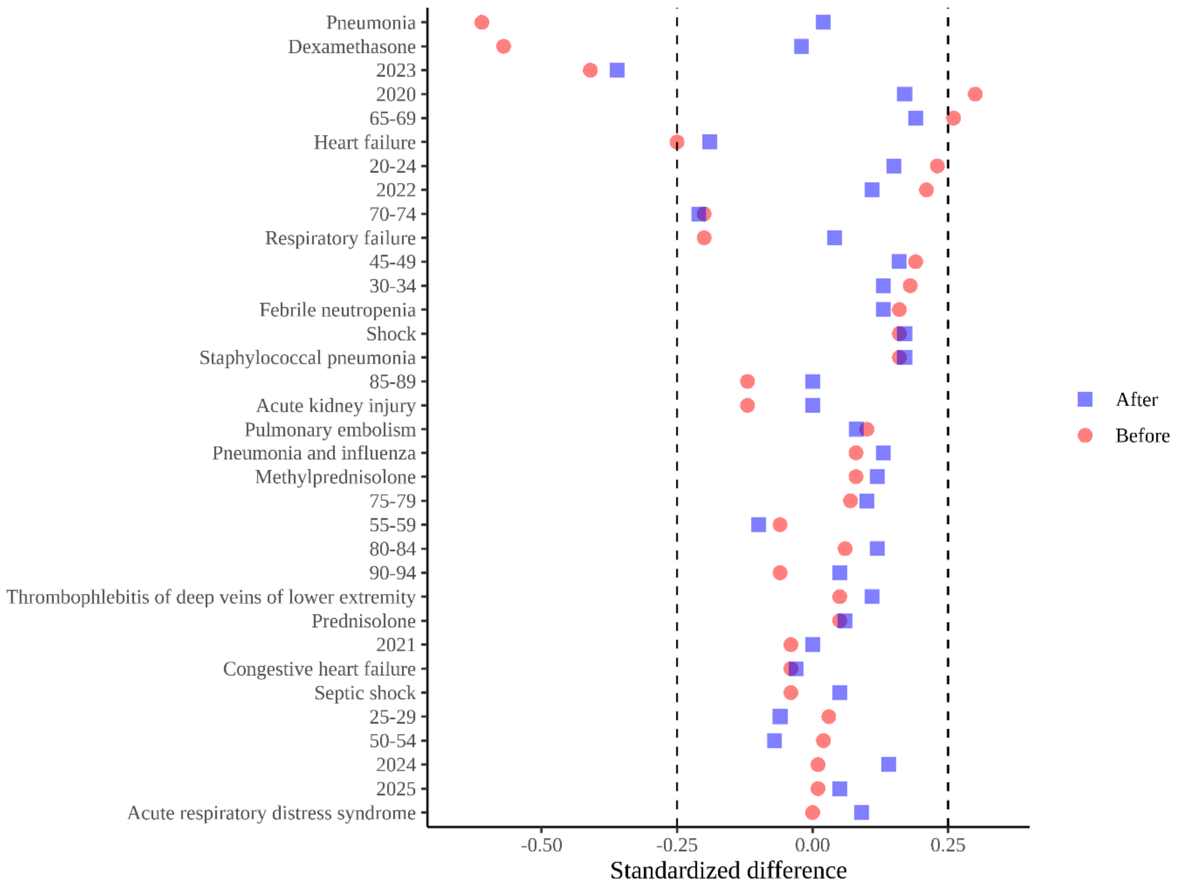

ISH, International St. Mary's Hospital.

Supplementary Figure S4B. Love plot of standardized mean differences before and after propensity score adjustment: aminopenicillin/β-lactamase inhibitor combinations vs third-generation cephalosporins (ISH)

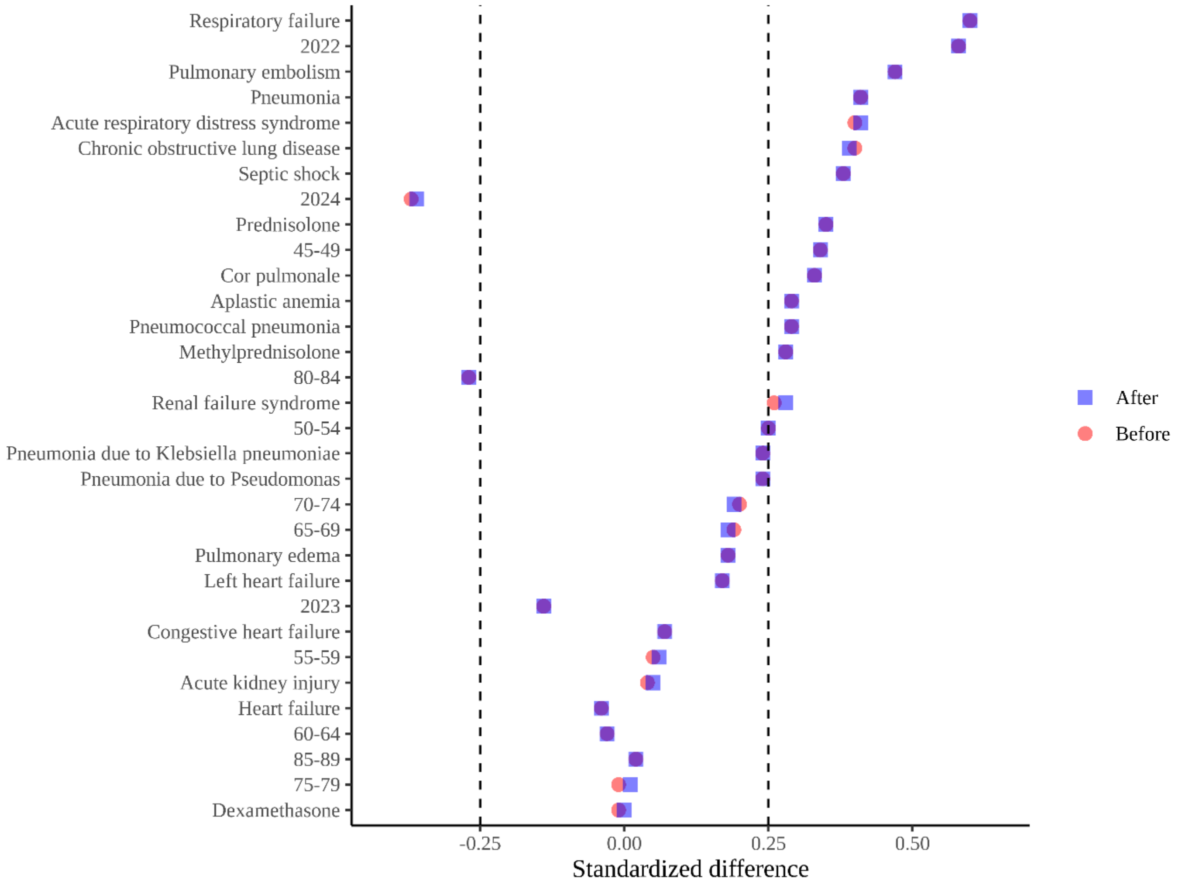

ISH, International St. Mary's Hospital.

Supplementary Figure S5A. Love plot of standardized mean differences before and after propensity score adjustment: fluoroquinolones vs third-generation cephalosporins (JCMJ)

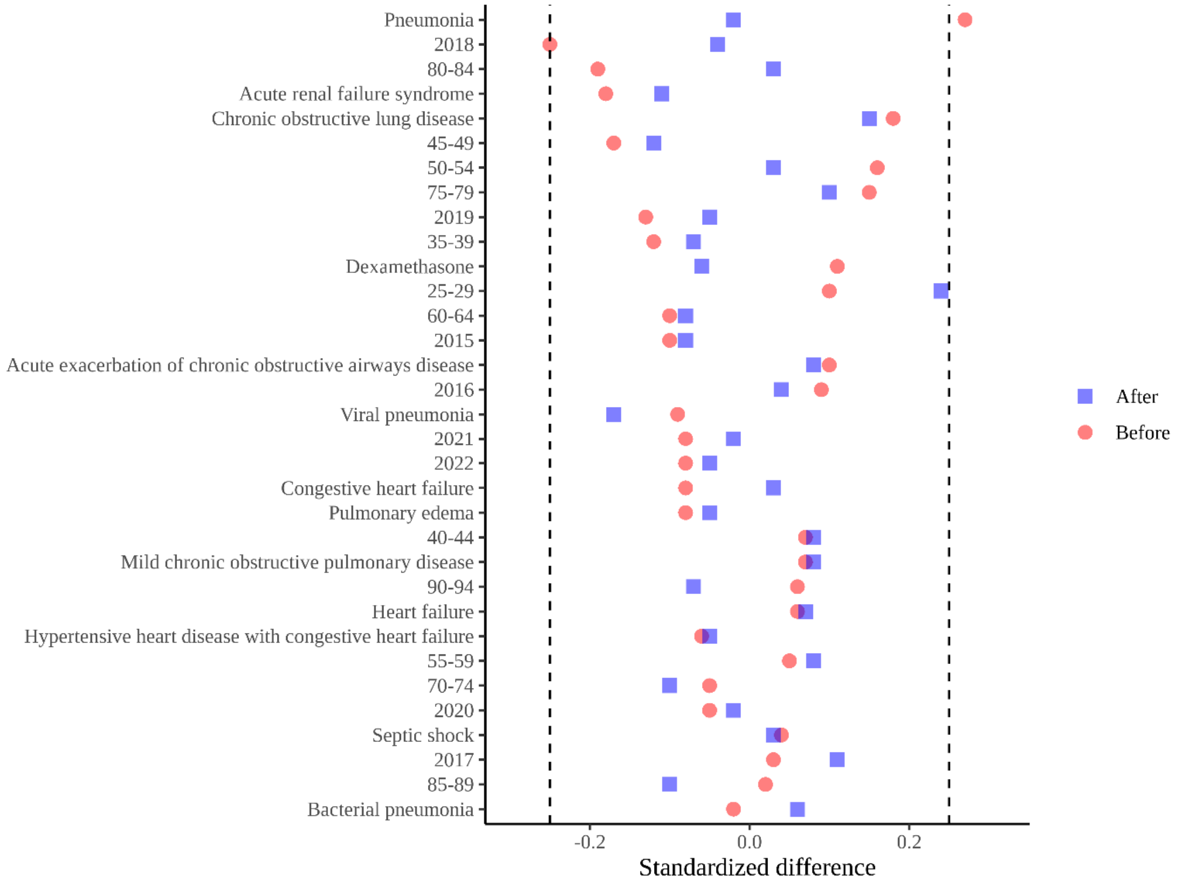

JCMJ, Jecheon Myongji Hospital.

Supplementary Figure S5B. Love plot of standardized mean differences before and after propensity score adjustment: aminopenicillin/β-lactamase inhibitor combinations vs third-generation cephalosporins (JCMJ)

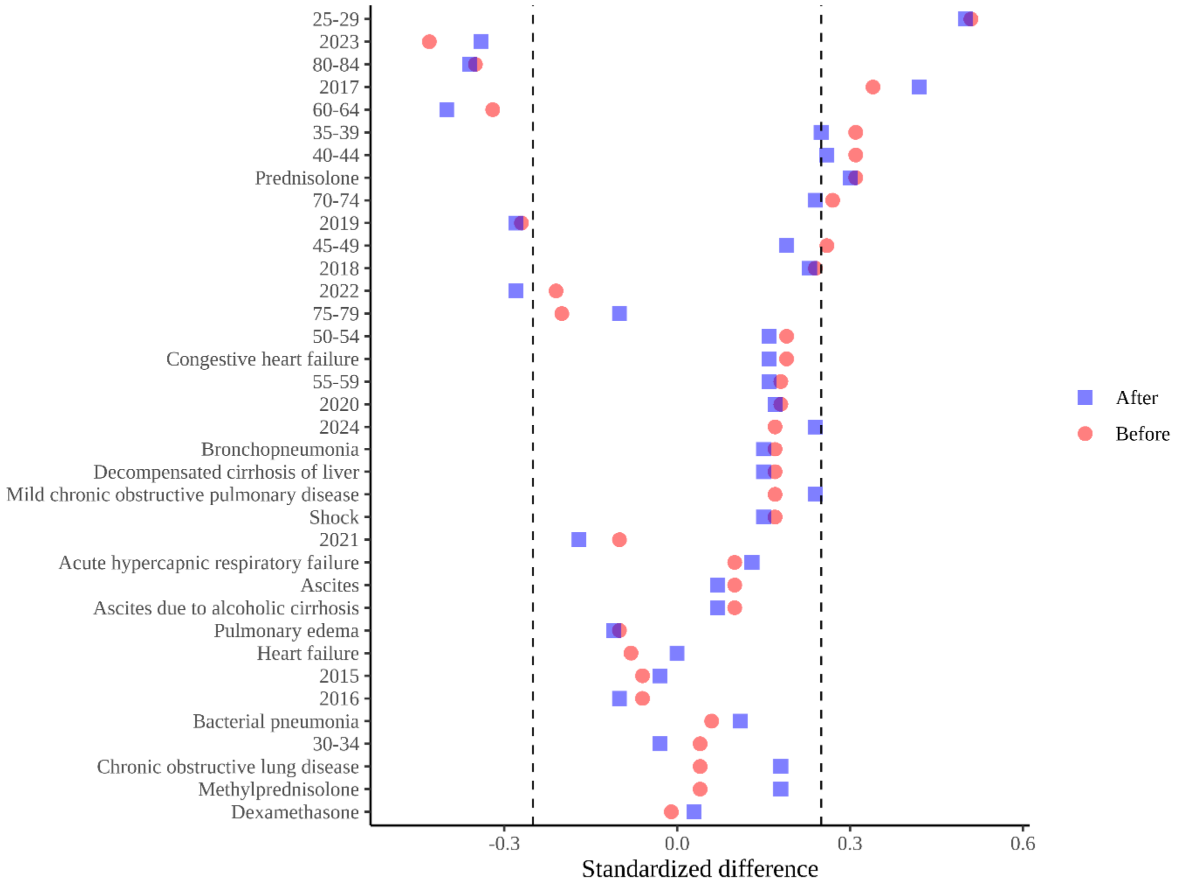

JCMJ, Jecheon Myongji Hospital.

Supplementary Figure S6A. Love plot of standardized mean differences before and after propensity score adjustment: fluoroquinolones vs third-generation cephalosporins (KDH)

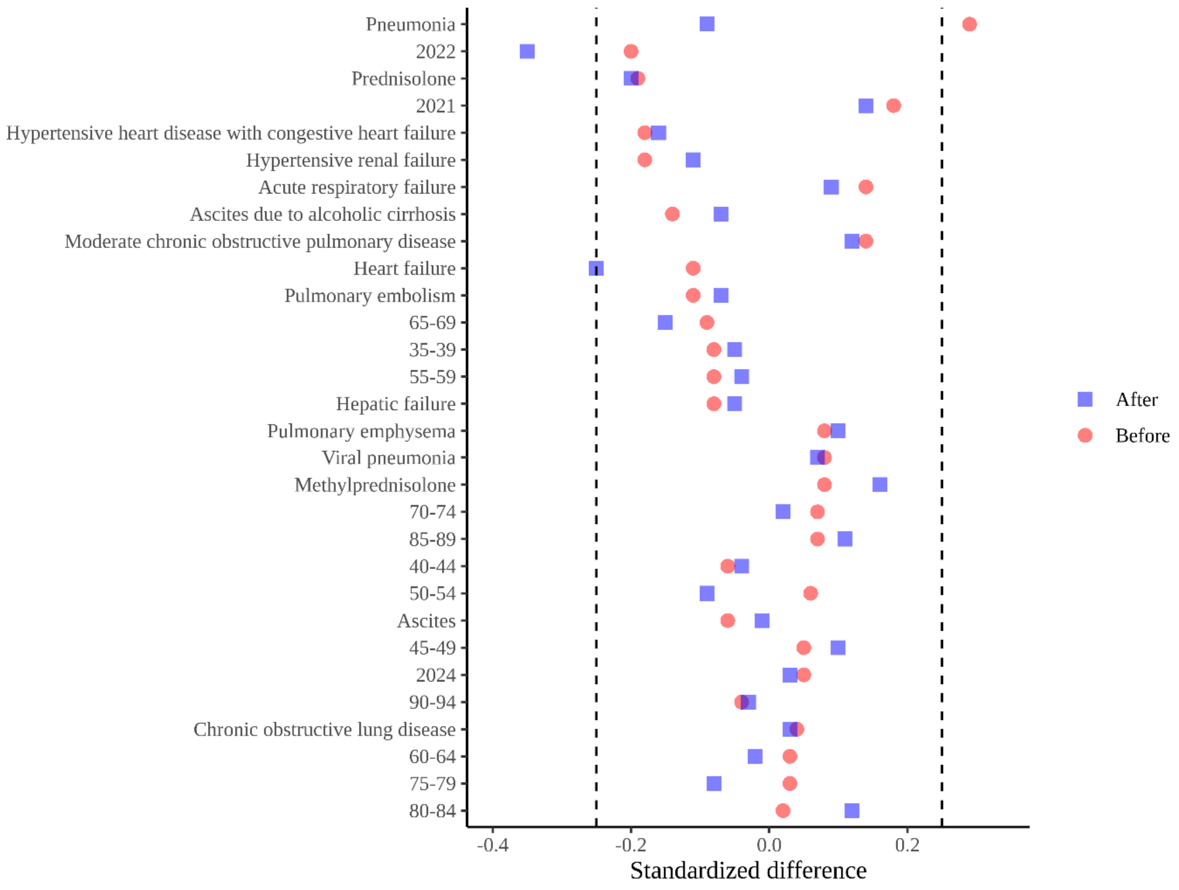

KDH, Kangdong Sacred Heart Hospital.

Supplementary Figure S6B. Love plot of standardized mean differences before and after propensity score adjustment: aminopenicillin/β-lactamase inhibitor combinations vs third-generation cephalosporins (KDH)

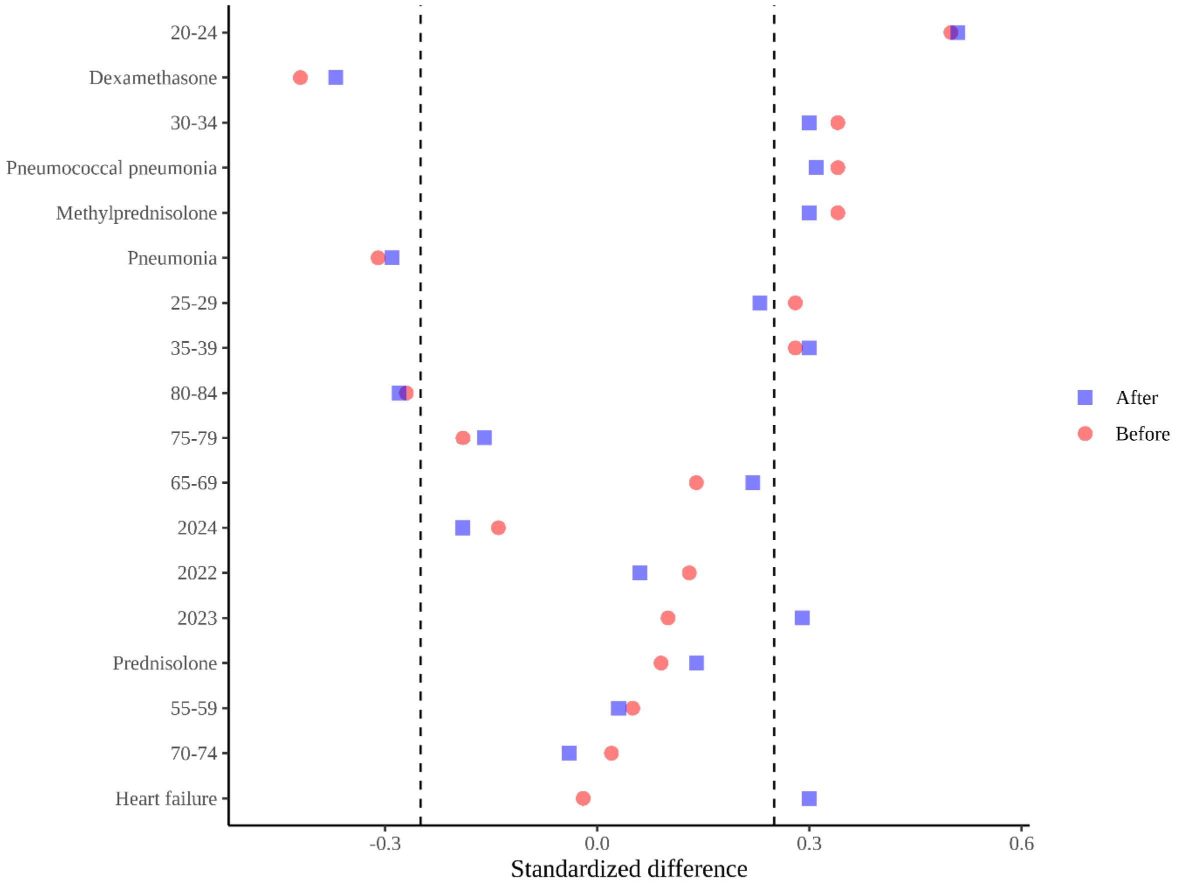

KDH, Kangdong Sacred Heart Hospital.

Supplementary Figure S7A. Love plot of standardized mean differences before and after propensity score adjustment: fluoroquinolones vs third-generation cephalosporins (KHMC)

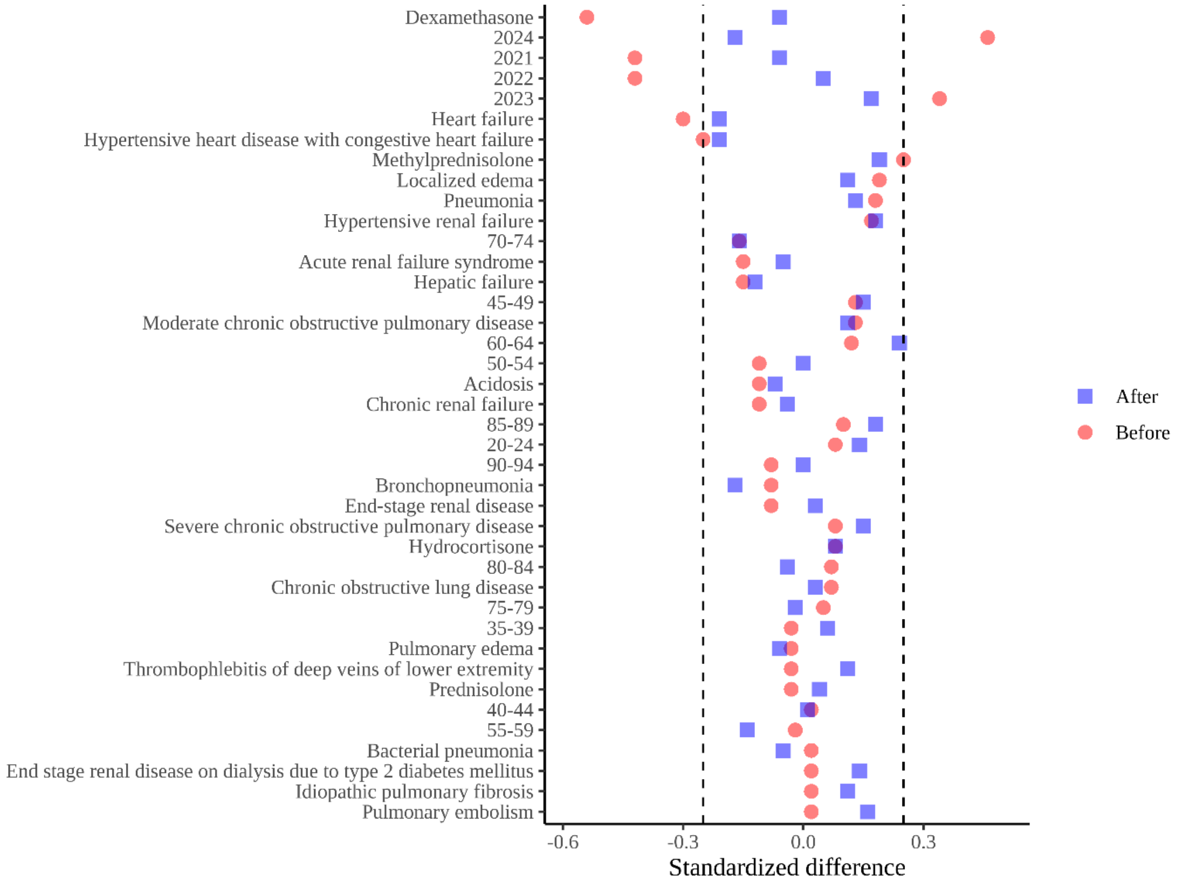

KHMC, Kyunghee University Medical Center.

Supplementary Figure S7B. Love plot of standardized mean differences before and after propensity score adjustment: aminopenicillin/β-lactamase inhibitor combinations vs third-generation cephalosporins (KHMC)

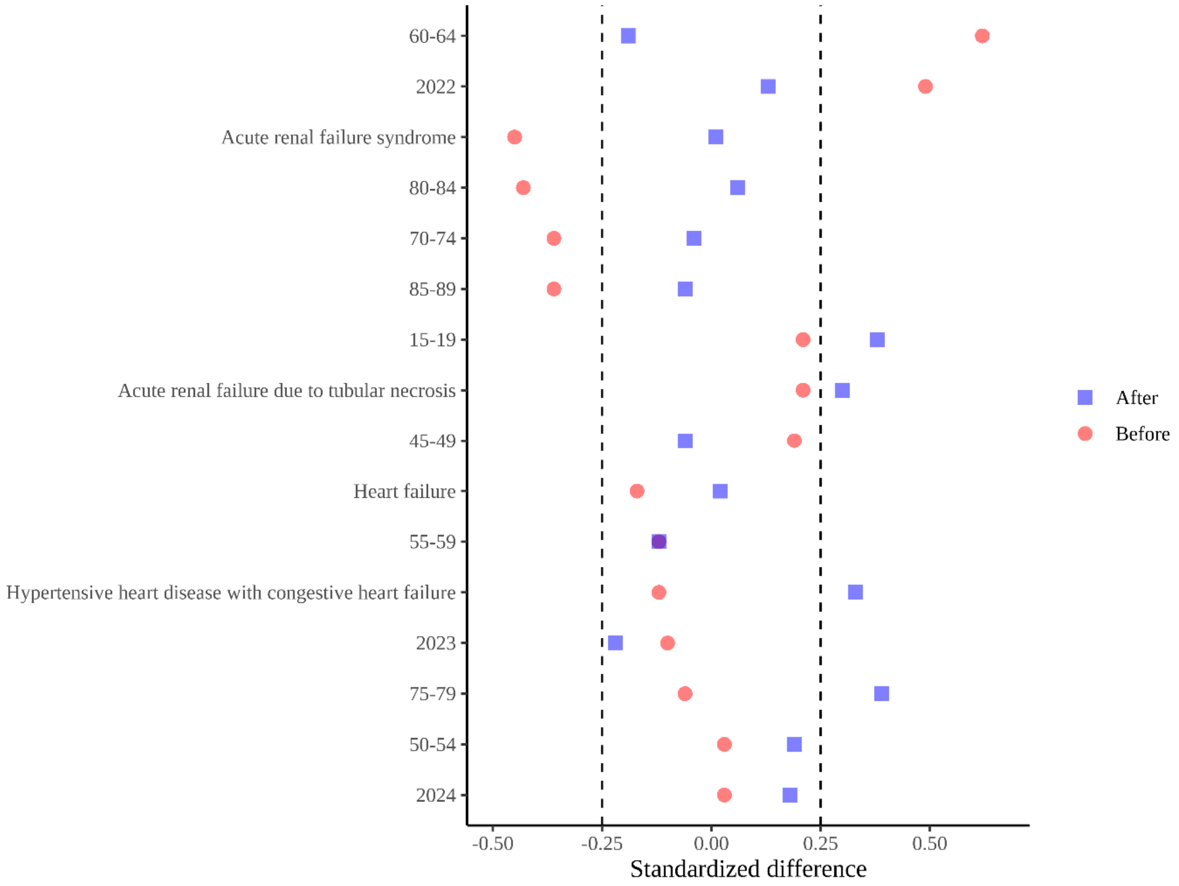

KHMC, Kyunghee University Medical Center.

Supplementary Figure S8A. Love plot of standardized mean differences before and after propensity score adjustment: fluoroquinolones vs third-generation cephalosporins (KWMC)

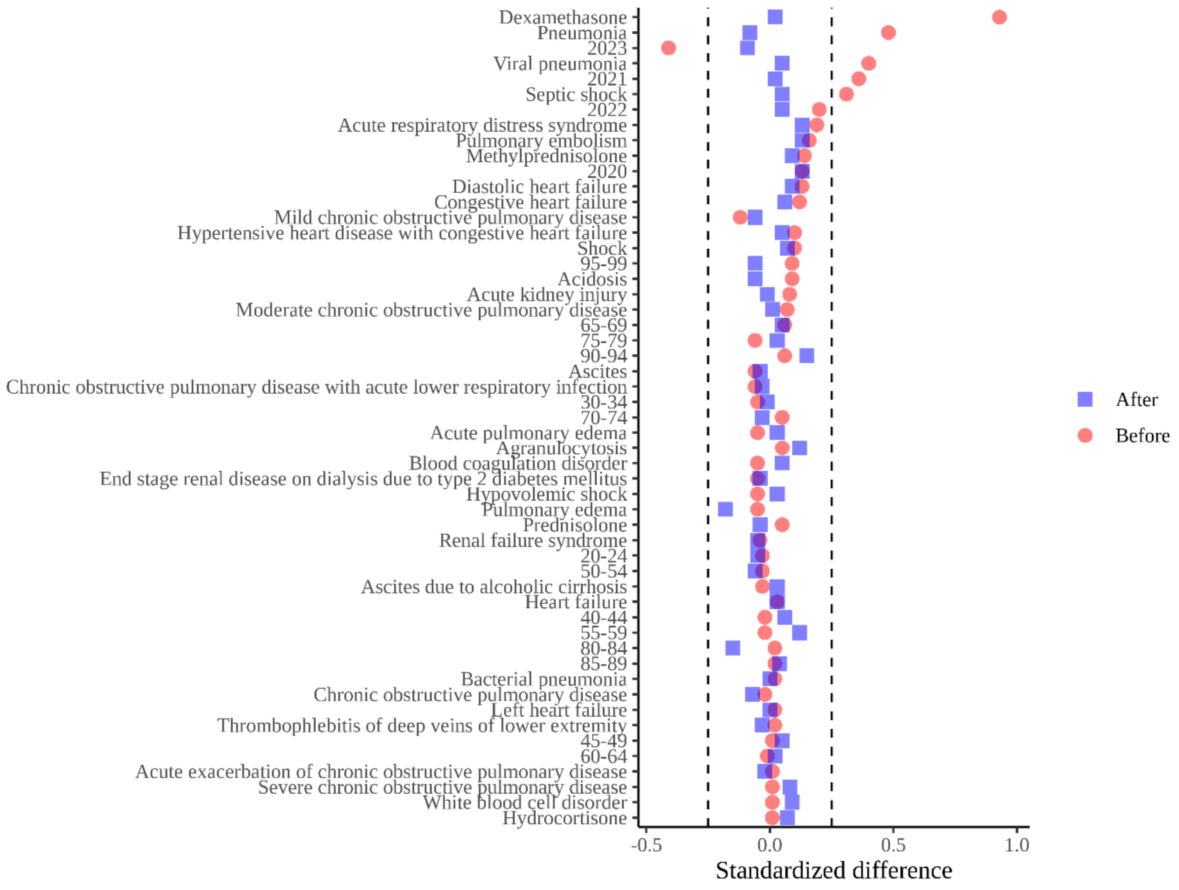

KWMC, Gangwon National University Medical Center.

Supplementary Figure S8B. Love plot of standardized mean differences before and after propensity score adjustment: aminopenicillin/β-lactamase inhibitor combinations vs third-generation cephalosporins (KWMC)

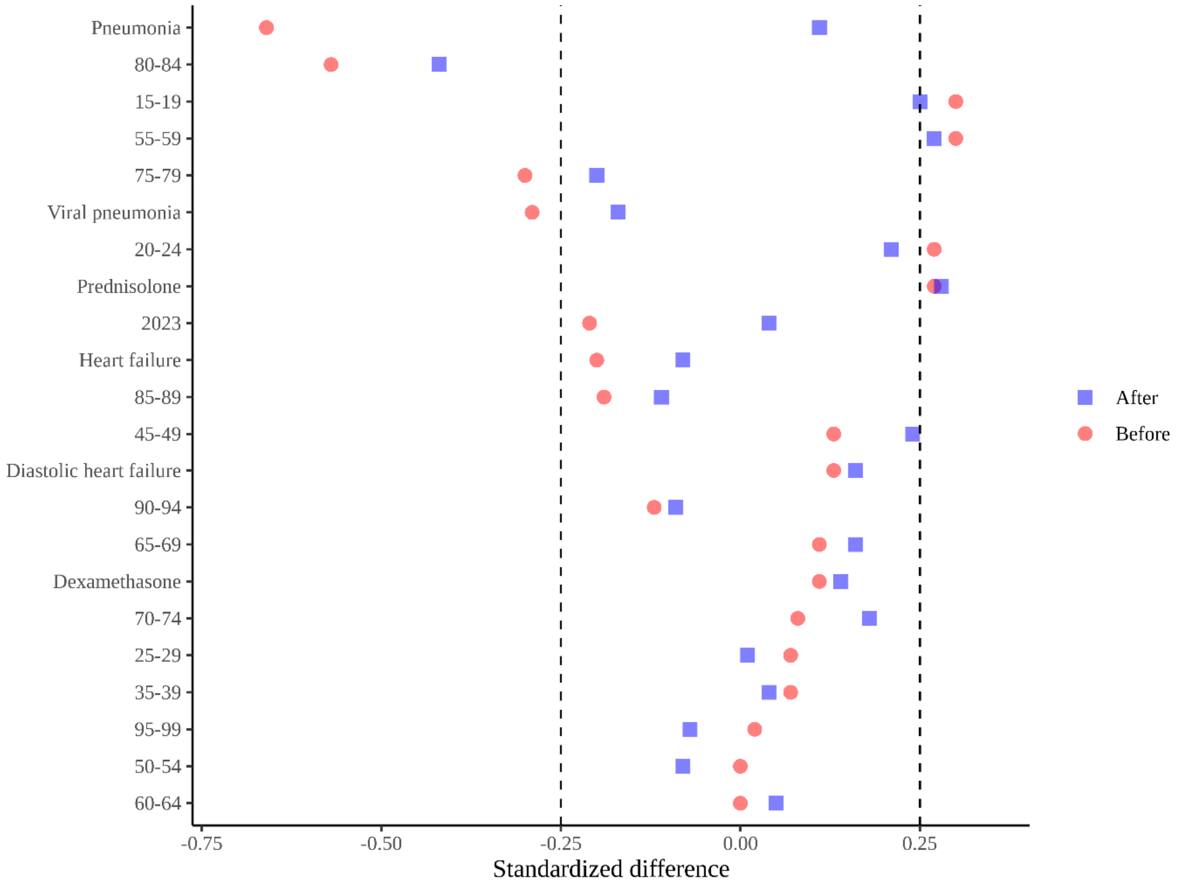

KWMC, Gangwon National University Medical Center.

Supplementary Figure S9A. Love plot of standardized mean differences before and after propensity score adjustment: fluoroquinolones vs third-generation cephalosporins (MJH)

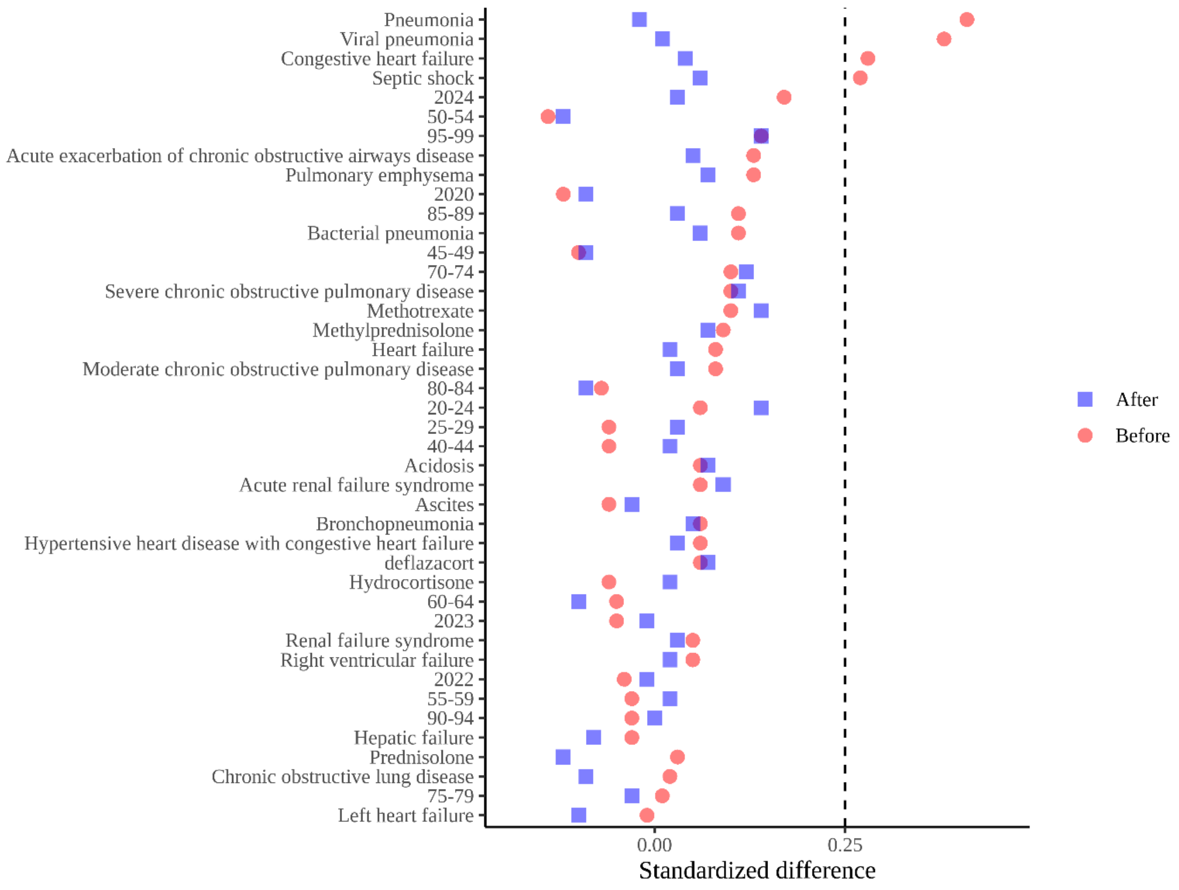

MJH, Myongji Hospital.

Supplementary Figure S9B. Love plot of standardized mean differences before and after propensity score adjustment: aminopenicillin/β-lactamase inhibitor combinations vs third-generation cephalosporins (MJH)

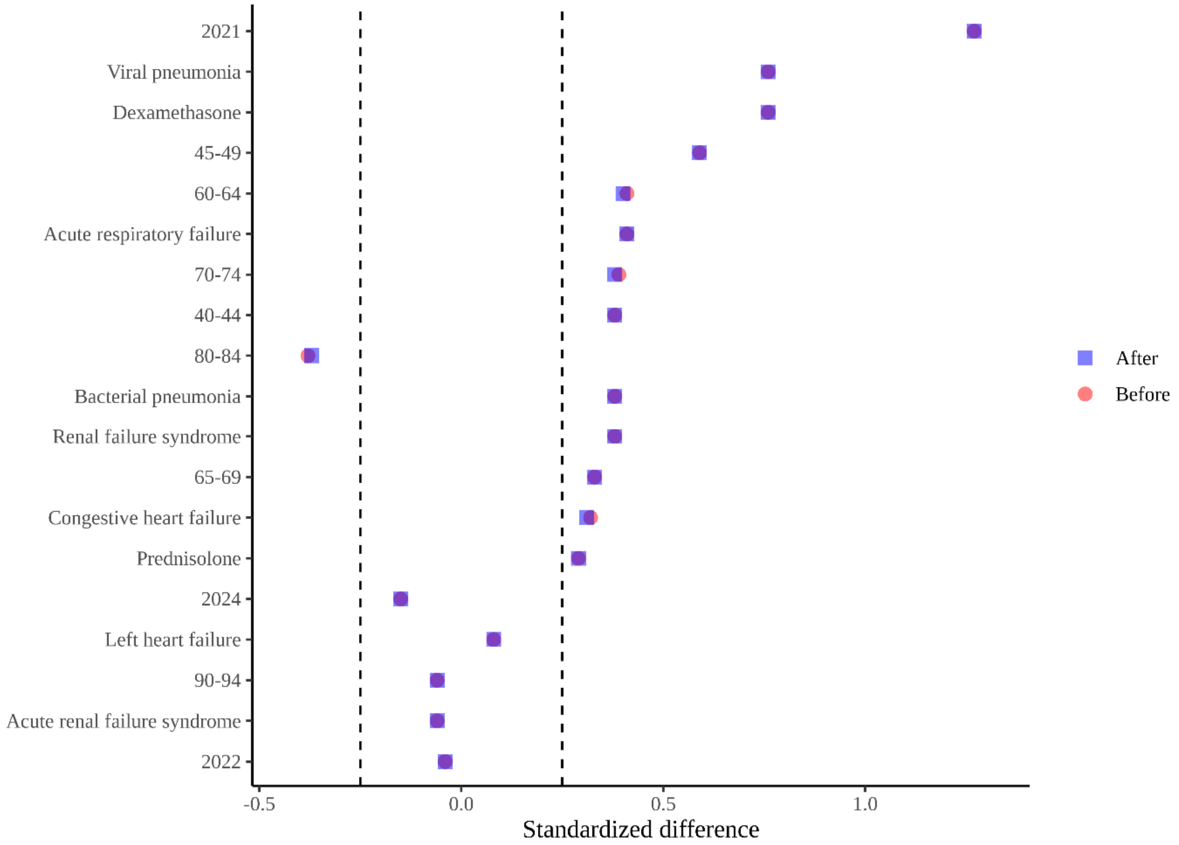

MJH, Myongji Hospital.

Supplementary Figure S10A. Love plot of standardized mean differences before and after propensity score adjustment: fluoroquinolones vs third-generation cephalosporins (SCHBC)

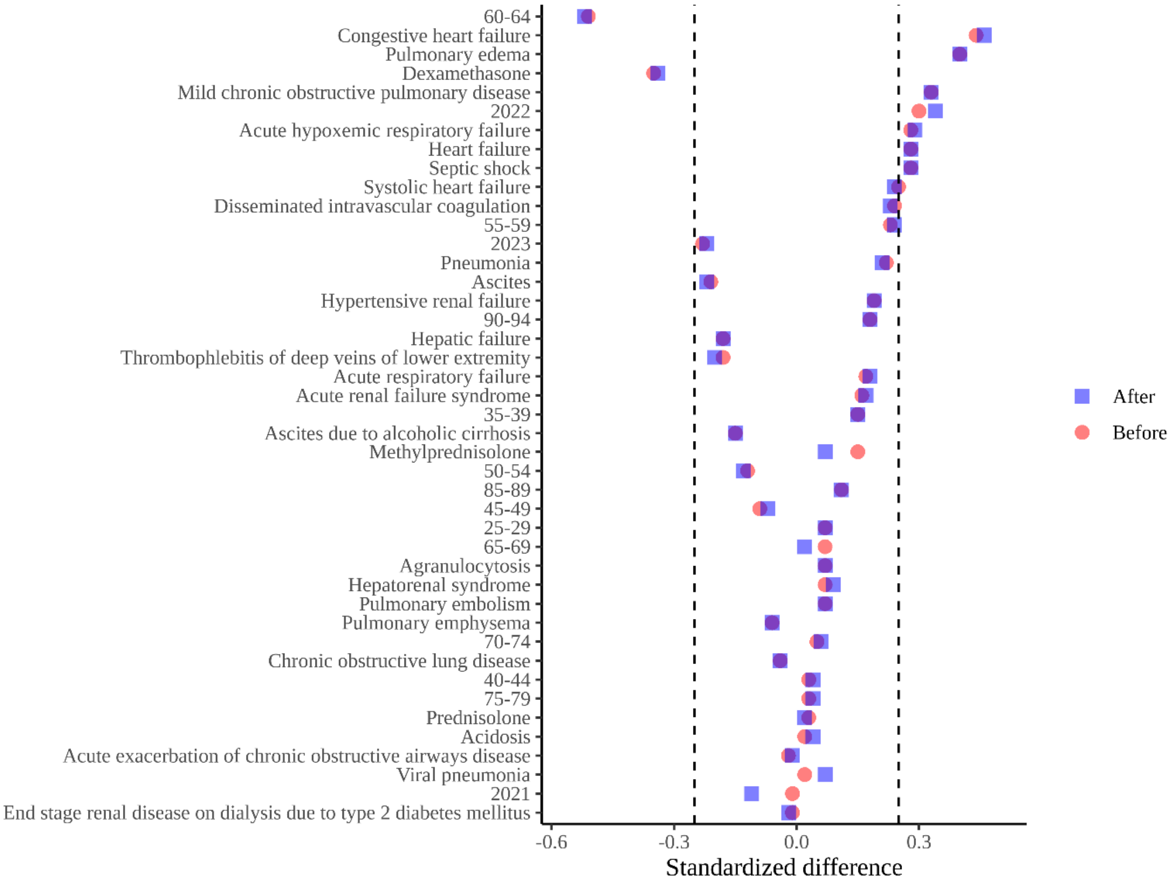

SCHBC, Soonchunhyang University Hospital Bucheon Center.

Supplementary Figure S11A. Love plot of standardized mean differences before and after propensity score adjustment: fluoroquinolones vs third-generation cephalosporins (SCHCA)

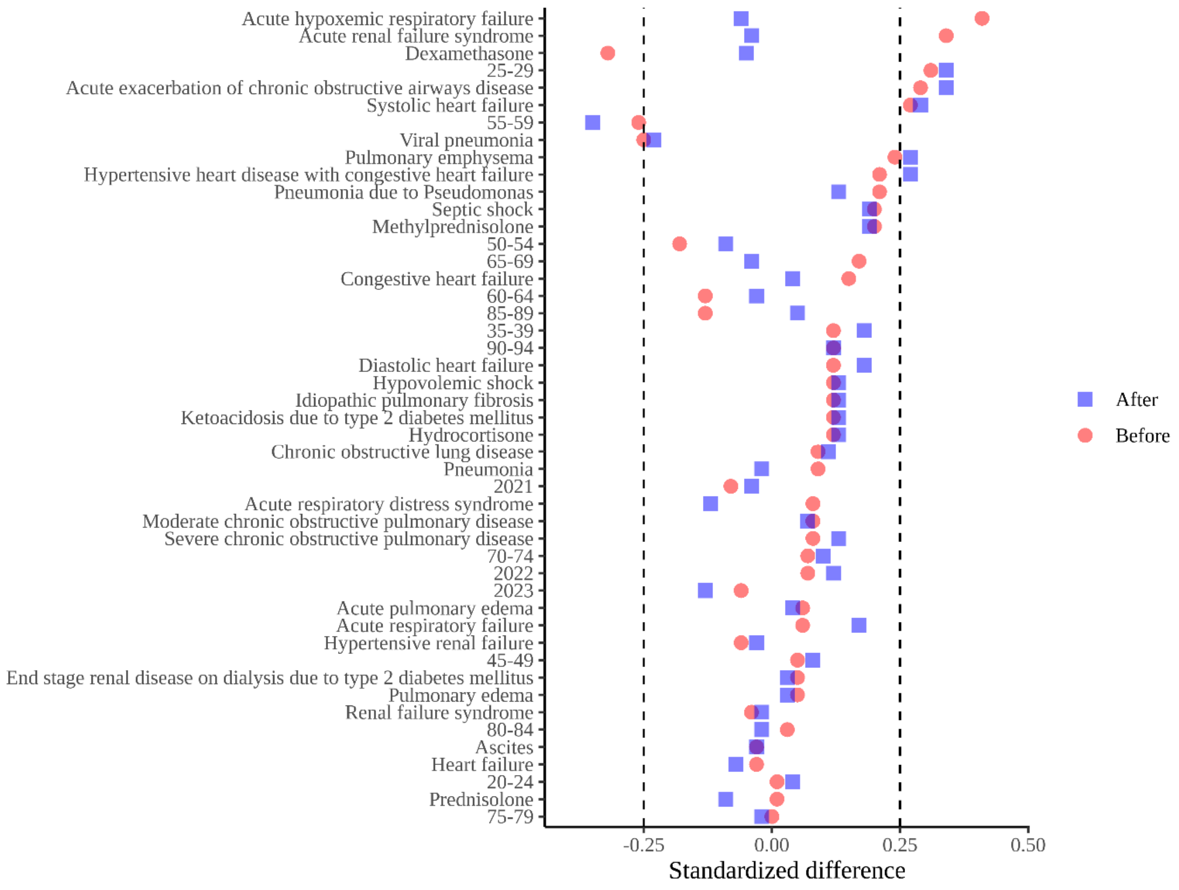

SCHCA, Soonchunhyang University Hospital Cheonan Center.

Supplementary Figure S12A. Love plot of standardized mean differences before and after propensity score adjustment: fluoroquinolones vs third-generation cephalosporins (WKUH)

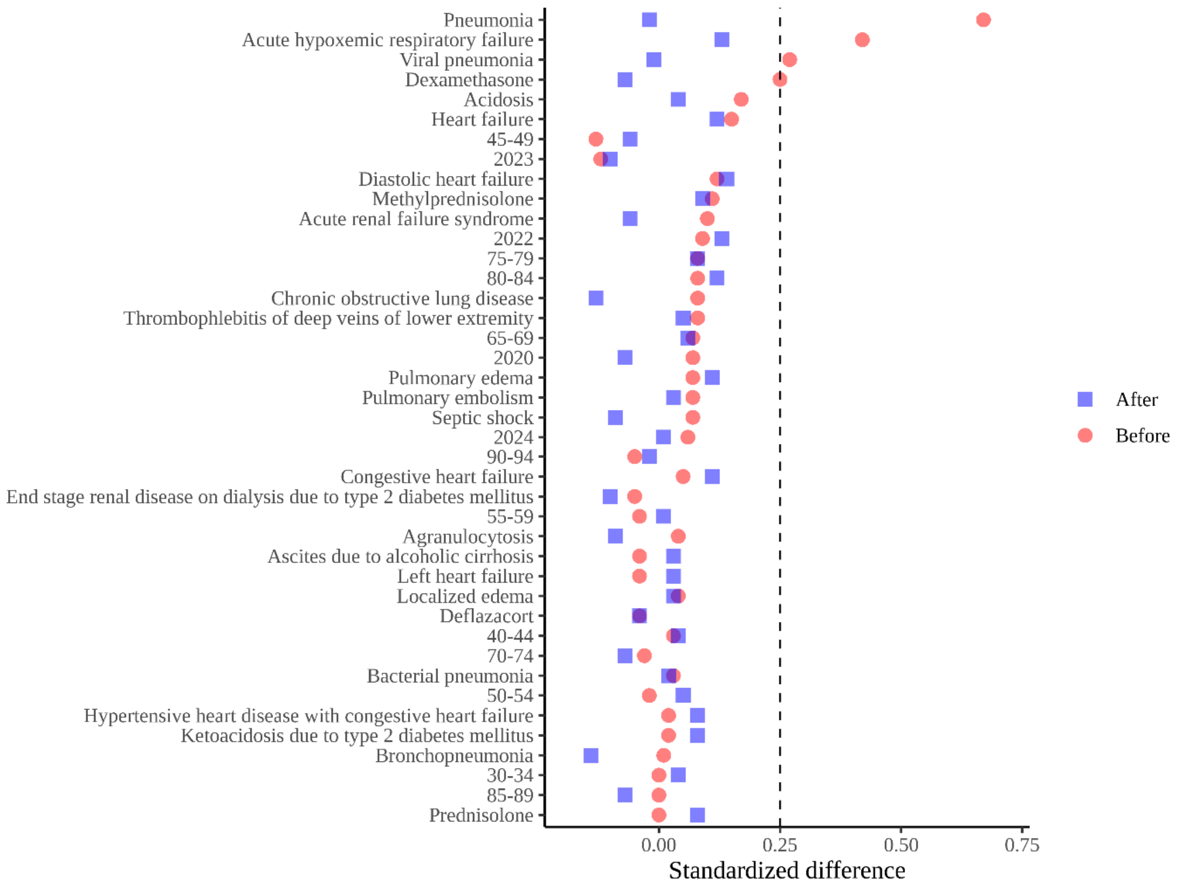

WKUH, Wonkwang University Hospital.

Supplementary Figure S12B. Love plot of standardized mean differences before and after propensity score adjustment: aminopenicillin/β-lactamase inhibitor combinations vs third-generation cephalosporins (WKUH)

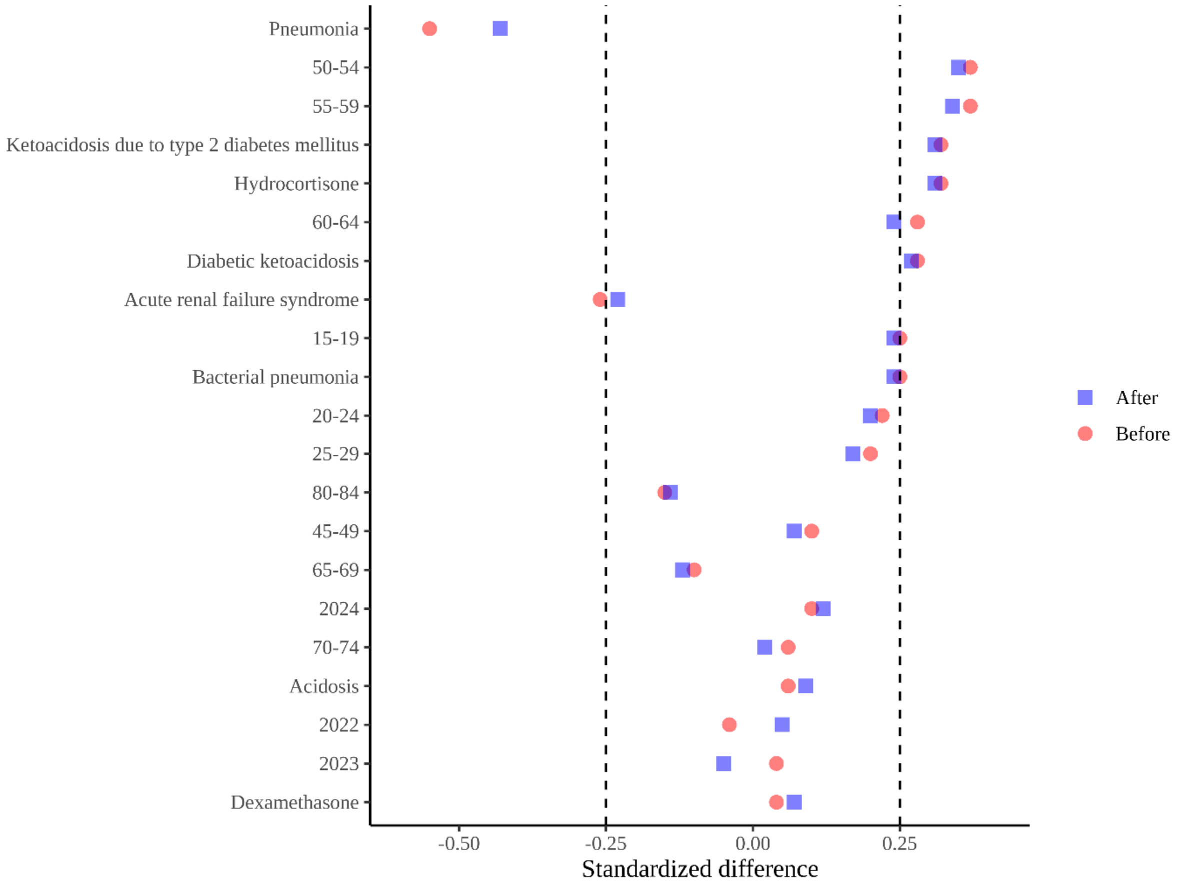

WKUH, Wonkwang University Hospital.

Supplementary Figure S13A. All-Cause mortality by antibiotic class: Kaplan–Meier analysis after propensity score matching (3GCs vs FQs)

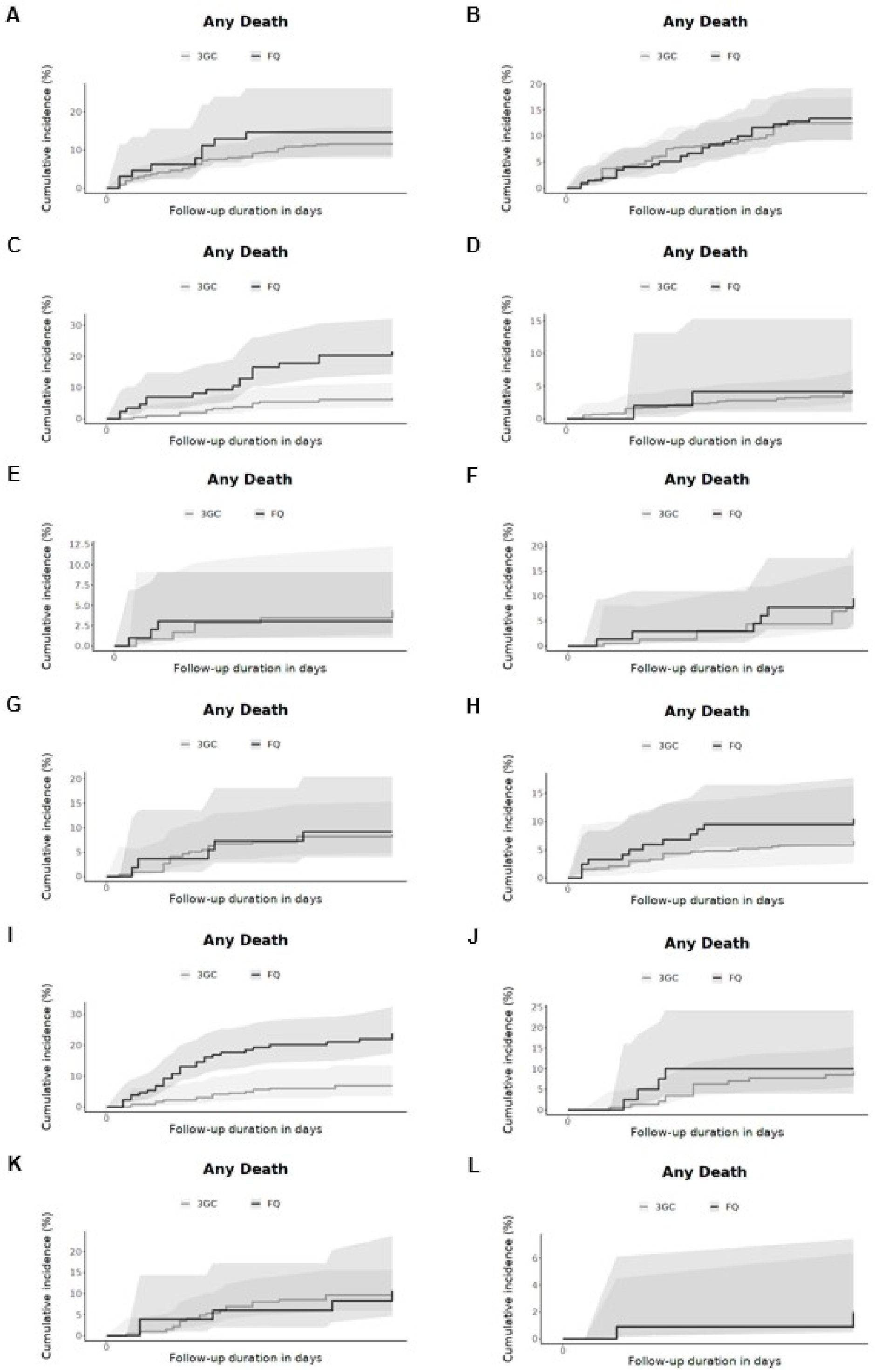

(A) AUMC. (B) EUMC. (C) GNUH. (D) ISH. (E) JCMJ. (F) KDH. (G) KHMC. (H) KWMC. (I) MJH. (J) SCHCA. (K) SCHBC. (L) WKUH. 3GCs, Third-generation cephalosporins; AUMC, Ajou University Medical Center; EUMC, Ewha Womans University Medical Center; FQ, Fluoroquinolones; GNUH, Gyeongsang National University Hospital; ISH, International St. Mary's Hospital; JCMJ, Jecheon Myongji Hospital; KDH, Kangdong Sacred Heart Hospital; KHMC, Kyunghee University Medical Center; KWMC, Kangwon National University Medical Center; MJH, Myongji Hospital; SCHCA, Soonchunhyang University Hospital Cheonan Center; SCHBC, Soonchunhyang University Hospital Bucheon Center; WKUH, Wonkwang University Hospital.

Supplementary Figure S13B. Composite outcome by antibiotic class: Kaplan–Meier analysis after propensity score matching (3GCs vs FQs)

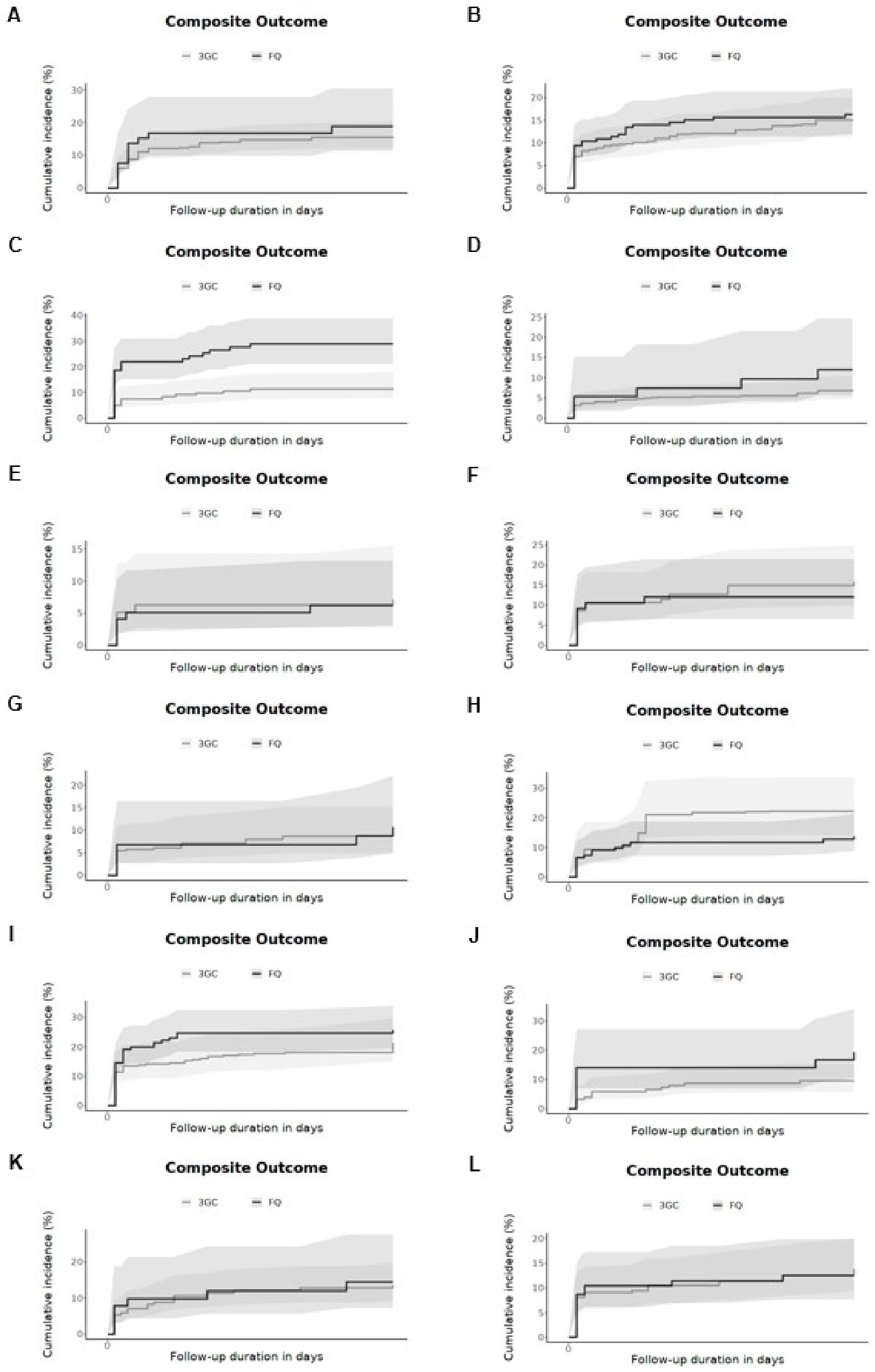

(A) AUMC. (B) EUMC. (C) GNUH. (D) ISH. (E) JCMJ. (F) KDH. (G) KHMC. (H) KWMC. (I) MJH. (J) SCHCA. (K) SCHBC. (L) WKUH. 3GCs, Third-generation cephalosporins; AUMC, Ajou University Medical Center; EUMC, Ewha Womans University Medical Center; FQ, Fluoroquinolones; GNUH, Gyeongsang National University Hospital; ISH, International St. Mary's Hospital; JCMJ, Jecheon Myongji Hospital; KDH, Kangdong Sacred Heart Hospital; KHMC, Kyunghee University Medical Center; KWMC, Kangwon National University Medical Center; MJH, Myongji Hospital; SCHCA, Soonchunhyang University Hospital Cheonan Center; SCHBC, Soonchunhyang University Hospital Bucheon Center; WKUH, Wonkwang University Hospital.

Supplementary Figure S14A. All-Cause mortality by antibiotic class: Kaplan–Meier analysis after propensity score matching (3GCs vs PEN-BLis)

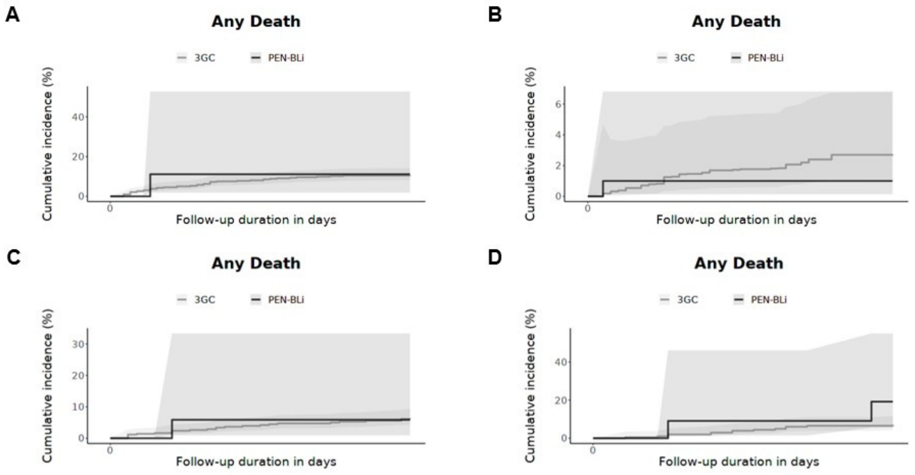

(A) AUMC. (B) EUMC. (C) ISH. (D) MJH. 3GCs, Third-generation cephalosporins; AUMC, Ajou University Medical Center; EUMC, Ewha Womans University Medical Center; ISH, International St. Mary's Hospital; MJH, Myongji Hospital; PEN-BLi, aminopenicillin/β-lactamase inhibitor combinations.

Supplementary Figure S14B. Composite outcome by antibiotic class: Kaplan–Meier analysis after propensity score matching (3GCs vs PEN-BLis)

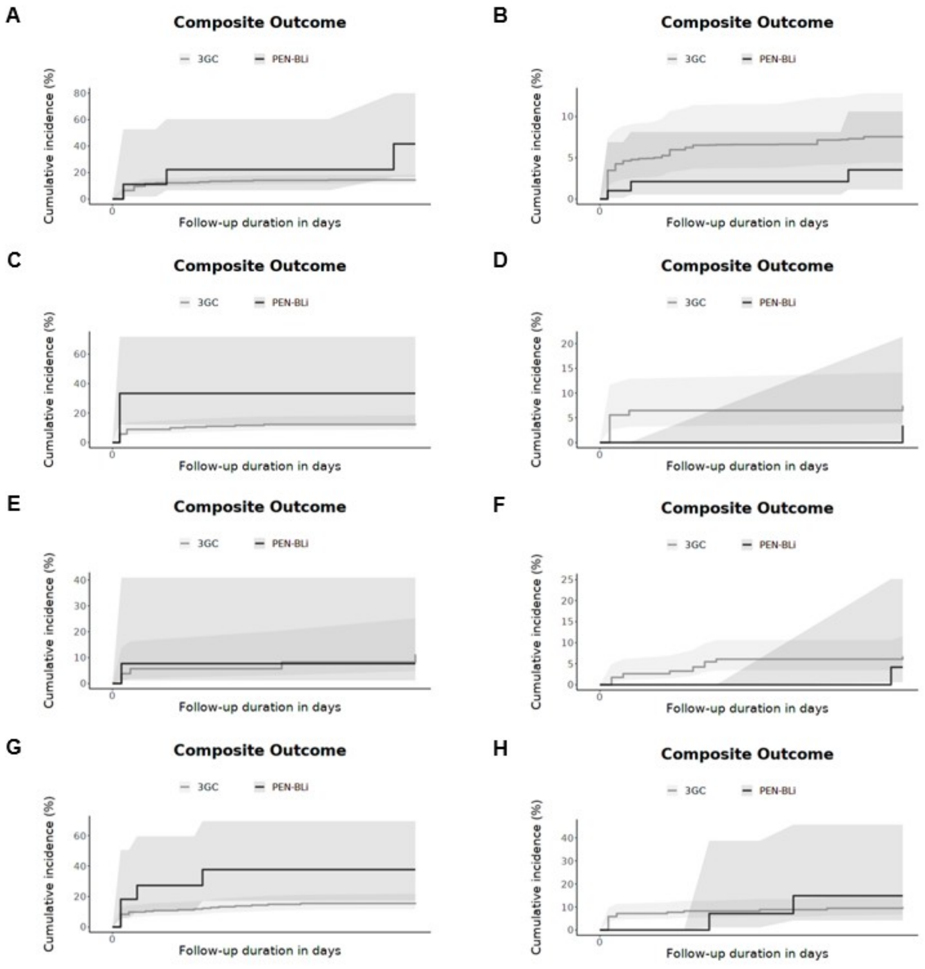

(A) AUMC. (B) EUMC. (C) GNUH. (D) JCMJ. (E) KDH. (F) KWMC (G) MJH. (H) WKUH. 3GCs, Third-generation cephalosporins; AUMC, Ajou University Medical Center; EUMC, Ewha Womans University Medical Center; GNUH, Gyeongsang National University Hospital; JCMJ, Jecheon Myongji Hospital; KDH, Kangdong Sacred Heart Hospital; KWMC, Kangwon National University Medical Center; MJH, Myongji Hospital; PEN-BLi, aminopenicillin/β-lactamase inhibitor combinations; WKUH, Wonkwang University Hospital.

Supplementary Figure S15A. Mechanical ventilation by antibiotic class: Kaplan–Meier analysis after propensity score matching (3GCs vs FQs)

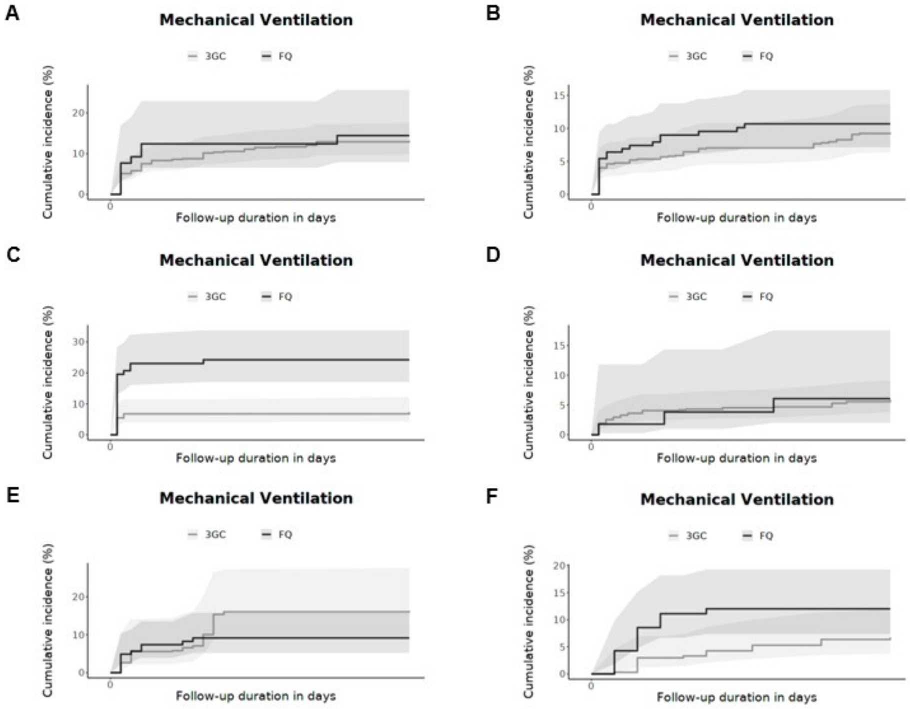

(A) AUMC. (B) EUMC. (C) GNUH. (D) ISH. (E) KWMC. (F) WKUH. 3GCs, Third-generation cephalosporins; AUMC, Ajou University Medical Center; EUMC, Ewha Womans University Medical Center; FQ, Fluoroquinolones; GNUH, Gyeongsang National University Hospital; ISH, International St. Mary's Hospital; KWMC, Kangwon National University Medical Center; WKUH, Wonkwang University Hospital.

Supplementary Figure S15B. ICU admission by antibiotic class: Kaplan–Meier analysis after propensity score matching (3GCs vs FQs)

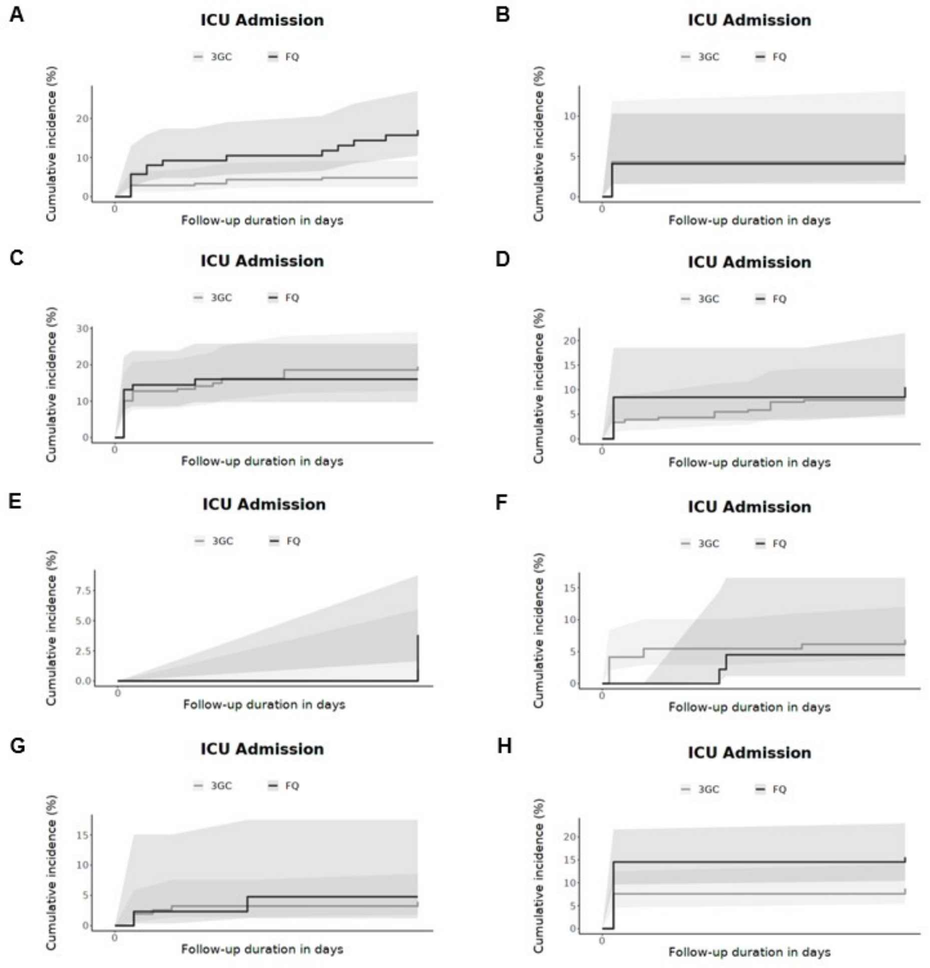

(A) GNUH. (B) JCMJ. (C) KDH. (D) KHMC. (E) MJH. (F) SCHCA. (G) SCHBC. (H) WKUH. 3GCs, Third-generation cephalosporins; FQ, Fluoroquinolones; GNUH, Gyeongsang National University Hospital; JCMJ, Jecheon Myongji Hospital; KDH, Kangdong Sacred Heart Hospital; KHMC, Kyunghee University Medical Center; MJH, Myongji Hospital; SCHCA, Soonchunhyang University Hospital Cheonan Center; SCHBC, Soonchunhyang University Hospital Bucheon Center; WKUH, Wonkwang University Hospital.

Supplementary Figure S15C. Vasopressor use by antibiotic class: Kaplan–Meier analysis after propensity score matching (3GCs vs FQs)

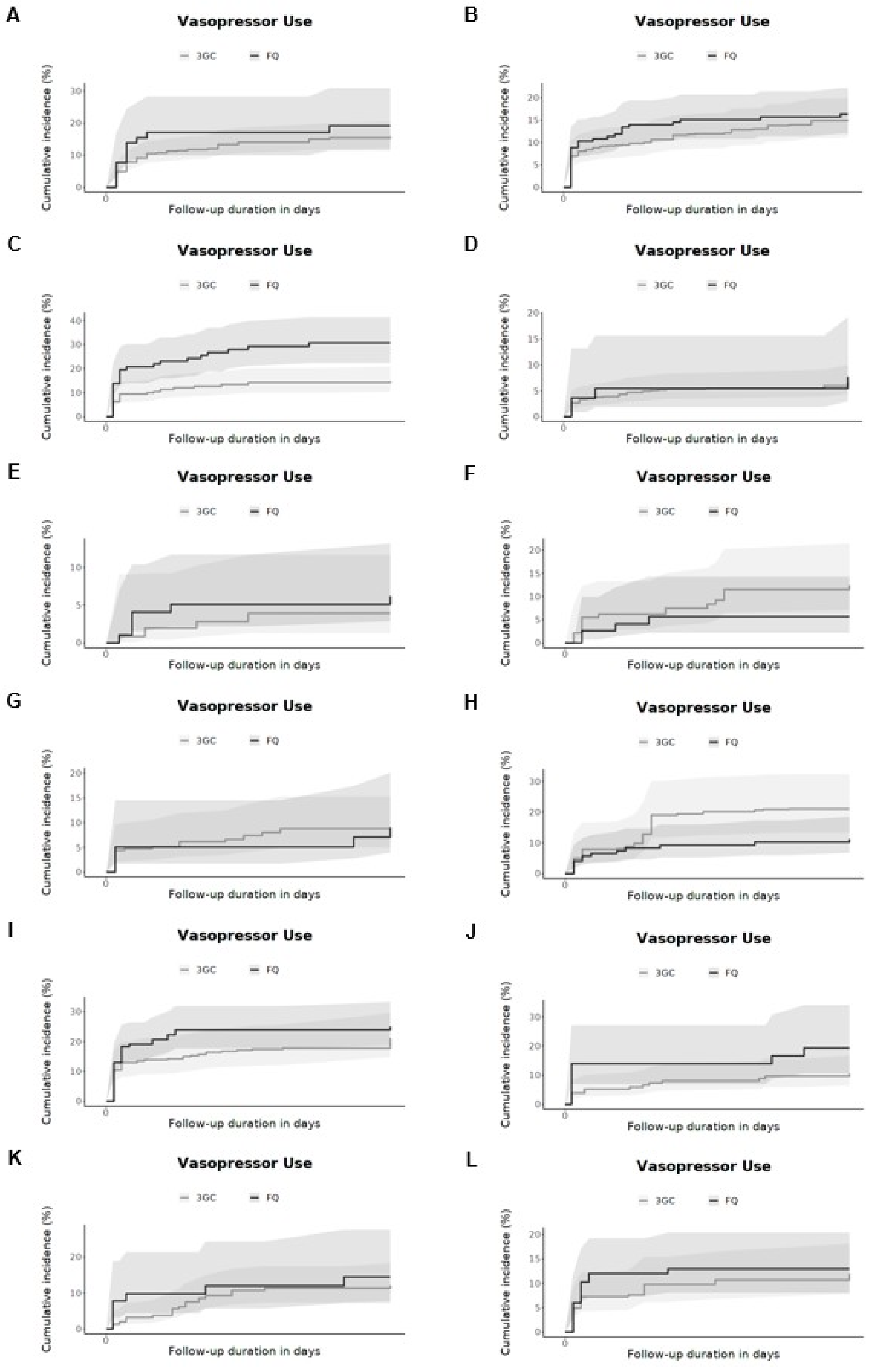

(A) AUMC. (B) EUMC. (C) GNUH. (D) ISH. (E) JCMJ. (F) KDH. (G) KHMC. (H) KWMC. (I) MJH. (J) SCHCA. (K) SCHBC. (L) WKUH. 3GCs, Third-generation cephalosporins; AUMC, Ajou University Medical Center; EUMC, Ewha Womans University Medical Center; FQ, Fluoroquinolones; GNUH, Gyeongsang National University Hospital; ISH, International St. Mary's Hospital; JCMJ, Jecheon Myongji Hospital; KDH, Kangdong Sacred Heart Hospital; KHMC, Kyunghee University Medical Center; KWMC, Kangwon National University Medical Center; MJH, Myongji Hospital; SCHCA, Soonchunhyang University Hospital Cheonan Center; SCHBC, Soonchunhyang University Hospital Bucheon Center; WKUH, Wonkwang University Hospital.

Supplementary Figure S16A. Mechanical ventilation by antibiotic class: Kaplan–Meier analysis after propensity score matching (3GCs vs PEN-BLis)

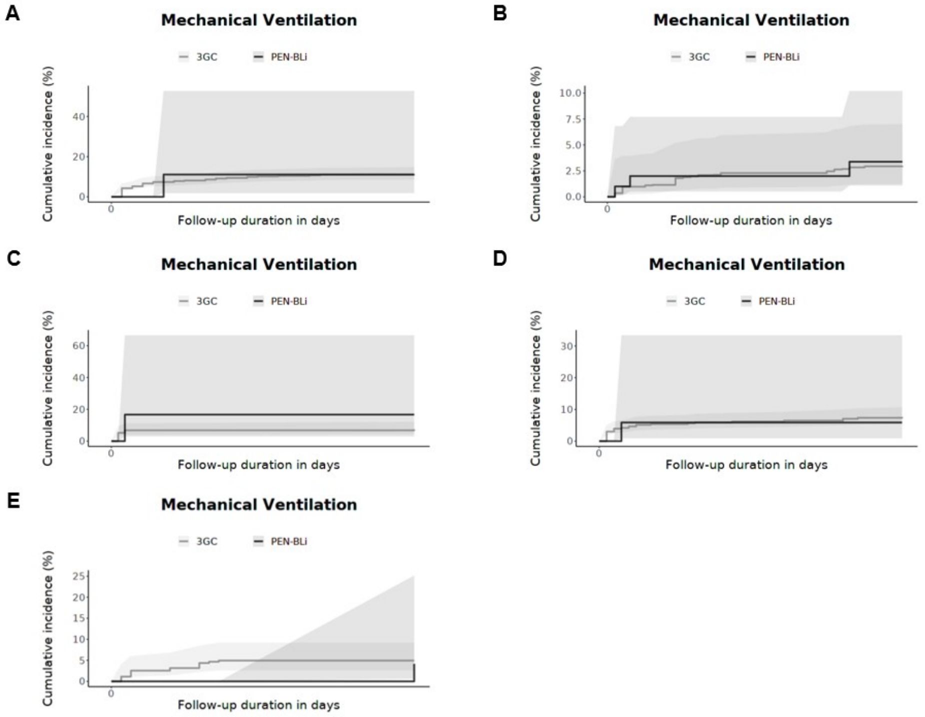

(A) AUMC. (B) EUMC. (C) GNUH. (D) ISH. (E) KWMC. 3GCs, Third-generation cephalosporins; AUMC, Ajou University Medical Center; EUMC, Ewha Womans University Medical Center; GNUH, Gyeongsang National University Hospital; ISH, International St. Mary's Hospital; KWMC, Kangwon National University Medical Center; PEN-BLi, aminopenicillin/β-lactamase inhibitor combinations.

Supplementary Figure S16B. Vasopressor use by antibiotic class: Kaplan–Meier analysis after propensity score matching (3GCs vs PEN-BLis)

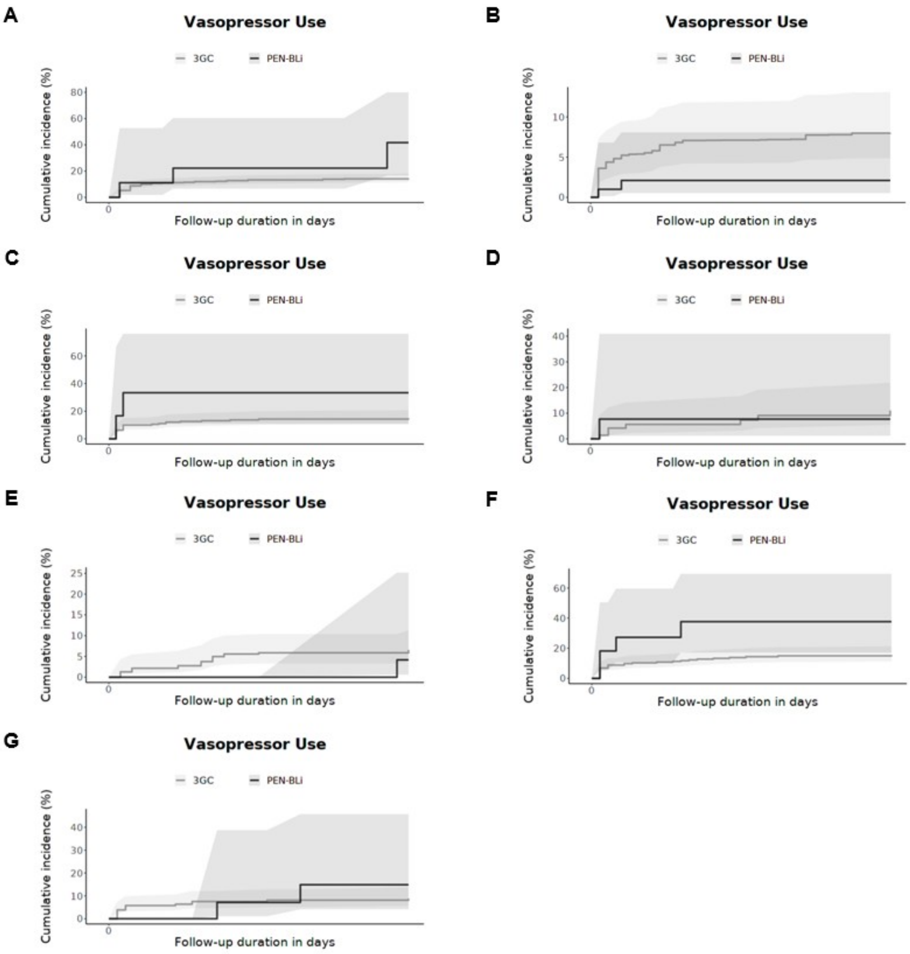

(A) AUMC. (B) EUMC. (C) GNUH. (D) KDH. (E) KWMC. (F) MJH. (E) WKUH. 3GCs, Third-generation cephalosporins; AUMC, Ajou University Medical Center; EUMC, Ewha Womans University Medical Center; GNUH, Gyeongsang National University Hospital; KDH, Kangdong Sacred Heart Hospital; KWMC, Kangwon National University Medical Center; MJH, Myongji Hospital; PEN-BLi, aminopenicillin/β-lactamase inhibitor combinations; WKUH, Wonkwang University Hospital.

Supplementary Figure S17A. Comparative risks of primary outcomes between fluoroquinolones and 3GCs among hospitalized COVID-19 patients: sensitivity analysis with expanded severity covariates

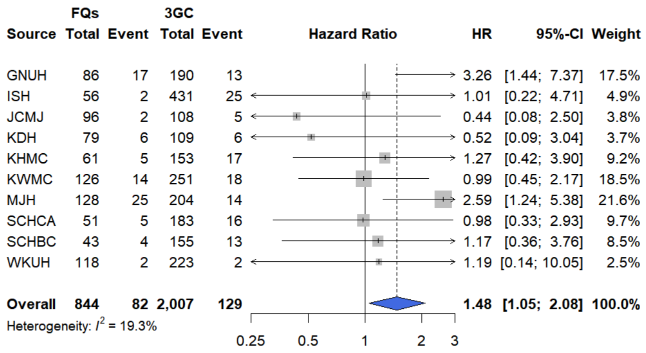

(a)

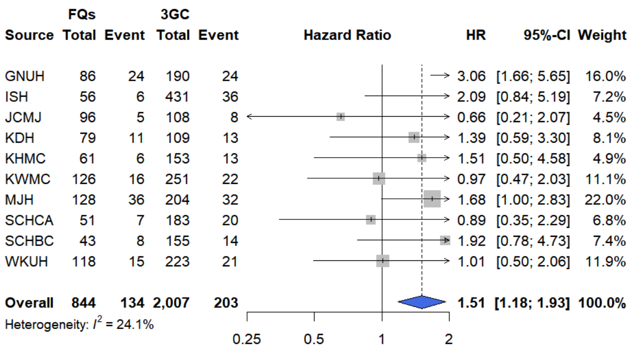

(b)

(a) All-cause in-hospital mortality. (b) Composite outcome. The number of events and total patients in each treatment group are shown alongside site-specific hazard ratios (HRs). The HRs were calibrated based on the empirical null distribution derived from negative control outcomes to account for systematic bias. The size of the data marker indicates the weight of the study. Error bars indicate 95% confidence intervals (CIs). 3GCs, Third-generation cephalosporins; FQs, Fluoroquinolones; GNUH, Gyeongsang National University Hospital; ISH, International St. Mary's Hospital; JCMJ, Jecheon Myongji Hospital; KDH, Kangdong Sacred Heart Hospital; KHMC, Kyunghee University Medical Center; KWMC, Kangwon National University Medical Center; MJH, Myongji Hospital; SCHCA, Soonchunhyang University Hospital Cheonan Center; SCHBC, Soonchunhyang University Hospital Bucheon Center; WKUH, Wonkwang University Hospital.

Supplementary Figure S17B. Comparative risks of primary outcomes between PEN–BLis and 3GCs among hospitalized COVID-19 patients: sensitivity analysis with expanded severity covariates

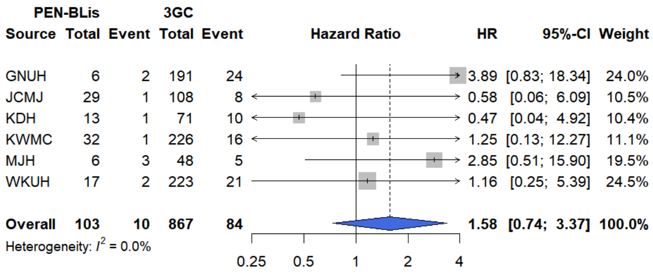

Composite outcome. The number of events and total patients in each treatment group are shown alongside site-specific hazard ratios (HRs). The HRs were calibrated based on the empirical null distribution derived from negative control outcomes to account for systematic bias. The size of the data marker indicates the weight of the study. Error bars indicate 95% confidence intervals (CIs). 3GCs, Third-generation cephalosporins; GNUH, Gyeongsang National University Hospital; JCMJ, Jecheon Myongji Hospital; KDH, Kangdong Sacred Heart Hospital; KWMC, Kangwon National University Medical Center; MJH, Myongji Hospital; PEN–BLis, aminopenicillin/β-lactamase inhibitor combinations; WKUH, Wonkwang University Hospital.

Supplementary Figure S18A. Comparative risks of secondary outcomes between fluoroquinolones and 3GCs among hospitalized COVID-19 patients: sensitivity analysis with expanded severity covariates

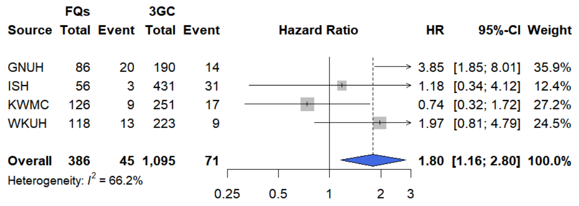

(a)

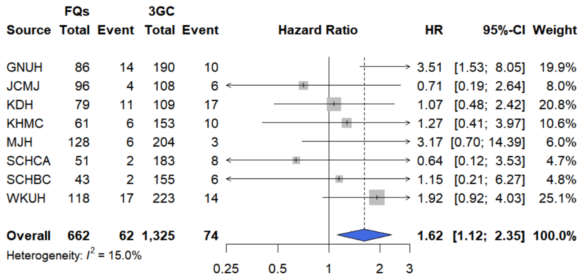

(b)

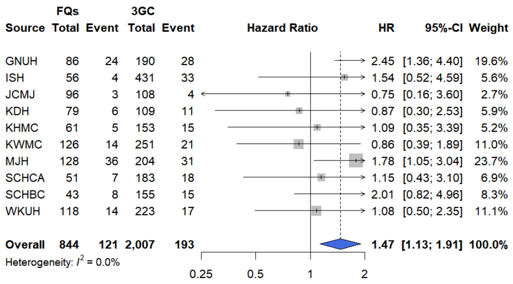

(c)

(a) Mechanical ventilation. (b) Intensive Care Unit (ICU) admission. (c) Vasopressor use. The number of events and total patients in each treatment group are shown alongside site-specific hazard ratios (HRs). The HRs were calibrated based on the empirical null distribution derived from negative control outcomes to account for systematic bias. The size of the data marker indicates the weight of the study. Error bars indicate 95% confidence intervals (CIs). 3GCs, Third-generation cephalosporins; FQs, Fluoroquinolones; GNUH, Gyeongsang National University Hospital; ISH, International St. Mary's Hospital; JCMJ, Jecheon Myongji Hospital; KDH, Kangdong Sacred Heart Hospital; KHMC, Kyunghee University Medical Center; KWMC, Kangwon National University Medical Center; MJH, Myongji Hospital; SCHCA, Soonchunhyang University Hospital Cheonan Center; SCHBC, Soonchunhyang University Hospital Bucheon Center; WKUH, Wonkwang University Hospital.

Supplementary Figure S18B. Comparative risks of secondary outcomes between PEN–BLis and 3GCs among hospitalized COVID-19 patients: sensitivity analysis with expanded severity covariates

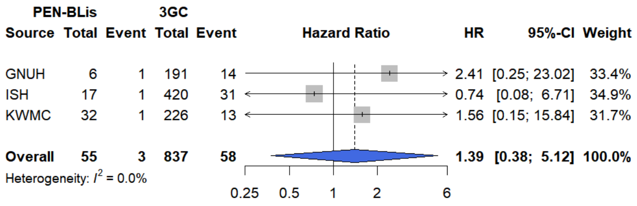

(a)

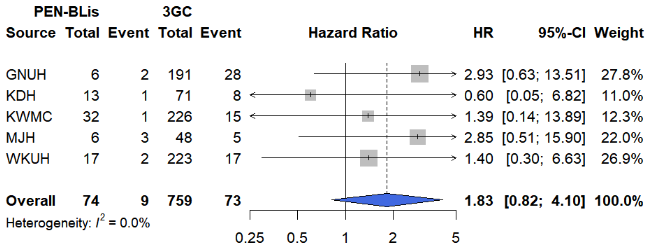

(b)

(a) Mechanical ventilation. (b) Vasopressor use. The number of events and total patients in each treatment group are shown alongside site-specific hazard ratios (HRs). The HRs were calibrated based on the empirical null distribution derived from negative control outcomes to account for systematic bias. The size of the data marker indicates the weight of the study. Error bars indicate 95% confidence intervals (CIs). 3GCs, Third-generation cephalosporins; GNUH, Gyeongsang National University Hospital; ISH, International St. Mary's Hospital; KDH, Kangdong Sacred Heart Hospital; KWMC, Kangwon National University Medical Center; MJH, Myongji Hospital; PEN–BLis, aminopenicillin/β-lactamase inhibitor combinations; WKUH, Wonkwang University Hospital.

Supplementary Figure S19A. Comparative risks of primary outcomes between fluoroquinolones and 3GCs among hospitalized COVID-19 patients: after excluding patients with early positive cultures or elevated baseline procalcitonin

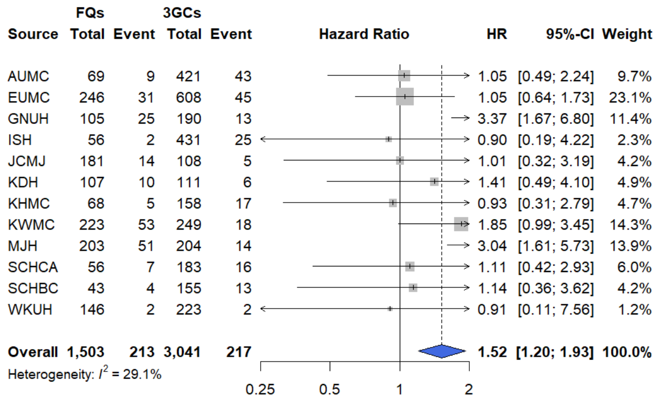

(a)

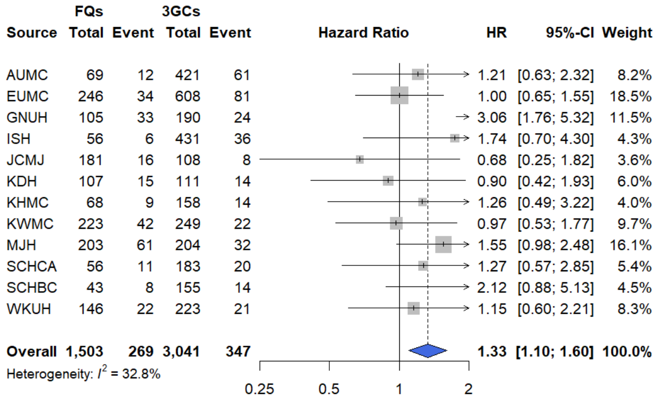

(b)

(a) All-cause in-hospital mortality. (b) Composite outcome. The number of events and total patients in each treatment group are shown alongside site-specific hazard ratios (HRs). The HRs were calibrated based on the empirical null distribution derived from negative control outcomes to account for systematic bias. The size of the data marker indicates the weight of the study. Error bars indicate 95% confidence intervals (CIs). 3GCs, Third-generation cephalosporins; AUMC, Ajou University Medical Center; EUMC, Ewha Womans University Medical Center; FQs, Fluoroquinolones; GNUH, Gyeongsang National University Hospital; ISH, International St. Mary's Hospital; JCMJ, Jecheon Myongji Hospital; KDH, Kangdong Sacred Heart Hospital; KHMC, Kyunghee University Medical Center; KWMC, Kangwon National University Medical Center; MJH, Myongji Hospital; SCHCA, Soonchunhyang University Hospital Cheonan Center; SCHBC, Soonchunhyang University Hospital Bucheon Center; WKUH, Wonkwang University Hospital.

Supplementary Figure S19B. Comparative risks of primary outcomes between PEN–BLis and 3GCs among hospitalized COVID-19 patients: after excluding patients with early positive cultures or elevated baseline procalcitonin

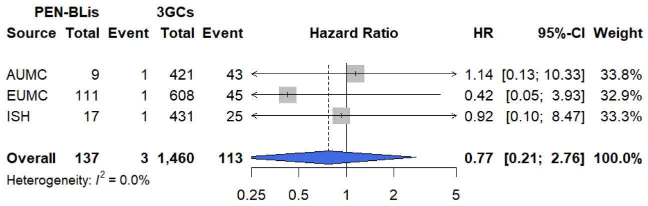

(a)

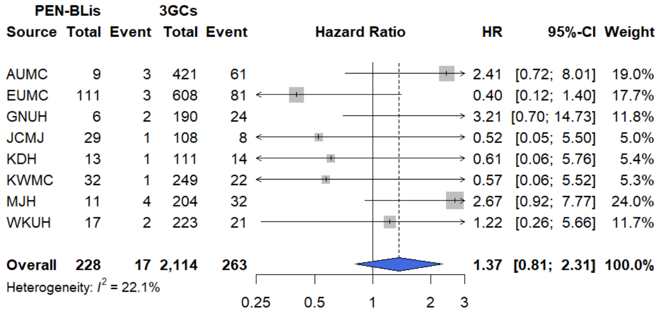

(b)

(a) All-cause in-hospital mortality. (b) Composite outcome. The number of events and total patients in each treatment group are shown alongside site-specific hazard ratios (HRs). The HRs were calibrated based on the empirical null distribution derived from negative control outcomes to account for systematic bias. The size of the data marker indicates the weight of the study. Error bars indicate 95% confidence intervals (CIs). 3GCs, Third-generation cephalosporins; AUMC, Ajou University Medical Center; EUMC, Ewha Womans University Medical Center; GNUH, Gyeongsang National University Hospital; ISH, International St. Mary's Hospital; JCMJ, Jecheon Myongji Hospital; KDH, Kangdong Sacred Heart Hospital; KWMC, Kangwon National University Medical Center; MJH, Myongji Hospital; PEN–BLis, aminopenicillin/β-lactamase inhibitor combinations; WKUH, Wonkwang University Hospital.

Supplementary Figure S20A. Comparative risks of secondary outcomes between fluoroquinolones and 3GCs among hospitalized COVID-19 patients: after excluding patients with early positive cultures or elevated baseline procalcitonin

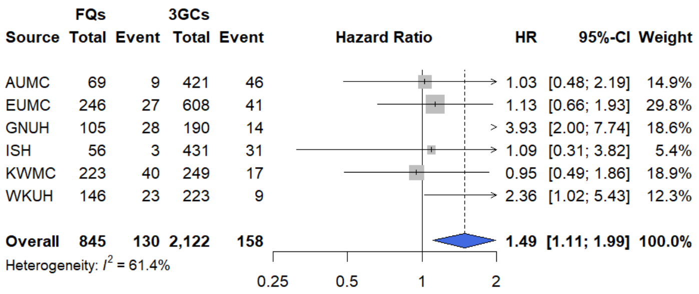

(a)

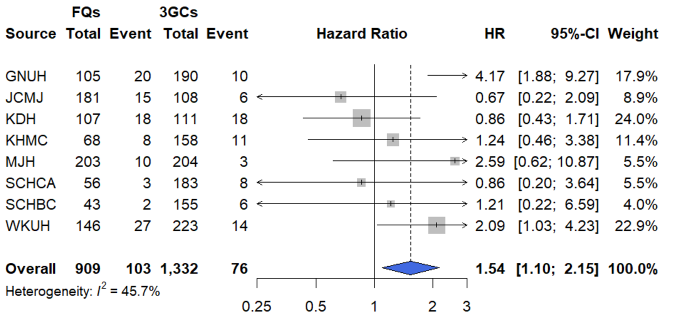

(b)

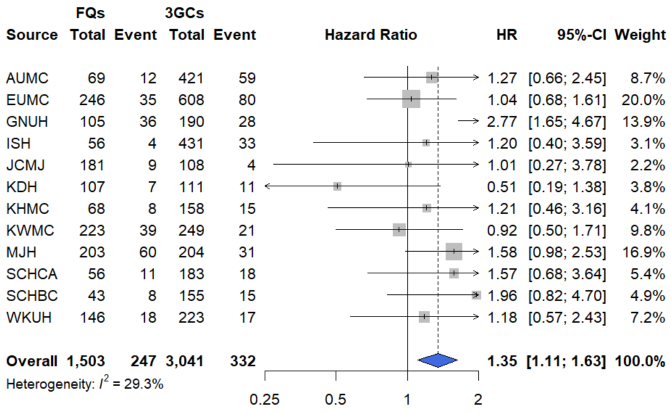

(c)

(a) Mechanical ventilation. (b) Intensive Care Unit (ICU) admission. (c) Vasopressor use. The number of events and total patients in each treatment group are shown alongside site-specific hazard ratios (HRs). The HRs were calibrated based on the empirical null distribution derived from negative control outcomes to account for systematic bias. The size of the data marker indicates the weight of the study. Error bars indicate 95% confidence intervals (CIs). 3GCs, Third-generation cephalosporins; AUMC, Ajou University Medical Center; EUMC, Ewha Womans University Medical Center; FQs, Fluoroquinolones; GNUH, Gyeongsang National University Hospital; ISH, International St. Mary's Hospital; JCMJ, Jecheon Myongji Hospital; KDH, Kangdong Sacred Heart Hospital; KHMC, Kyunghee University Medical Center; KWMC, Kangwon National University Medical Center; MJH, Myongji Hospital; SCHCA, Soonchunhyang University Hospital Cheonan Center; SCHBC, Soonchunhyang University Hospital Bucheon Center; WKUH, Wonkwang University Hospital.

Supplementary Figure S20B. Comparative risks of secondary outcomes between PEN–BLis and 3GCs among hospitalized COVID-19 patients: after excluding patients with early positive cultures or elevated baseline procalcitonin

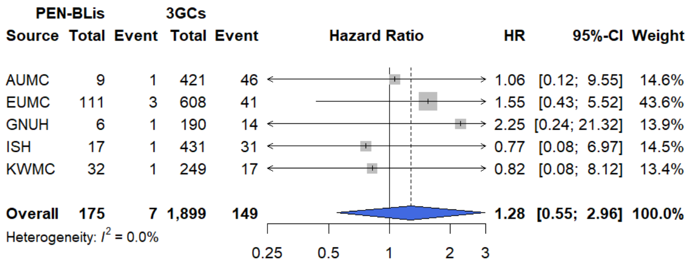

(a)

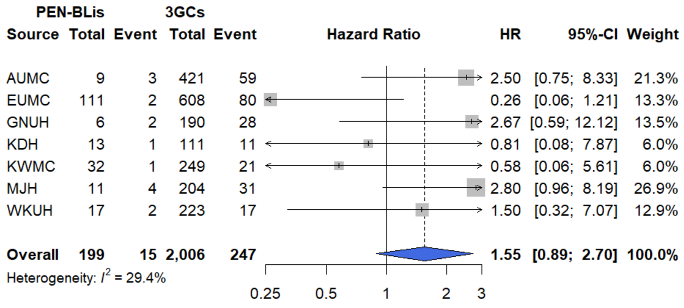

(b)

(a) Mechanical ventilation. (b) Vasopressor use. The number of events and total patients in each treatment group are shown alongside site-specific hazard ratios (HRs). The HRs were calibrated based on the empirical null distribution derived from negative control outcomes to account for systematic bias. The size of the data marker indicates the weight of the study. Error bars indicate 95% confidence intervals (CIs). 3GCs, Third-generation cephalosporins; AUMC, Ajou University Medical Center; EUMC, Ewha Womans University Medical Center; GNUH, Gyeongsang National University Hospital; ISH, International St. Mary's Hospital; JCMJ, Jecheon Myongji Hospital; KDH, Kangdong Sacred Heart Hospital; KWMC, Kangwon National University Medical Center; MJH, Myongji Hospital; PEN–BLis, aminopenicillin/β-lactamase inhibitor combinations; WKUH, Wonkwang University Hospital.

Supplementary Figure S21A. Comparative risks of primary outcomes between fluoroquinolones and 3GCs among hospitalized COVID-19 patients: during the Omicron period

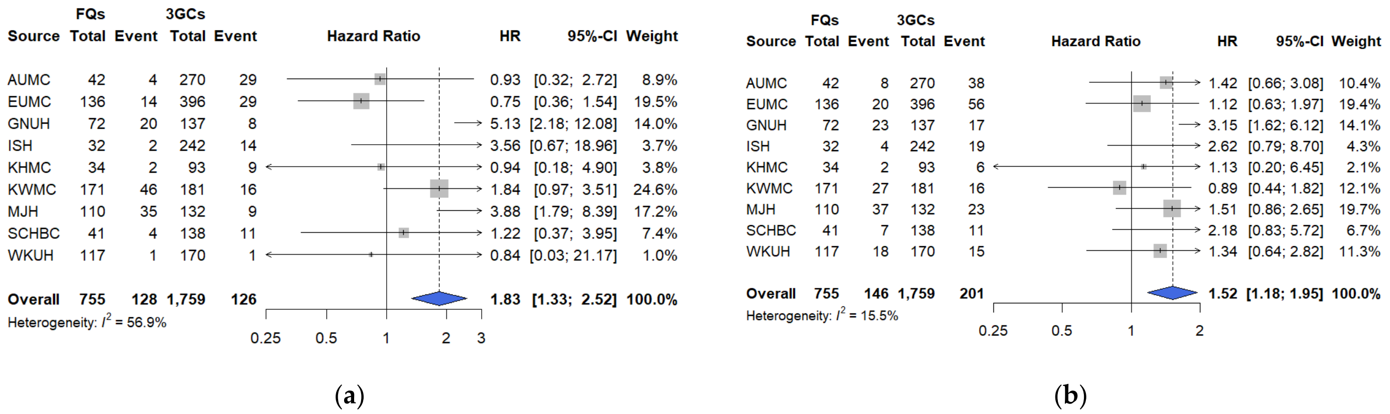

(a) All-cause in-hospital mortality. (b) Composite outcome. The number of events and total patients in each treatment group are shown alongside site-specific hazard ratios (HRs). The HRs were calibrated based on the empirical null distribution derived from negative control outcomes to account for systematic bias. The size of the data marker indicates the weight of the study. Error bars indicate 95% confidence intervals (CIs). 3GCs, Third-generation cephalosporins; AUMC, Ajou University Medical Center; EUMC, Ewha Womans University Medical Center; FQs, Fluoroquinolones; GNUH, Gyeongsang National University Hospital; ISH, International St. Mary's Hospital; JCMJ, Jecheon Myongji Hospital; KDH, Kangdong Sacred Heart Hospital; KHMC, Kyunghee University Medical Center; KWMC, Kangwon National University Medical Center; MJH, Myongji Hospital; SCHCA, Soonchunhyang University Hospital Cheonan Center; SCHBC, Soonchunhyang University Hospital Bucheon Center; WKUH, Wonkwang University Hospital.

Supplementary Figure S21B. Comparative risks of primary outcomes between PEN–BLis and 3GCs among hospitalized COVID-19 patients: during the Omicron period

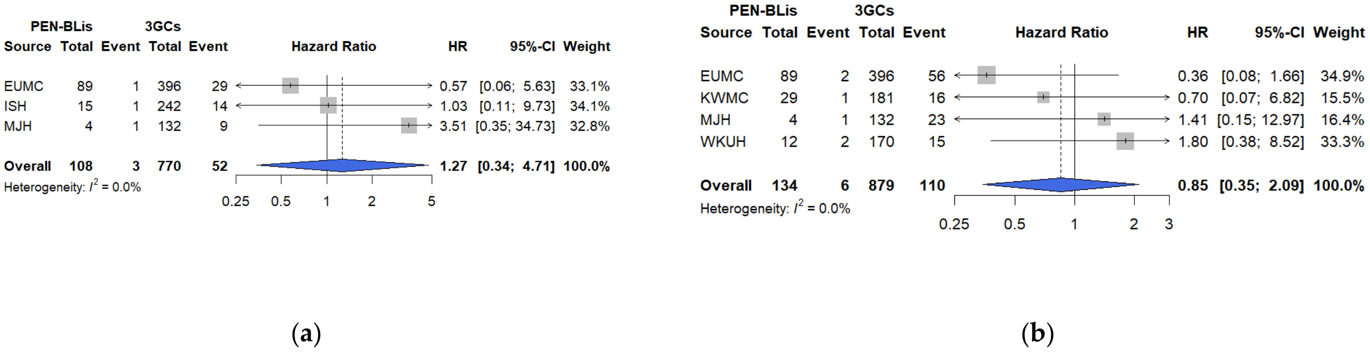

(a) All-cause in-hospital mortality. (b) Composite outcome. The number of events and total patients in each treatment group are shown alongside site-specific hazard ratios (HRs). The HRs were calibrated based on the empirical null distribution derived from negative control outcomes to account for systematic bias. The size of the data marker indicates the weight of the study. Error bars indicate 95% confidence intervals (CIs). 3GCs, Third-generation cephalosporins; AUMC, Ajou University Medical Center; EUMC, Ewha Womans University Medical Center; GNUH, Gyeongsang National University Hospital; ISH, International St. Mary's Hospital; JCMJ, Jecheon Myongji Hospital; KDH, Kangdong Sacred Heart Hospital; KWMC, Kangwon National University Medical Center; MJH, Myongji Hospital; PEN–BLis, aminopenicillin/β-lactamase inhibitor combinations; WKUH, Wonkwang University Hospital.

Supplementary Figure S22A. Comparative risks of secondary outcomes between fluoroquinolones and 3GCs among hospitalized COVID-19 patients: during the Omicron period

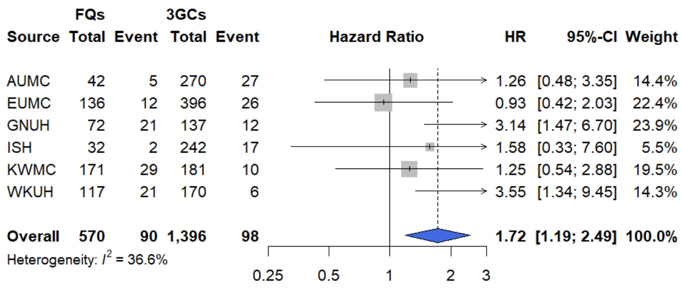

(a)

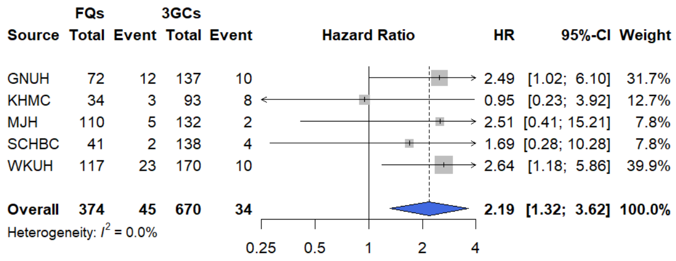

(b)

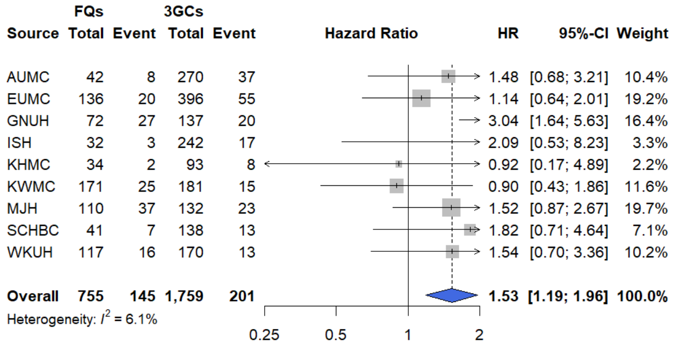

(c)

(a) Mechanical ventilation. (b) Intensive Care Unit (ICU) admission. (c) Vasopressor use. The number of events and total patients in each treatment group are shown alongside site-specific hazard ratios (HRs). The HRs were calibrated based on the empirical null distribution derived from negative control outcomes to account for systematic bias. The size of the data marker indicates the weight of the study. Error bars indicate 95% confidence intervals (CIs). 3GCs, Third-generation cephalosporins; AUMC, Ajou University Medical Center; EUMC, Ewha Womans University Medical Center; FQs, Fluoroquinolones; GNUH, Gyeongsang National University Hospital; ISH, International St. Mary's Hospital; JCMJ, Jecheon Myongji Hospital; KDH, Kangdong Sacred Heart Hospital; KHMC, Kyunghee University Medical Center; KWMC, Kangwon National University Medical Center; MJH, Myongji Hospital; SCHCA, Soonchunhyang University Hospital Cheonan Center; SCHBC, Soonchunhyang University Hospital Bucheon Center; WKUH, Wonkwang University Hospital.

Supplementary Figure S22B. Comparative risks of secondary outcomes between PEN–BLis and 3GCs among hospitalized COVID-19 patients: during the Omicron period

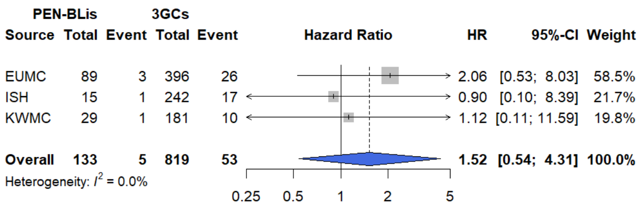

(a)

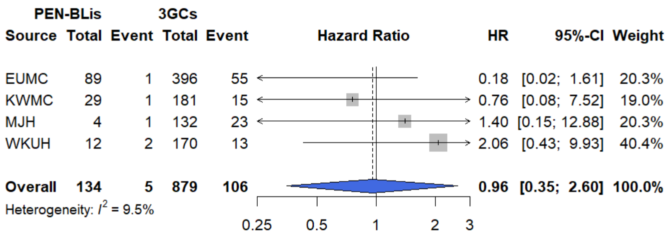

(b)

(a) Mechanical ventilation. (b) Vasopressor use. The number of events and total patients in each treatment group are shown alongside site-specific hazard ratios (HRs). The HRs were calibrated based on the empirical null distribution derived from negative control outcomes to account for systematic bias. The size of the data marker indicates the weight of the study. Error bars indicate 95% confidence intervals (CIs). 3GCs, Third-generation cephalosporins; AUMC, Ajou University Medical Center; EUMC, Ewha Womans University Medical Center; GNUH, Gyeongsang National University Hospital; ISH, International St. Mary's Hospital; JCMJ, Jecheon Myongji Hospital; KDH, Kangdong Sacred Heart Hospital; KWMC, Kangwon National University Medical Center; MJH, Myongji Hospital; PEN–BLis, aminopenicillin/β-lactamase inhibitor combinations; WKUH, Wonkwang University Hospital.

Supplementary Figure S23. Comparative risks of primary outcomes between ceftriaxone and levofloxacin/moxifloxacin among hospitalized COVID-19 patients

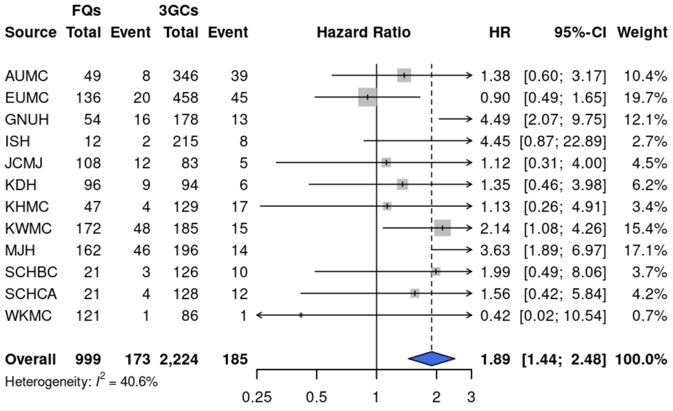

(a)

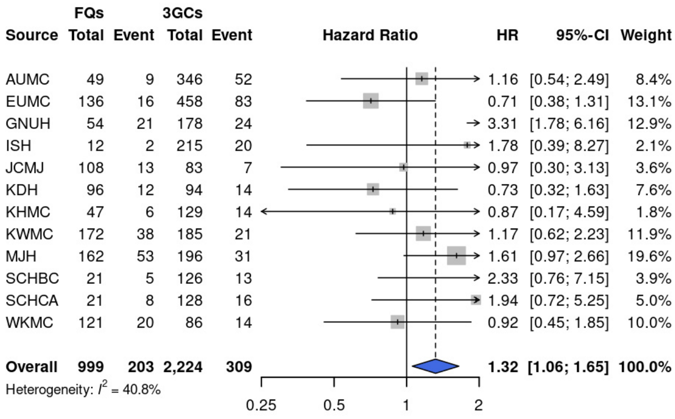

(b)

(a) All-cause in-hospital mortality. (b) Composite outcome. The number of events and total patients in each treatment group are shown alongside site-specific hazard ratios (HRs). The HRs were calibrated based on the empirical null distribution derived from negative control outcomes to account for systematic bias. The size of the data marker indicates the weight of the study. Error bars indicate 95% confidence intervals (CIs). 3GCs, Third-generation cephalosporins; AUMC, Ajou University Medical Center; EUMC, Ewha Womans University Medical Center; FQs, Fluoroquinolones; GNUH, Gyeongsang National University Hospital; ISH, International St. Mary's Hospital; JCMJ, Jecheon Myongji Hospital; KDH, Kangdong Sacred Heart Hospital; KHMC, Kyunghee University Medical Center; KWMC, Kangwon National University Medical Center; MJH, Myongji Hospital; SCHCA, Soonchunhyang University Hospital Cheonan Center; SCHBC, Soonchunhyang University Hospital Bucheon Center; WKUH, Wonkwang University Hospital.

Supplementary Figure S24. Comparative risks of secondary outcomes between ceftriaxone and levofloxacin/moxifloxacin among hospitalized COVID-19 patients

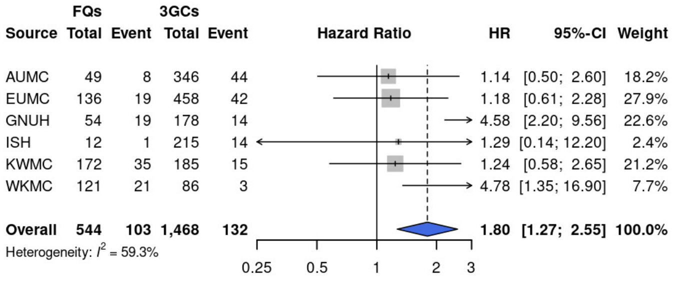

(a)

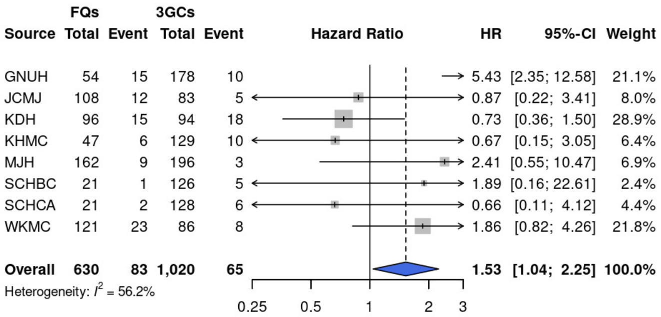

(b)

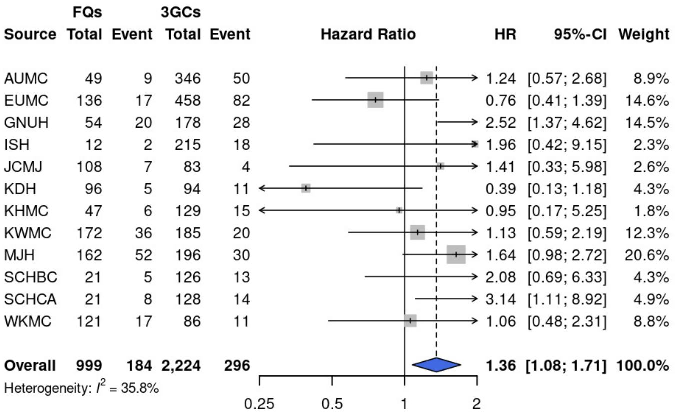

(c)

(a) Mechanical ventilation. (b) Intensive Care Unit (ICU) admission. (c) Vasopressor use. The number of events and total patients in each treatment group are shown alongside site-specific hazard ratios (HRs). The HRs were calibrated based on the empirical null distribution derived from negative control outcomes to account for systematic bias. The size of the data marker indicates the weight of the study. Error bars indicate 95% confidence intervals (CIs). 3GCs, Third-generation cephalosporins; AUMC, Ajou University Medical Center; EUMC, Ewha Womans University Medical Center; FQs, Fluoroquinolones; GNUH, Gyeongsang National University Hospital; ISH, International St. Mary's Hospital; JCMJ, Jecheon Myongji Hospital; KDH, Kangdong Sacred Heart Hospital; KHMC, Kyunghee University Medical Center; KWMC, Kangwon National University Medical Center; MJH, Myongji Hospital; SCHCA, Soonchunhyang University Hospital Cheonan Center; SCHBC, Soonchunhyang University Hospital Bucheon Center; WKUH, Wonkwang University Hospital.

Supplementary Figure S25A. Comparative risks of primary outcomes between fluoroquinolones and 3GCs among hospitalized COVID-19 patients: as-treated analysis

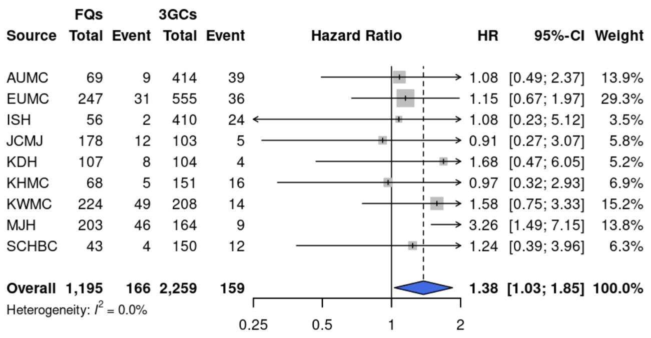

(a)

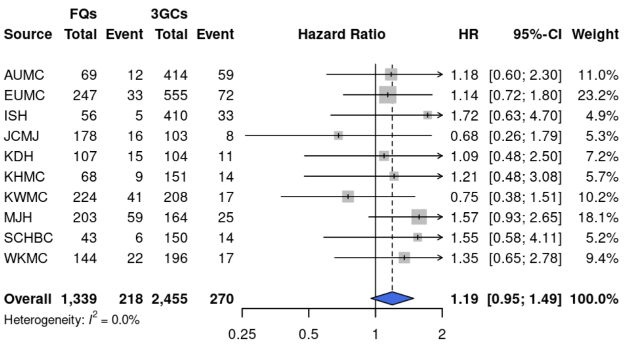

(b)

(a) All-cause in-hospital mortality. (b) Composite outcome. The number of events and total patients in each treatment group are shown alongside site-specific hazard ratios (HRs). The HRs were calibrated based on the empirical null distribution derived from negative control outcomes to account for systematic bias. The size of the data marker indicates the weight of the study. Error bars indicate 95% confidence intervals (CIs). 3GCs, Third-generation cephalosporins; AUMC, Ajou University Medical Center; EUMC, Ewha Womans University Medical Center; FQs, Fluoroquinolones; GNUH, Gyeongsang National University Hospital; ISH, International St. Mary's Hospital; JCMJ, Jecheon Myongji Hospital; KDH, Kangdong Sacred Heart Hospital; KHMC, Kyunghee University Medical Center; KWMC, Kangwon National University Medical Center; MJH, Myongji Hospital; SCHCA, Soonchunhyang University Hospital Cheonan Center; SCHBC, Soonchunhyang University Hospital Bucheon Center; WKUH, Wonkwang University Hospital.

Supplementary Figure S25B. Comparative risks of primary outcomes between PEN–BLis and 3GCs among hospitalized COVID-19 patients: as-treated analysis

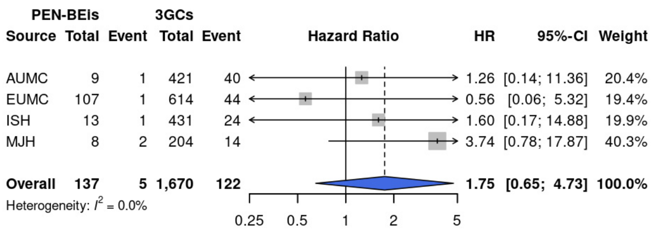

(a)

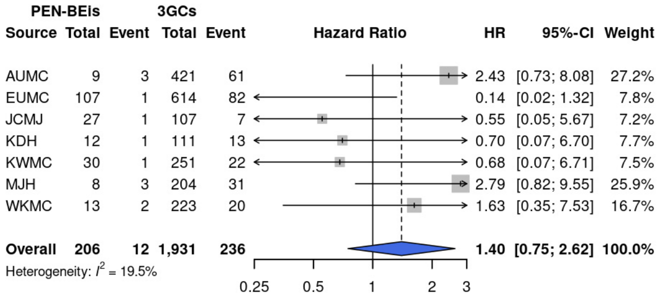

(b)

(a) All-cause in-hospital mortality. (b) Composite outcome. The number of events and total patients in each treatment group are shown alongside site-specific hazard ratios (HRs). The HRs were calibrated based on the empirical null distribution derived from negative control outcomes to account for systematic bias. The size of the data marker indicates the weight of the study. Error bars indicate 95% confidence intervals (CIs). 3GCs, Third-generation cephalosporins; AUMC, Ajou University Medical Center; EUMC, Ewha Womans University Medical Center; GNUH, Gyeongsang National University Hospital; ISH, International St. Mary's Hospital; JCMJ, Jecheon Myongji Hospital; KDH, Kangdong Sacred Heart Hospital; KWMC, Kangwon National University Medical Center; MJH, Myongji Hospital; PEN–BLis, aminopenicillin/β-lactamase inhibitor combinations; WKUH, Wonkwang University Hospital.

Supplementary Figure S26A. Comparative risks of secondary outcomes between fluoroquinolones and 3GCs among hospitalized COVID-19 patients: as-treated analysis

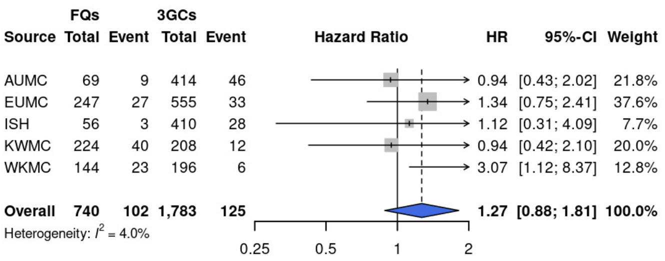

(a)

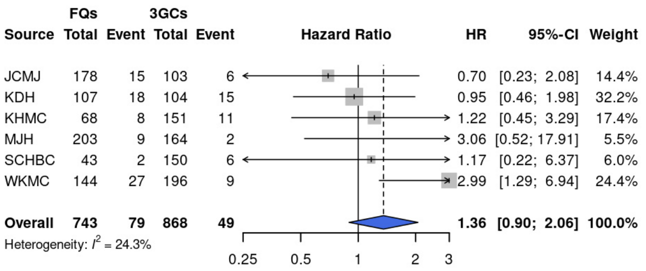

(b)

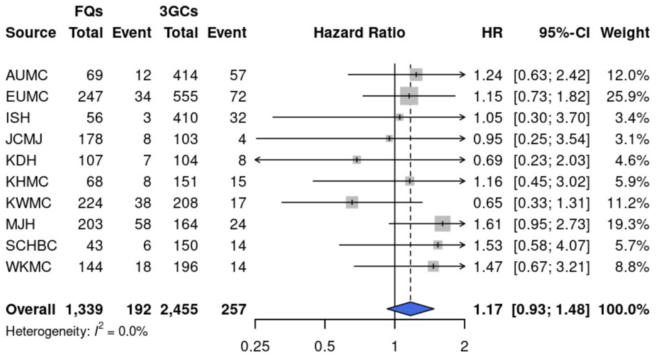

(c)

(a) Mechanical ventilation. (b) Intensive Care Unit (ICU) admission. (c) Vasopressor use. The number of events and total patients in each treatment group are shown alongside site-specific hazard ratios (HRs). The HRs were calibrated based on the empirical null distribution derived from negative control outcomes to account for systematic bias. The size of the data marker indicates the weight of the study. Error bars indicate 95% confidence intervals (CIs). 3GCs, Third-generation cephalosporins; AUMC, Ajou University Medical Center; EUMC, Ewha Womans University Medical Center; FQs, Fluoroquinolones; GNUH, Gyeongsang National University Hospital; ISH, International St. Mary's Hospital; JCMJ, Jecheon Myongji Hospital; KDH, Kangdong Sacred Heart Hospital; KHMC, Kyunghee University Medical Center; KWMC, Kangwon National University Medical Center; MJH, Myongji Hospital; SCHCA, Soonchunhyang University Hospital Cheonan Center; SCHBC, Soonchunhyang University Hospital Bucheon Center; WKUH, Wonkwang University Hospital.

Supplementary Figure S26B. Comparative risks of secondary outcomes between PEN–BLis and 3GCs among hospitalized COVID-19 patients: as-treated analysis

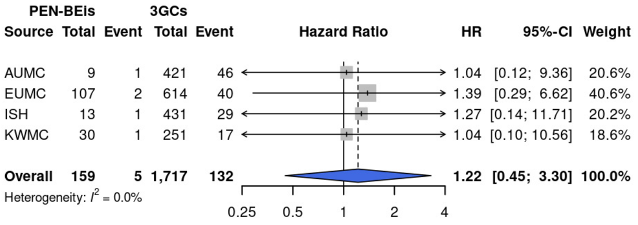

(a)

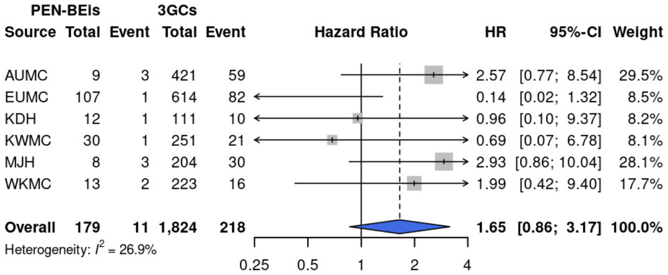

(b)

(a) Mechanical ventilation. (b) Vasopressor use. The number of events and total patients in each treatment group are shown alongside site-specific hazard ratios (HRs). The HRs were calibrated based on the empirical null distribution derived from negative control outcomes to account for systematic bias. The size of the data marker indicates the weight of the study. Error bars indicate 95% confidence intervals (CIs). 3GCs, Third-generation cephalosporins; AUMC, Ajou University Medical Center; EUMC, Ewha Womans University Medical Center; GNUH, Gyeongsang National University Hospital; ISH, International St. Mary's Hospital; JCMJ, Jecheon Myongji Hospital; KDH, Kangdong Sacred Heart Hospital; KWMC, Kangwon National University Medical Center; MJH, Myongji Hospital; PEN–BLis, aminopenicillin/β-lactamase inhibitor combinations; WKUH, Wonkwang University Hospital.
